# Changes in the global hospitalisation burden of respiratory syncytial virus in young children during the COVID-19 pandemic: a systematic analysis

**DOI:** 10.1016/S1473-3099(23)00630-8

**Published:** 2023-12-20

**Authors:** Bingbing Cong, Uğurcan Koç, Teresa Bandeira, Quique Bassat, Louis Bont, Giorgi Chakhunashvili, Cheryl Cohen, Christine Desnoyers, Laura L Hammitt, Terho Heikkinen, Q Sue Huang, Joško Markić, Ainara Mira-Iglesias, Jocelyn Moyes, D James Nokes, Dominique Ploin, Euri Seo, Rosalyn Singleton, Nicole Wolter, Chee Fu Yung, Heather J Zar, Daniel R Feikin, Erin G Sparrow, Respiratory Virus Global Epidemiology Network, Harish Nair, You Li, PROMISE investigators

**Affiliations:** Department of Epidemiology, National Vaccine Innovation Platform, School of Public Health, Nanjing Medical University, Nanjing, China; Centre for Global Health, Usher Institute, University of Edinburgh, Edinburgh, UK; Pediatric Department, Hospital Santa Maria, Centro Hospitalar Universitário Lisboa Norte, Centro Académico de Medicina de Lisboa, University of Lisbon, Lisbon, Portugal; ISGlobal, Hospital Clínic–Universitat de Barcelona, Barcelona, Spain; Centro de Investigação em Saúde de Manhiça, Maputo, Mozambique; Catalan Institution for Research and Advanced Studies, Barcelona, Spain; Wilhelmina Children’s Hospital, University Medical Center Utrecht, Utrecht, Netherlands; ReSViNET Foundation, Zeist, Netherlands; National Center for Disease Control and Public Health, Tbilisi, Georgia; Centre for Respiratory Diseases and Meningitis, National Institute for Communicable Diseases, Division of the National Health Laboratory Service, Johannesburg, South Africa; School of Public Health, Faculty of Health Sciences, University of the Witwatersrand, Johannesburg, South Africa; Yukon-Kuskokwim Health Corporation, Bethel, AK, USA; Department of International Health, Johns Hopkins Bloomberg School of Public Health, Baltimore, MD, USA; Department of Pediatrics, University of Turku and Turku University Hospital, Turku, Finland; WHO National Influenza Centre, Institute of Environmental Science and Research, Wellington, New Zealand; Department of Pediatrics, University Hospital Split, Split, Croatia; University of Split School of Medicine, Split, Croatia; Área de Investigación en Vacunas, Fundación para el Fomento de la Investigación Sanitaria y Biomédica de la Comunitat Valenciana, Salud Pública, Valencia, Spain; CIBER de Epidemiología y Salud Pública, Instituto de Salud Carlos III, Madrid, Spain; Centre for Respiratory Diseases and Meningitis, National Institute for Communicable Diseases, Division of the National Health Laboratory Service, Johannesburg, South Africa; Kenya Medical Research Institute–Wellcome Trust Research Programme, Kilifi, Kenya; School of Life Sciences, University of Warwick, Coventry, UK; Hospices Civils de Lyon, Hôpital Femme Mère Enfant, Service de Réanimation Pédiatrique et d’Accueil des Urgences, Bron, France; The Center for Viral Immunology, Korea Virus Research Institute, Institute for Basic Science, Daejeon, South Korea; Alaska Native Tribal Health Consortium, Anchorage, AK, USA; Centre for Respiratory Diseases and Meningitis, National Institute for Communicable Diseases, Division of the National Health Laboratory Service, Johannesburg, South Africa; School of Pathology, Faculty of Health Sciences, University of the Witwatersrand, Johannesburg, South Africa; Infectious Diseases Service, Department of Paediatrics, KK Women’s and Children’s Hospital, Singapore; Duke–NUS Medical School, Singapore; Lee Kong Chian School of Medicine, Nanyang Technological University, Singapore; Department of Paediatrics and Child Health, Red Cross War Memorial Children’s Hospital, Cape Town, South Africa; South African Medical Research Council Unit on Child & Adolescent Health, University of Cape Town, Cape Town, South Africa; Department of Immunization, Vaccines, and Biologicals, WHO, Geneva, Switzerland; Department of Immunization, Vaccines, and Biologicals, WHO, Geneva, Switzerland; Department of Epidemiology, National Vaccine Innovation Platform, School of Public Health, Nanjing Medical University, Nanjing, China; Centre for Global Health, Usher Institute, University of Edinburgh, Edinburgh, UK; School of Public Health, Faculty of Health Sciences, University of the Witwatersrand, Johannesburg, South Africa; Department of Epidemiology, National Vaccine Innovation Platform, School of Public Health, Nanjing Medical University, Nanjing, China; Centre for Global Health, Usher Institute, University of Edinburgh, Edinburgh, UK

## Abstract

**Background:**

The COVID-19 pandemic is reported to have affected the epidemiology of respiratory syncytial virus (RSV), which could have important implications for RSV prevention and control strategies. We aimed to assess the hospitalisation burden of RSV-associated acute lower respiratory infection (ALRI) in children younger than 5 years during the pandemic period and the possible changes in RSV epidemiology from a global perspective.

**Methods:**

We conducted a systematic literature search for studies published between Jan 1, 2020, and June 30, 2022, in MEDLINE, Embase, Global Health, Web of Science, the WHO COVID-19 Research Database, CINAHL, LILACS, OpenGrey, CNKI, WanFang, and CqVip. We included unpublished data on RSV epidemiology shared by international collaborators. Eligible studies reported data on at least one of the following measures for children (aged <5 years) hospitalised with RSV-associated ALRI: hospital admission rates, in-hospital case fatality ratio, and the proportion of hospitalised children requiring supplemental oxygen or requiring mechanical ventilation or admission to intensive care. We used a generalised linear mixed-effects model for data synthesis to measure the changes in the incidence, age distribution, and disease severity of children hospitalised with RSV-associated ALRI during the pandemic, compared with the year 2019.

**Findings:**

We included 61 studies from 19 countries, of which 14 (23%) studies were from the published literature (4052 identified records) and 47 (77%) were from unpublished datasets. Most (51 [84%]) studies were from high-income countries; nine (15%) were from upper-middle-income countries, one (2%) was from a lower-middle-income country (Kenya), and none were from a low-income country. 15 studies contributed to the estimates of hospitalisation rate and 57 studies contributed to the severity analyses. Compared with 2019, the rates of RSV-associated ALRI hospitalisation in all children (aged 0–60 months) in 2020 decreased by 79·7% (325 000 cases *vs* 66 000 cases) in high-income countries, 13·8% (581 000 cases *vs* 501 000 cases) in upper-middle-income countries, and 42·3% (1 378 000 cases *vs* 795 000 cases) in Kenya. In high-income countries, annualised rates started to rise in 2021, and by March, 2022, had returned to a level similar to 2019 (6·0 cases per 1000 children [95% uncertainty interval 5·4–6·8] in April, 2021, to March, 2022, *vs* 5·0 cases per 1000 children [3·6–6·8] in 2019). By contrast, in middle-income countries, rates remained lower in the latest period with data available than in 2019 (for upper-middle-income countries, 2·1 cases [0·7–6·1] in April, 2021, to March, 2022, *vs* 3·4 [1·2–9·7] in 2019; for Kenya, 2·2 cases [1·8–2·7] in 2021 *vs* 4·1 [3·5–4·7] in 2019). Across all time periods and income regions, hospitalisation rates peaked in younger infants (aged 0 to <3 months) and decreased with increasing age. A significantly higher proportion of children aged 12–24 months were hospitalised with RSV-associated ALRI in high-income and upper-middle-income countries during the pandemic years than in 2019, with odds ratios ranging from 1·30 (95% uncertainty interval 1·07–1·59) to 2·05 (1·66–2·54). No consistent changes in disease severity were observed.

**Interpretation:**

The hospitalisation burden of RSV-associated ALRI in children younger than 5 years was significantly reduced during the first year of the COVID-19 pandemic. The rebound in hospitalisation rates to pre-pandemic rates observed in the high-income region but not in the middle-income region by March, 2022, suggests a persistent negative impact of the pandemic on health-care systems and health-care access in the middle-income region. RSV surveillance needs to be established (or re-established) to monitor changes in RSV epidemiology, particularly in low-income and lower-middle-income countries.

**Funding:**

EU Innovative Medicines Initiative Preparing for RSV Immunisation and Surveillance in Europe (PROMISE), Bill & Melinda Gates Foundation, and WHO.

## Introduction

Human respiratory syncytial virus (RSV) is a leading cause of acute lower respiratory infection (ALRI) in infants and young children.^1,2^ We previously estimated that, globally in 2019, there were 33·0 million episodes, 3·6 million hospital admissions, 26 300 in-hospital deaths, and approximately 101 000 overall deaths from RSV-associated ALRI in children younger than 5 years.^2^ Compared with high-income countries, low-income and middle-income countries (LMICs) had a higher incidence of RSV-associated ALRI but similar or lower rates of hospitalisation, suggesting scarce health-care accessibility and availability in these settings.^2^ There have been major advances in the development of novel RSV prophylactic products targeting infants; long-acting monoclonal antibody and maternal vaccine products were licensed in the USA and Europe from late 2022 to mid 2023.^3^

Following the onset of the COVID-19 pandemic, low RSV activity was observed worldwide as a result of the large-scale implementation of non-pharmaceutical interventions (NPIs).^4–6^ RSV epidemics resurged as these NPIs were lifted.^7^ However, several studies have reported that RSV epidemiology might have been reshaped by the COVID-19 pandemic with regard to age distribution and disease severity, although these reported changes in epidemiological characteristics are not consistent across existing studies. Studies from France,^5^ Denmark,^8^ the USA,^9^ and Australia^10^ showed that children hospitalised with RSV disease during the COVID-19 pandemic tended to be older than those hospitalised during the pre-pandemic period; however, a study from Croatia^11^ did not observe any changes in the age distribution of hospitalised children. Regarding disease severity, some studies^11,12^ reported that the proportion of severe RSV cases during the pandemic period was higher than that of the pre-pandemic period, whereas other studies^13,14^ reported no differences in disease severity. Although these changes reflect the altered circulating patterns of RSV during the COVID-19 pandemic, they might also reflect possible changes in health-care systems in response to the pandemic. For instance, the observed changes in the burden of RSV hospitalisation could differ by country income group, considering that the adaptability and resilience of health-care systems under the impact of the COVID-19 pandemic might have varied depending on the attributes or structure of these systems.^15–17^

Understanding the changes in RSV epidemiology following the onset of the COVID-19 pandemic could have important implications for RSV prevention and control strategies in upcoming RSV seasons and provide further insight into the possible effects of the COVID-19 pandemic on health-care systems and health-care access. We aimed to assess the hospitalisation burden of RSV-associated ALRI in children younger than 5 years during the COVID-19 pandemic and to understand the possible changes in RSV epidemiology from a global perspective.

## Methods

### Search strategy and selection criteria

In this systematic analysis of published and unpublished epidemiological data on RSV, we conducted a systematic literature review to identify studies that reported the hospitalisation burden of RSV-associated ALRI in children younger than 5 years, following the onset of the COVID-19 pandemic. As described previously,^2^ ALRI was defined as a physician-confirmed diagnosis of pneumonia or bronchiolitis for hospital-based studies. RSV-associated ALRI was defined as ALRI with a laboratory-confirmed RSV infection in specimens from the upper respiratory tract. Although the onset of local COVID-19 epidemics varied worldwide, for ease of comparison and reporting, we considered Jan 1, 2020, as the onset of the COVID-19 pandemic.

We searched the following 11 electronic databases: MEDLINE (Ovid), Embase (Ovid), Web of Science, Global Health (Ovid), the WHO COVID-19 Research Database, CINAHL, LILACS, grey literature (OpenGrey) databases, CNKI, WanFang Data, and CqVip. We searched for randomised controlled trials, cross-sectional studies, and cohort studies reporting changes in the incidence, age distribution, and disease severity of children (aged <5 years) hospitalised with RSV-associated ALRI, published between Jan 1, 2020, and June 30, 2022, using a tailored search strategy adapted on the basis of our previous work (appendix pp 4–7).^2^ No language restrictions were applied. Eligible studies reported data on at least one of the following measures for children hospitalised with RSV-associated ALRI: hospital admission rates, in-hospital case fatality ratios (CFRs), or severity measures (ie, the proportion of hospitalised children requiring supplemental oxygen or requiring mechanical ventilation or admission to the intensive care unit [ICU]). These data had to be available for at least 12 consecutive months, except for data on severity measures. Studies were excluded if the case definition of RSV-associated ALRI was not clearly defined or consistently applied, if RSV infection was not confirmed in the laboratory or was confirmed solely from serology, or if no data were available after the onset of the COVID-19 pandemic. Both individual patient-level data and summary estimates were sought, although only summary estimates were identified from the published literature.

Two reviewers (BC and UK) screened the results of the literature search and extracted data independently using a tailored template for data extraction. This template collected study-level information, such as location or country, study period, eligibility criteria, age group, case definition, clinical specimen and diagnostic tests, and reported estimates of hospitalisation and mortality. Any discrepancies between the two reviewers during the data screening and extraction stages were arbitrated by a senior member of the review team (HN or YL).

To supplement data extracted from the systematic literature review, we wrote to members of the Respiratory Virus Global Epidemiology Network (RSV GEN)^2^ and invited them to contribute unpublished data on RSV. The call for data was initiated on March 26, 2021, and we aimed to collect monthly aggregated data on RSV-associated ALRI hospitalisations between Jan 1, 2019, and May 31, 2022, by age group (0 to <3 months, 3 to <6 months, 6 to <9 months, 9 to <12 months, 12 to <18 months, 18 to <24 months, and 24 to <60 months). This level of granularity allowed us to analyse how the hospitalisation burden of RSV might have changed over time during the course of the pandemic and how these changes might have varied by age group. The complete list of unpublished datasets contributed by RSV GEN members is presented in the appendix (p 9).

For both published and unpublished data on RSV, two reviewers (BC and UK) assessed study quality using a quality scoring form identical to that used in our previous reviews.^2,18^ Briefly, the quality scoring form assessed study quality and risk of bias on the basis of study design, participants, case definition, sampling strategy (for RSV testing), and diagnostic tests. For studies reporting hospitalisation rate, adjustment for health-care use was also assessed (appendix p 8). We calculated the overall quality score, ranging between 0 (lowest quality) and 1 (highest quality) for each study after assessing each criterion listed above. Any discrepancies during the quality assessment were resolved after discussion within the review team. The protocol of this study is registered with PROSEPRO, CRD42022303344.

### Data analysis

For all data analyses, we used 2019 as the reference year of the pre-pandemic period. We also presented our previously published estimate of RSV disease burden for the year 2019 for comparison, which was based on data up to and including 2019.^2^ Given that RSV epidemiology could change during the course of the COVID-19 pandemic, we further divided the pandemic period into the following three 12-month periods, where data allowed: the year 2020, the year 2021, and the latest available period between April, 2021, and March, 2022 (determined on the basis of data availability).

We synthesised the annualised rate of RSV-associated ALRI hospitalisations by region (World Bank income classification for 2019 and country development status according to UNICEF categories^19,20^), age group, and time period (2019, 2020, 2021, or latest available period). For this synthesis, we conducted a generalised linear mixed-effects model meta-analysis^21^ using a Poisson regression model of RSV hospitalisation counts, with a random study-level intercept and an offset of person-time. Similar to our previous work,^2,11^ to account for undertesting of RSV, the rate of RSV-associated hospitalisation was adjusted upwards by applying the proportion of children testing positive for RSV among children with ALRI tested for RSV to the total number of tested and untested children with ALRI for each combination of age group and calendar month (to account for RSV seasonality, changes in testing practices, and variations in RSV positivity rate), if this adjustment was not already done in the individual study. The number of RSV-associated ALRI hospitalisations in each region and year was calculated with population estimates for the corresponding years from World Population Prospects.^22^ Global estimates were obtained by summing the numbers of developing and industrialised countries. For the main analysis, we included studies focusing on Indigenous populations in industrialised countries (eg, American Indian and Alaska Native children) and grouped them into upper-middle-income or developing countries, given that this population was considered to share similar socioeconomic and demographic risk factors for respiratory infections with populations in developing countries.^23^ We included and reclassified these studies as high-income or industrialised countries in a sensitivity analysis. We conducted a sensitivity analysis that excluded studies with a high risk of bias (defined as quality scores of <0·6 as in our previous work^2^) and a sensitivity analysis that excluded studies testing RSV only during the perceived RSV epidemic months. The same model used to estimate RSV hospitalisation counts was repeated to obtain the rate of children hospitalised with RSV-associated ALRI requiring mechanical ventilation or ICU admission.

To help understand the changes in population behaviours in response to NPIs and to interpret the changes in RSV epidemiology, we downloaded Google community mobility data,^24^ which documented changes in population mobility compared with a pre-defined period, and the Oxford COVID-19 Government Response Tracker Stringency Index of COVID-19 NPIs,^25^ a composite measure ranging from 0 (no intervention) to 100 (strictest intervention). For Google community mobility data, we selected the mobility metric of visits to retail and recreation places as the proxy of population mobility, given that this metric was previously shown to be associated with transmission of SARS-CoV-2.^26^ We calculated median and IQR values across the study sites of average mobility metrics and the NPI stringency index for each time period.

For a subset of individual studies with available monthly aggregated data on RSV-associated ALRI hospitalisations, we calculated the 12-month moving hospitalisation rate between January, 2019, and May, 2022, for each study and conducted a generalised linear mixed-effects model meta-analysis. The selection of a 12-month window accounted for the typical annual seasonality of RSV and allowed us to understand how the annualised hospitalisation rate of children with RSV-associated ALRI had changed during a finer timescale (ie, the 12-month interval moved month by month). As an exploratory analysis, we further calculated the 12-month moving average of population mobility for each country and conducted a cross-correlation analysis (Pearson’s correlation) between the 12-month hospitalisation rate and the 12-month moving average of population mobility, with a range of time lags between 0 months and 11 months. For each country, we identified the time lag with the highest correlation coefficient (which needed to be >0·5 to exclude weak correlations) as the optimal time lag between population mobility and hospitalisation rate. This time lag helped us to understand the temporal association between changes in population mobility and hospitalisation rate.

We selected the youngest age group (0 to <3 months) as the reference, considering that this age group was available in most studies included and could be used to test whether older age groups accounted for more RSV-associated ALRI hospitalisations relative to this younger age group. We calculated the odds ratio (OR) for observing RSV-associated ALRI hospitalisations in older age groups (ie, 3 to <6 months, 6 to <9 months, 9 to <12 months, and 12 to <24 months) during the pandemic period for each study, and then conducted a generalised linear mixed-effects model meta-analysis (using a logistic regression model) to obtain the pooled OR separately by region, age group, and time period (ie, 2020, 2021, and the latest available period). In addition, we restricted the analysis to hospitalised children requiring supplemental oxygen or requiring mechanical ventilation or ICU admission to examine whether age distribution changed specifically among severe cases of RSV-associated ALRI. We conducted sensitivity analyses that excluded studies with a high risk of bias or those without year-round testing for RSV.

For each study, we calculated the OR for observing severe outcomes (ie, the proportion of children requiring supplemental oxygen and the proportion requiring mechanical ventilation or ICU admission) among all children hospitalised with RSV-associated ALRI during the pandemic period. We then conducted a generalised linear mixed-effects model meta-analysis (using a logistic regression model) to obtain the pooled OR separately by severity outcome, age group, and time period (ie, 2020, 2021, and the latest available period). For these outcomes, we conducted a sensitivity analysis that excluded studies with a high risk of bias.

We obtained pooled meta-estimates of CFRs for children hospitalised with RSV-associated ALRI by income region, age group, and time period (2019 and 2020 onwards; no further stratification was performed because of data scarcity) through a generalised linear mixed-effects model meta-analysis that was based on a logistic regression model with a random study-level intercept. We conducted a sensitivity analysis that excluded studies with a high risk of bias.

For estimates that were generated from a single meta-analysis, the uncertainty interval (UI) was calculated as follows: coefficient ±1·96 × SE. For estimates that were generated through results from multiple meta-analyses, the UI was generated with the Monte Carlo simulation to avoid inflation, which was based on 1000 samples of each meta-estimate from log-normal distributions, with the 2·5th percentile defining the lower bound and the 97·5th percentile defining the upper bound.^27^

All statistical analyses were done with R (version 4.2.1). This study was reported according to PRISMA guidelines (appendix pp 58–60) and in accordance with GATHER recommendations (appendix p 57). This study is registered with PROSPERO, number CRD42022303344.

### Role of the funding source

The funders of the study had no role in study design, data collection, data analysis, data interpretation, or writing of the report.

## Results

**Figure 1:**
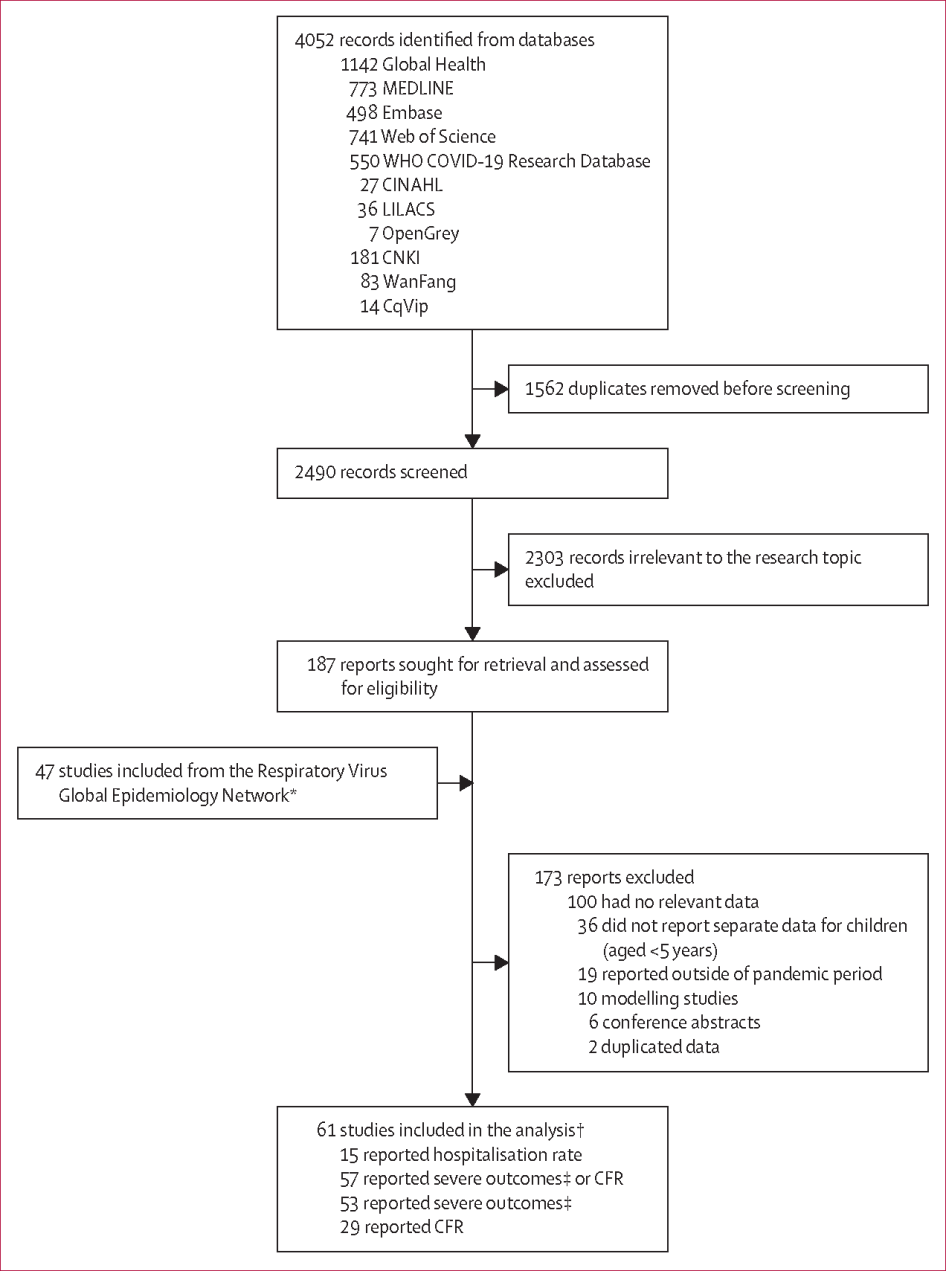
Study selection CFR=case fatality ratio. RSV=respiratory syncytial virus. ALRI=acute lower respiratory infection. *Details in the appendix (p 9). †Studies could have contributed data to more than one category. ‡Defined as the proportion of children hospitalised with RSV-associated ALRI requiring supplemental oxygen or requiring mechanical ventilation or admission to intensive care.

We identified 4052 records from the systematic literature search of databases, of which 2490 (61·5%) studies were screened and a total of 14 (0·3%) from seven countries were included (figure 1). We also included unpublished data from 47 study sites from 14 countries, bringing the total number of included studies to 61 from 19 countries. Of the 61 studies, 51 (84%) were from 13 high-income countries, nine (15%) were from five upper-middle-income countries, one (2%) was from a lower-middle-income country (Kenya), and none were from the low-income region. 15 (25%) studies contributed to the estimates of hospitalisation rate: three (5%) studies from Africa, three (5%) from the Western Pacific, four (7%) from North America, and five (8%) from Europe. A total of 57 (93%) studies from 18 countries contributed data on the proportion of severe cases or in-hospital CFR (figure 1). Basic characteristics and the geographical distribution of included studies are summarised in the appendix (pp 11–17, 38–39).

In 2020, all three income regions reported substantial decreases in the rate of children hospitalised with RSV-associated ALRI consistently across all age groups (table 1). Compared with 2019, the rates of hospitalisation among all children (aged 0 to <60 months) in 2020 decreased by 79·7% (325 000 cases *vs* 66 000 cases) in high-income countries, 13·8% (581 000 cases *vs* 501 000 cases) in upper-middle-income countries, and 42·3% (1 378 000 cases *vs* 795 000 cases) in Kenya (a lower-middle-income country). However, these rates started to rise in 2021 and showed different trajectories by income region. In the high-income region, the annualised rate of RSV-associated ALRI hospitalisations for April, 2021, to March, 2022, was 6·0 cases per 1000 children (95% UI 5·4–6·8), similar to the estimated rates of 5·0 cases per 1000 children (3·6–6·8) for 2019 in this study and 6·0 cases per 1000 children (4·7–7·7) for 2019 in our previous analysis.^2^ By contrast, in middle-income countries, annualised rates generally remained substantially lower in 2021 and in the latest available period than in 2019 (table 1). The only exception to this observation was for children aged 12 to <60 months in the lower-middle-income region (ie, data from Kenya), for whom the hospitalisation rate in 2021 (1·0 case per 1000 children [0·7–1·4]) was similar to the pre-pandemic rate (0·7 cases per 1000 children [0·5–1·0] in this study and 1·6 cases per 1000 children [1·0–2·7] from our previous analysis).^2^

**Figure 2:**
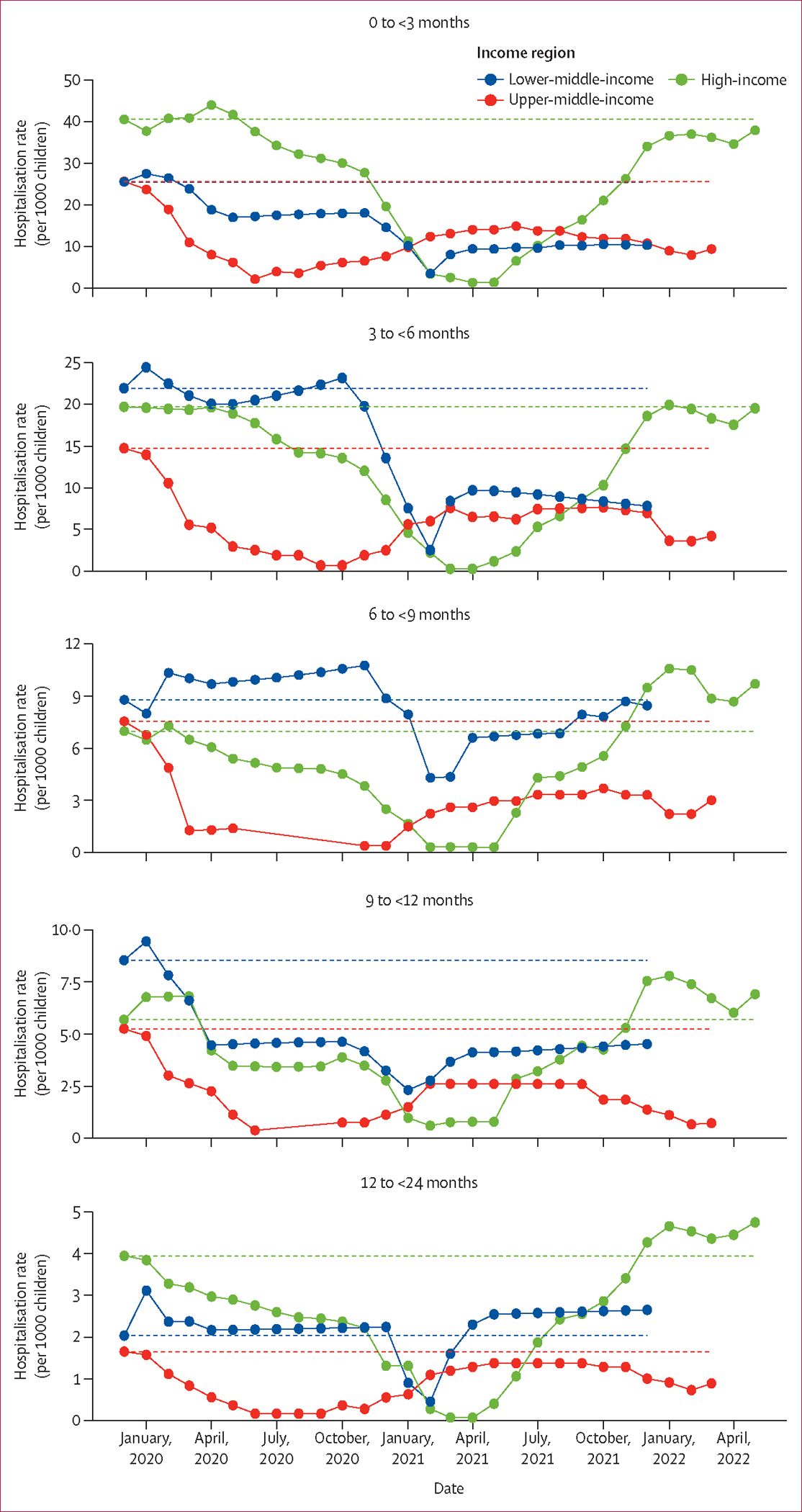
12-month rates of children hospitalised with RSV-associated ALRI by age group and World Bank income region The moving average curve is plotted according to each study (five studies from the high-income region, two from the upper-middle-income region, and one from the lower-middle-income region) reporting complete data (ie, hospitalisation rates from January, 2019, to March, 2022, or later). Each date represents the end of the 12-month interval—eg, January, 2020, represents the time period between February, 2019, and January, 2020. Dotted lines represent the corresponding hospitalisation rates in the pre-pandemic reference period (ie, the year 2019). RSV=respiratory syncytial virus. ALRI=acute lower respiratory infection.

Regional estimates stratified by country development status showed similar trends to the main analysis by country income regions; when extrapolating to global estimates, we estimated that 1·7 million (95% UI 0·8–4·1) children were hospitalised with RSV-associated ALRI in 2020 and 1·8 million (1·2–2·9) in 2021, which was substantially lower than the estimates for 2019 in this analysis with fewer studies (3·2 million [1·8–6·3]) and in our previous analysis (3·6 million [2·9–4·6]; appendix pp 19–20).^2^ Similar trends to the main analysis were observed when restricting the analysis to children hospitalised with RSV-associated ALRI requiring mechanical ventilation or ICU admission, across all age groups (appendix pp 21–22). Sensitivity analyses that excluded studies with a high risk of bias or without year-round testing, and those that reclassified studies on Indigenous populations, showed findings that were consistent with the main analysis (appendix pp 23–31). These findings are substantiated by results from the analysis of the 12-month moving rate of RSV-associated ALRI hospitalisations (figure 2, appendix p 40).

In 2020, the median of population mobility decreased by 13·8% (IQR 13·7–20·7) across high-income countries, 16·1% (16·1–23·6) across upper-middle-income countries, and 14·4% (14·4–14·4) across lower-middle-income countries (ie, Kenya; table 1). In 2021 and in the latest available period, population mobility had increased across all three regions compared with 2020. By March, 2022, mobility had not returned to the pre-pandemic level in high-income countries, but it had increased to above the pre-pandemic level in Kenya (table 1). The exploratory cross-correlation analysis suggested a significant correlation between the 12-month hospitalisation rate in children and population mobility metrics in 11 studies from eight countries, with eight studies from seven countries showing correlation coefficients above 0·5 (appendix pp 41–42). The optimal time lag was 0–2 months in high-income countries and 0–4 months in upper-middle-income countries (appendix p 32).

**Figure 3:**
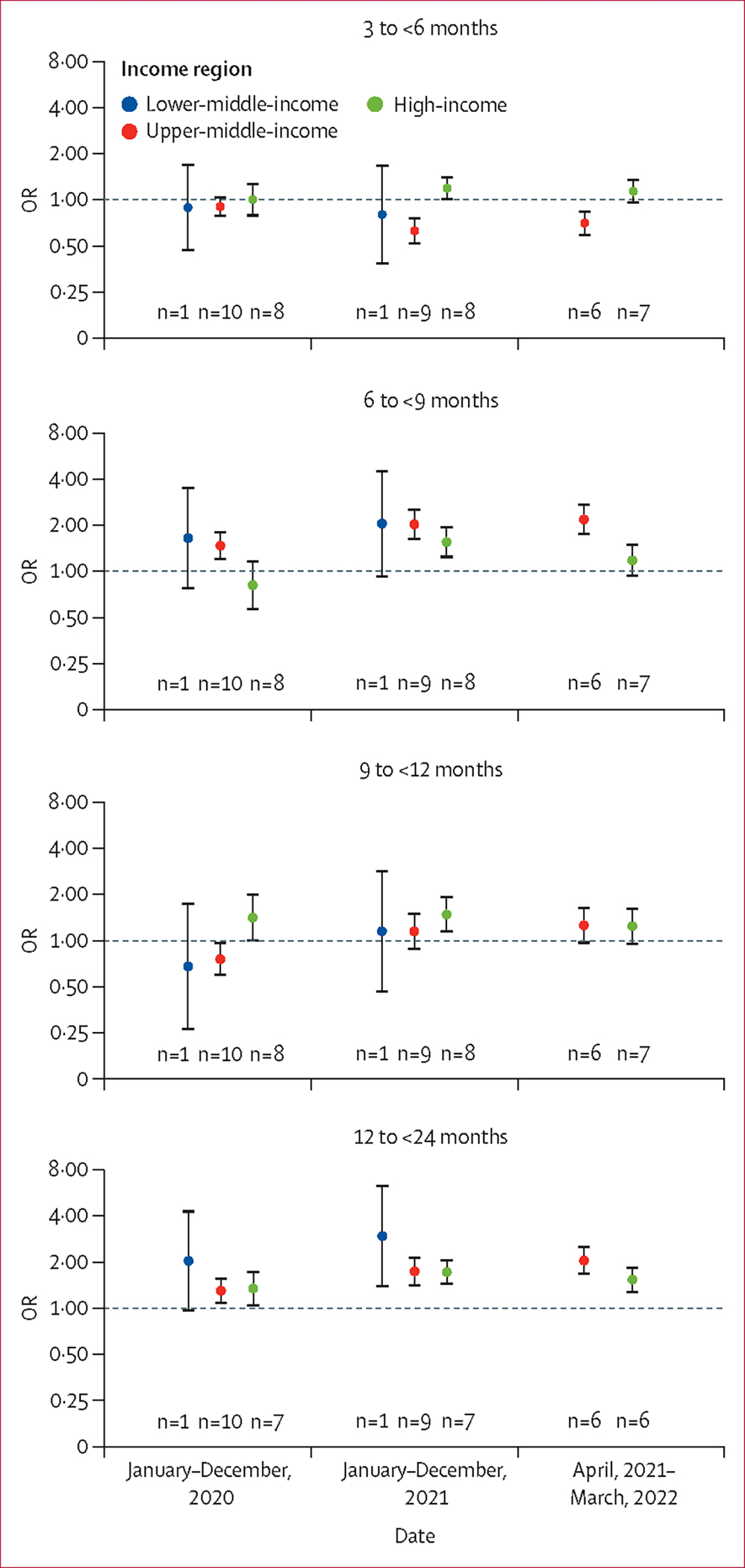
Relative risk of RSV-associated ALRI hospitalisation during the pandemic period by age group and World Bank income region Older age groups are compared with the youngest reference age group (ie, 0 to <3 months); the pandemic period is compared with the year 2019. Dots indicate point estimates and error bars indicate corresponding 95% uncertainty intervals. Each number indicates the number of studies contributing to each group. Underlying data are available in the appendix (pp 33–35). OR=odds ratio. RSV=respiratory syncytial virus. ALRI=acute lower respiratory infection.

Across all time periods and income regions, the rate of RSV-associated ALRI hospitalisations peaked in the youngest age group of children (aged 0 to <3 months) and decreased substantially with increasing age (table 1, appendix p 18). Compared with the youngest cohort of children (aged 0 to <3 months), a significantly higher proportion of children aged 12 to <24 months were hospitalised with RSV-associated ALRI during all time periods of the pandemic (ie, the year 2020, 2021, and the latest available period) than in the pre-pandemic year (ie, 2019) for both high-income countries and upper-middle-income countries, with corresponding ORs ranging from 1·30 (95% UI 1·07–1·59) in 2020 to 2·05 (1·66–2·54) in the latest available period (figure 3). Results from the subgroup analysis showed that both children aged 12 to <18 months and those aged 18 to <24 months accounted for a significantly increased proportion of RSV-associated ALRI hospitalised cases in high-income and upper-middle-income countries, except for those aged 12 to <18 months in 2020 (appendix p 45). Additionally, children aged 6 to <9 months accounted for a significantly higher proportion of hospitalised cases during all time periods of the pandemic in upper-middle-income countries than did infants younger than 3 months (figure 3). The increased proportion of children hospitalised with RSV-associated ALRI among older children (aged 12 to <24 months) was also observed in all sensitivity analyses (appendix pp 46–51). Similar patterns were observed, albeit with wider UIs (some were not significant), when restricting the analyses to children hospitalised with RSV-associated ALRI requiring supplemental oxygen or requiring mechanical ventilation or ICU admission (appendix p 52).

**Figure 4:**
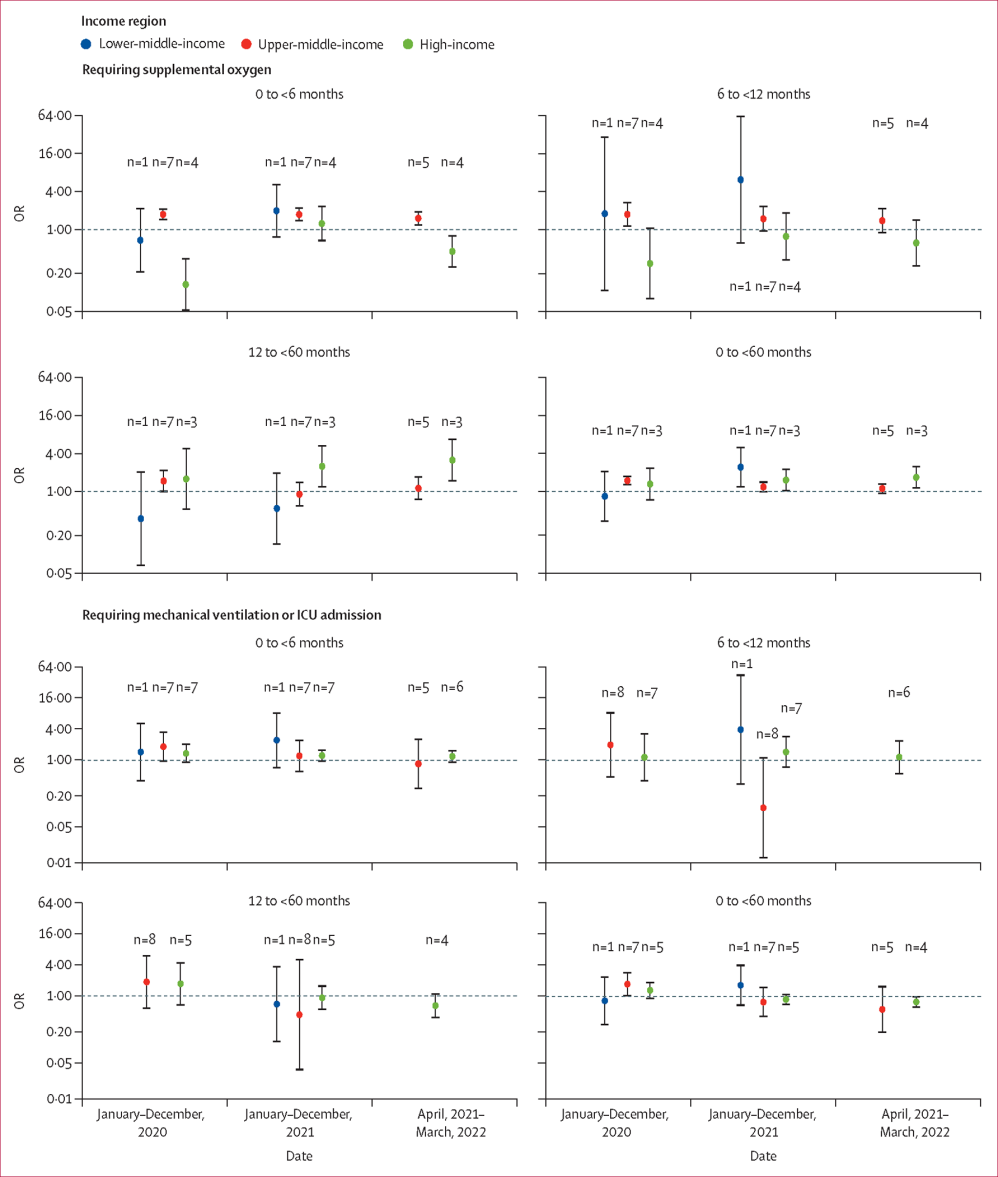
Relative risk of severe outcomes from RSV-associated ALRI hospitalisation during the pandemic period by age group and World Bank income region The pandemic period is compared with the year 2019. Dots indicate point estimates and error bars indicate corresponding 95% uncertainty intervals. Each number indicates the number of studies contributing to each group. Underlying data are available in the appendix (pp 36–37). OR=odds ratio. RSV=respiratory syncytial virus. ALRI=acute lower respiratory infection. ICU=intensive care unit.

We did not observe consistent patterns regarding changes in the proportions of severe outcomes during the pandemic years versus 2019, although significant findings were noted for certain combinations of age groups, time periods, and severe outcomes (figure 4, appendix p 53). Similar results were found in the sensitivity analysis that only included high-quality studies (appendix pp 55–56).

The in-hospital CFR of RSV-associated ALRI hospitalised cases seemed to be lower among children during the pandemic period than in 2019 for both high-income and upper-middle-income regions, despite wide UIs due to sparse data on in-hospital deaths (table 2). For the lower-middle-income region (ie, Kenya), an increased in-hospital CFR was observed in children during January, 2020–May, 2022, compared with 2019, but the UIs overlapped. Similar results were found for high-income countries in the sensitivity analysis that only included high-quality studies (appendix pp 26–28).

## Discussion

In this systemic analysis, we found that the rate of RSV-associated ALRI hospitalisations decreased consistently across different income regions in 2020, but had returned to the pre-pandemic level by March, 2022, only in high-income countries. Consistent with the pre-pandemic period, the rate of RSV-associated ALRI hospitalisations was highest in the youngest age group of children (aged 0 to <3 months) and decreased substantially with increasing age during the pandemic period. In both high-income and upper-middle-income regions, however, a significantly higher proportion of children aged 12–24 months were hospitalised with RSV-associated ALRI during the pandemic period than during the pre-pandemic period. No consistent changes in disease severity were observed.

We used 2019 as the pre-pandemic reference, assuming that RSV epidemiology would have remained stable if the COVID-19 pandemic had not occurred. This assumption was supported by our previous analysis of RSV-associated ALRI hospitalisations across 58 countries, in which the annual hospitalisation rate in children younger than 5 years fluctuated from 0·8 times to 1·2 times the country’s median yearly rate for most years, with no consistent trend over time.^28^ The assumption was also supported by the broadly consistent estimates for the year 2019 from this analysis and our previous analysis, which pooled all data for 2019 and earlier.^2^

The low rates of children hospitalised with RSV-associated ALRI in 2020 observed in this study are consistent with the widespread implementation of NPIs and the substantial decrease in population mobility observed that year. However, we noted a delayed increase in RSV-associated ALRI hospitalisation rates in middle-income countries in 2021, despite similar or higher population mobility than the pre-pandemic period. One possible explanation for this inconsistency is that healthcare systems in middle-income countries were less resilient to the burden of patients with COVID-19 than were those in high-income countries.^16,29^ Access to healthcare services might have also been affected in middle-income countries during this period.^30^ Furthermore, parental concerns over the risk of their infants acquiring COVID-19 at clinics or hospitals in middle-income countries, which remained overwhelmed for longer than did those in high-income countries, might have influenced health care-seeking behaviour. We have previously highlighted the striking gap of morbidity and mortality associated with RSV in communities and hospitals across LMICs.^2^ Taken together with the findings from this study, we speculate that this gap could be even more substantial than that in the pre-pandemic period, although active community-based studies are warranted to confirm this speculation.

During the pandemic years, children younger than 6 months (primarily aged 0 to <3 months) continued to have the highest hospitalisation burden from RSV; therefore, RSV-passive immunisation programmes targeting protection during the first 6 months of life should remain impactful. Despite the overall consistency in age patterns of hospitalisation rates between pre-pandemic and pandemic periods, we observed significantly higher proportions of older children (aged 12 to <24 months) hospitalised with RSV-related ALRI during the pandemic period, particularly after 2021. This increased proportion of older children was probably a result of the decreased exposure to RSV of infants and their mothers (during pregnancy) in the early phase of the pandemic;^8,31–33^ the RSV-naive birth cohort in 2020 remained susceptible until RSV circulation resurged in 2021. A mathematical modelling study published in 2022 showed a similar shift in age distribution in children hospitalised with RSV.^34^ Additionally, in this study, we observed a small but significantly increased proportion of children aged 12–24 months hospitalised with RSV-associated ALRI in the year 2020 in both high-income and upper-middle-income regions, which might be a result of changes in health care-seeking behaviour, criteria for admission, or parental precautions over protecting young infants. The increased proportion of older children hospitalised with RSV-associated ALRI during the pandemic observed in this study supports the dominant role of immunity (over age) in establishing the risk of severe RSV; RSV infections could result in the hospitalisation of older children with no previous exposure to RSV. Studies on the seroprevalence of RSV in children aged 12–24 months during the pandemic could help to test this hypothesis.

We observed large regional variations in the proportion of severe outcomes among children hospitalised with RSV-associated ALRI, even after accounting for the confounder of age (appendix p 53). As a result, our pooled global estimates might provide incomplete information when clear regional differences were observed in this analysis. Several factors could explain these large variations. First, health care-seeking behaviour and admission criteria could have altered during the pandemic period; parents might have chosen to seek health care only when their children were severely ill.^35–37^ Second, data show that coverage of the pneumococcal conjugate vaccine decreased during the pandemic,^38^ which could have resulted in an increased number of children hospitalised due to pneumococcal diseases who were tested positive for RSV. Third, the prevalence of common risk factors for severe outcomes, such as premature birth and comorbidities, could have changed^39,40^ and, therefore, influenced the proportion of severe outcomes reported among children hospitalised with RSV-associated ALRI. Fourth, the overall increase in susceptibility due to reduced exposure to RSV in the early phase of the pandemic might be associated with increased disease severity. A 2023 cohort study in Denmark found that older children (aged between 3 months and 17 years) without known risk factors for severe RSV disease had atypical complications that led to intubation.^8^ Finally, we could not account for the interaction between RSV and other respiratory viruses in this study, which might be associated with clinical severity.^41,42^

Our study has several limitations. First, as a global systematic analysis, we acknowledge common sources of heterogeneity in data, such as study setting, case definition, health care-seeking behaviour, RSV testing criteria and diagnostic assays, and criteria for admission. In addition, we acknowledge new sources of heterogeneity specific to our analysis. Study sites worldwide showed different trajectories of COVID-19 onset, surges, and response, which could have influenced RSV seasonality. This asynchrony could have complicated the interpretation of the meta-estimates. We tried to reconcile these heterogeneities by focusing only on the annualised hospitalisation burden of RSV (rather than a month-by-month estimate), which could help to filter out the atypical timing of RSV epidemics. Second, testing practices would have varied substantially during the pandemic period, and the impact of the COVID-19 pandemic on RSV testing practices varied in different income regions, although undertesting for RSV was adjusted as before.^2^ As testing for RSV was probably not missing at random, the adjustment for undertesting could have potentially biased the hospitalisation rate estimates upwards or downwards. Third, we used Google community mobility and COVID-19 NPI stringency index data to help interpret the observed changes in hospitalisation rates during different time periods. However, both these data sources have shortcomings. The mobility metrics were based on Google service users who enabled their location history, so might not represent infant and child populations, and the single mobility metric of visits to retail and recreation places might not represent population mobility well. The COVID-19 NPI stringency index, as a composite metric, might not capture well the substantial variations in the content of various NPIs across different countries. Other potential important factors, such as school breaks,^7^ were not considered in this analysis. Fourth, we did not extract data on sex and could not assess the potential differences in RSV epidemiology by sex. Finally, due to the time lag between data collection, analysis, and reporting, we could report data only up to March, 2022, missing the latest period at the time of writing.

During the pandemic, resources were reallocated to the prevention and treatment of COVID-19, especially for the adult population. As a result, data on the disease burden of RSV in young children during this period are relatively sparse compared with our previously published estimates of global RSV disease burden for the year 2019,^2^ and most of these data are from high-income countries (mainly in the European region). This sparsity resulted in relatively wide UIs, particularly for the CFR and OR estimates in the middle-income region. Non-significant estimates should not be interpreted as the absence of significance. Low-income and lower-middle-income countries that have a disproportionately high RSV burden were largely under-represented. According to RSV GEN members based in low-income and lower-middle-income countries, collection of RSV data of sufficient quality was substantially interrupted by the COVID-19 pandemic. In this study, only one country (Kenya) was from the lower-middle-income region and no countries were included from the low-income region; therefore, the pooled global estimates are biased compared with our previous estimates.^2^ More studies and high-quality surveillance data from low-income and lower-middle-income countries are needed to understand RSV epidemiology in the era following the pandemic. Furthermore, the pandemic disrupted community-based studies, which are essential for understanding the underlying disease burden of RSV in populations that could not receive quality health care in LMICs during this period.

In summary, this study provides a global overview of the changes in the hospitalisation burden of RSV-associated ALRI in young children during the COVID-19 pandemic. Although hospitalisation rates have returned to pre-pandemic rates in high-income countries, the consistently lower rates during the pandemic period than in the pre-pandemic period in middle-income countries might be a result of the negative impact of the COVID-19 pandemic on health-care systems and health-care accessibility. Despite the observed increase in the proportion of older children hospitalised with RSV-associated ALRI likely due to reduced exposure to RSV during the early phase of the pandemic, further surveillance is needed to monitor whether patterns in age distribution will return to those in the pre-pandemic period once the cohort of children born during the pandemic have all had their first RSV infection. Surveillance is also required to monitor the possible effects of RSV prevention products on these changes in age distribution. RSV surveillance needs to be established (or re-established) to monitor changes in RSV epidemiology, especially in low-income and lower-middle-income countries.

## Supplementary Material

AppendixMaterial

## Figures and Tables

**Table 1:** Estimates of hospitalisation burden for children with RSV-associated ALRI before and during the pandemic period by World Bank income region

	January-December, 2019 (from this study)	January-December, 2019 (from Li et al, 2022)^2^	January-December, 2020	January-December, 2021*	April, 2021-March, 2022

**High-income region**					
Median NPI stringency index (IQR)†	0	0	42⋅0 (39⋅8 to 47⋅7)	43⋅8 (42⋅7 to 46⋅7)	41⋅5 (37⋅2 to 46⋅6)
Median changes in mobility, % (IQR)	0	0	−13⋅8 (−20⋅7 to −13⋅7)	−6⋅0 (−12⋅3 to −1⋅8)	−5⋅0 (−9⋅3 to 0⋅5)
Age 0 to <3 months					
Number of studies	6	19	6	6	5
Hospital admission rate (95% UI)	33⋅5 (22⋅7 to 49⋅4)	34⋅7 (21⋅5 to 56⋅2)	8⋅3 (2⋅0 to 34⋅7)	26⋅9 (14⋅8 to 48⋅9)	36⋅2 (23⋅3 to 56⋅2)
Number of episodes (95% UI)	105 (71 to 155)	116 (72 to 188)	25 (6 to 107)	82 (45 to 148)	110 (71 to 171)
Age 3 to <6 months					
Number of studies	6	21	6	6	5
Hospital admission rate (95% UI)	19⋅0 (14⋅4to 25⋅0)	20⋅7 (13⋅5 to 31⋅6)	4⋅8 (1⋅6 to 14⋅3)	14⋅8 (9⋅3 to 23⋅6)	18⋅3 (14⋅4 to 23⋅3)
Number of episodes (95% UI)	60 (45 to 79)	69 (45 to 106)	15 (5 to 44)	45 (28 to 72)	56 (44 to 71)
Age 0 to <6 months‡					
Number of studies	6	27	7	6	5
Hospital admission rate (95% UI)	26⋅4 (19⋅3 to 36⋅0)	28⋅4 (20⋅2 to 40⋅0)	5⋅7 (1⋅6 to 20⋅4)	20⋅9 (11⋅9 to 36⋅5)	27⋅8 (19⋅2 to 40⋅1)
Number of episodes (95% UI)	165 (121 to 226)	190 (135 to 267)	35 (10 to 125)	127 (72 to 222)	169 (117 to 243)
Age 6 to <12 months					
Number of studies	6	27	7	6	5
Hospital admission rate (95% UI)	7⋅2 (4⋅2 to 12⋅3)	11⋅2 (7⋅5 to 16⋅7)	1⋅5 (0⋅7 to 3⋅2)	6⋅6 (4⋅0 to 10⋅7)	7⋅7 (6⋅0 to 9⋅9)
Number of episodes (95% UI)	45 (27 to 77)	75 (50 to 112)	9 (4 to 20)	40 (24 to 65)	47 (36 to 60)
Age 0 to <12 months‡					
Number of studies	6	41	8	6	5
Hospital admission rate (95% UI)	17⋅7 (13⋅7 to 22⋅8)	22⋅0 (17⋅1 to 28⋅4)	3⋅9 (1⋅3 to 11⋅8)	14⋅1 (8⋅5 to 23⋅3)	18⋅2 (14⋅3 to 23⋅1)
Number of episodes (95% UI)	222 (172 to 287)	294 (228 to 380)	48 (16 to 145)	171 (104 to 283)	221 (174 to 281)
Age 12 to <60 months					
Number of studies	6	17	7	6	5
Hospital admission rate (95% UI)	1⋅7 (1⋅0to 2⋅8)	1⋅6 (1⋅2 to 2⋅1)	0⋅4 (0⋅1 to 1⋅5)	1⋅7 (0⋅8 to 3⋅5)	2⋅3 (1⋅3 to 4⋅0)
Number of episodes (95% UI)	88 (51 to 151)	88 (67 to 116)	22 (6 to 79)	85 (41 to 178)	115 (65 to 204)
Age 0 to <60 months‡					
Number of studies	6	51	7	6	5
Hospital admission rate (95% UI)	5⋅0 (3⋅6 to 6⋅8)	6⋅0 (4⋅7 to 7⋅7)	1⋅0 (0⋅2 to 4⋅3)	4⋅5 (2⋅6 to 7⋅7)	6⋅0 (5⋅4 to 6⋅8)
Number of episodes (95% UI)	325 (235 to 448)	409 (319 to 524)	66 (16 to 279)	282 (163 to 490)	381 (339 to 429)
**Upper-middle-income region**					
Median NPI stringency index (IQR)†	0	0	55⋅6 (54⋅1 to 55⋅6)	56 6 (52⋅6 to 56 6)	50⋅4 (49⋅1 to 50⋅4)
Median changes in mobility, % (IQR)	0	0	−16⋅1 (−23⋅6 to −16⋅1)	−9⋅4 (−9⋅4 to −4⋅6)	−7⋅8 (−7⋅8 to 3⋅4)
Age 0 to <3 months					
Number of studies	3	16	5	5	5
Hospital admission rate (95% UI)	47⋅6 (13⋅8 to 163⋅9)	26⋅4 (12⋅8 to 54⋅5)	26⋅2 (9⋅7 to 70⋅9)	12⋅2 (9⋅0 to 16⋅6)	20⋅5 (6⋅3 to 67⋅4)
Number of episodes (95% UI)	372 (108 to 1281)	236 (114 to 486)	192 (71 to 520)	83 (62 to 113)	140 (43 to 459)
Age 3 to <6 months					
Number of studies	3	16	5	5	5
Hospital admission rate (95% UI)	31⋅1 (7⋅7 to 125⋅1)	20⋅6 (11⋅8 to 36⋅0)	19⋅1 (4⋅0 to 92⋅1)	7⋅1 (4⋅7 to 10⋅8)	11⋅1 (2⋅8 to 43⋅6)
Number of episodes (95% UI)	243 (60 to 978)	184 (106 to 321)	140 (29 to 676)	48 (32 to 73)	76 (19 to 297)
Age 0 to <6 months‡					
Number of studies	3	16	5	5	5
Hospital admission rate (95% UI)	40⋅5 (10⋅8 to 152⋅0)	24⋅3 (13⋅2 to 44⋅7)	24⋅4 (7⋅6 to 78⋅5)	9⋅9 (7⋅8 to 12⋅7)	15⋅9 (4⋅7 to 53⋅7)
Number of episodes (95% UI)	633 (169 to 2376)	434 (236 to 798)	359 (112 to 1152)	135 (106 to 173)	216 (64 to 732)
Age 6 to <12 months					
Number of studies	3	15	5	5	5
Hospital admission rate (95% UI)	15⋅2 (3⋅4 to 67⋅3)	12⋅1 (6⋅6 to 22⋅1)	10⋅0 (1⋅5 to 67⋅8)	4⋅5 (1⋅5 to 13⋅5)	7⋅2 (1⋅8 to 28⋅8)
Number of episodes (95% UI)	238 (54 to 1052)	215 (117 to 394)	147 (22 to 995)	61 (20 to 184)	98 (24 to 393)
Age 0 to <12 months‡					
Number of studies	3	15	5	5	5
Hospital admission rate (95% UI)	27⋅6 (7⋅3 to 104⋅2)	18⋅7 (10⋅2 to 34⋅5)	18⋅2 (4⋅6 to 72⋅7)	8⋅1 (4⋅2 to 15⋅4)	11⋅7 (3⋅3 to 40⋅8)
Number of episodes (95% UI)	864 (229 to 3260)	669 (363 to 1232)	535 (134 to 2133)	219 (115 to 420)	318 (91 to 1112)
Age 12 to <60 months					
Number of studies	2	8	4	4	4
Hospital admission rate (95% UI)	0⋅9 (0⋅3 to 3⋅3)	1⋅5 (0⋅8 to 2⋅8)	1⋅1 (0⋅2 to 7⋅0)	0⋅8 (0⋅4 to 1⋅6)	0⋅8 (0⋅3 to 2⋅7)
Number of episodes (95% UI)	127 (36 to 450)	220 (117 to 415)	151 (24 to 947)	102 (50 to 208)	109 (35 to 343)
Age 0 to <60 months‡					
Number of studies	2	16	4	4	4
Hospital admission rate (95% UI)	3⋅4 (1⋅2 to 9⋅7)	6⋅2 (3⋅8 to 10⋅3)	3⋅1 (0⋅7 to 14⋅3)	1⋅9 (1⋅3 to 2⋅7)	2⋅1 (0⋅7 to 6⋅1)
Number of episodes (95% UI)	581 (206 to 1643)	1139 (693 to 1872)	501 (107 to 2340)	297 (210 to 419)	334 (117 to 956)
**Lower-middle-income region**					
Median NPI stringency index (IQR)†	0	0	57⋅8 (57⋅8 to 57⋅8)	56⋅1 (56⋅1 to 56⋅1)	52⋅8 (52⋅8 to 52⋅8)
Median changes in mobility, % (IQR)	0	0	−14⋅4 (−14⋅4 to −14⋅4)	19⋅9 (19⋅9 to 19⋅9)	32⋅3 (32⋅3 to 32⋅3)
Age 0 to <3 months					
Number of studies	1	11	1	1	0
Hospital admission rate (95% UI)	25⋅5 (20⋅0 to 32⋅6)	31⋅0 (17⋅0 to 56⋅4)	14⋅6 (10⋅4 to 20⋅6)	10⋅4 (7⋅0 to 15⋅4)	..
Number of episodes (95% UI)	436 (341 to 557)	485 (267 to 884)	248 (177 to 349)	176 (119 to 260)	..
Age 3 to <6 months					
Number of studies	1	13	1	1	0
Hospital admission rate (95% UI)	21⋅9 (16⋅5 to 29⋅1)	19⋅2 (11⋅5 to 32⋅1)	13⋅6 (8⋅9 to 20⋅6)	7⋅8 (4⋅7 to 13⋅0)	..
Number of episodes (95% UI)	374 (282 to 497)	301 (180 to 503)	230 (152 to 350)	133 (80 to 220)	..
Age 0 to <6 months‡					
Number of studies	1	12	1	1	0
Hospital admission rate (95% UI)	24⋅1 (20⋅0 to 28⋅9)	27⋅9 (16⋅7 to 46⋅6)	13⋅9 (10⋅7 to 18⋅2)	9⋅7 (7⋅2 to 13⋅1)	..
Number of episodes (95% UI)	822 (683 to 988)	873 (523 to 1460)	473 (362 to 618)	329 (243 to 445)	..
Age 6 to <12 months					
Number of studies	1	13	1	1	0
Hospital admission rate (95% UI)	9⋅1 (6⋅7 to 12⋅3)	12⋅1 (6⋅5 to 22⋅8)	5⋅9 (3⋅9 to 8⋅8)	5⋅9 (3⋅9to 8⋅9)	..
Number of episodes (95% UI)	311 (230 to 420)	381 (203 to 715)	200 (134 to 298)	201 (133 to 302)	..
Age 0 to <12 months‡					
Number of studies	1	20	1	1	0
Hospital admission rate (95% UI)	17⋅2 (14⋅7to 20⋅1)	17⋅5 (11⋅5 to 26⋅5)	9⋅8 (7⋅9 to 12⋅3)	8⋅2 (6⋅4 to 10⋅4)	..
Number of episodes (95% UI)	1173 (1005 to 1370)	1095 (722 to 1661)	666 (533 to 832)	553 (435 to 703)	..
Age 12 to <60 months					
Number of studies	1	12	1	1	0
Hospital admission rate (95% UI)	0⋅7 (0⋅5 to 1⋅0)	1⋅6 (1⋅0 to 2⋅7)	0⋅5 (0⋅4 to 0⋅8)	1⋅0 (0⋅7 to 1⋅4)	..
Number of episodes (95% UI)	188 (128 to 276)	396 (235 to 667)	147 (95 to 228)	269 (194 to 374)	..
Age 0 to <60 months‡					
Number of studies	1	22	1	1	0
Hospital admission rate (95% UI)	4⋅1 (3⋅5 to 4⋅7)	6⋅2 (4⋅0 to 9⋅4)	2⋅4 (1⋅9 to 2⋅8)	2⋅2 (1⋅8 to 2⋅7)	..
Number of episodes (95% UI)	1378 (1195 to 1588)	1908 (1251 to 2909)	795 (657 to 963)	755 (620 to 920)	..

Hospitalisation rate is the number of children (in thousands) per 1000 children. RSV=respiratory syncytial virus. ALRI=acute lower respiratory infection. NPI=non-pharmaceutical intervention. UI=uncertainty interval.

* Data for 2021 (ie, January-December) overlap with data for the latest available period (ie, April, 2021-March, 2022).

† The median (IQR) COVID-19 NPI stringency index and changes in mobility were calculated on the basis of the last month of the corresponding year by income region.

‡ Point estimates and UI estimates are not necessarily equal to the sum of the estimates by finer age bands because the studies that contributed to age-specific estimates were different.

**Table 2:** In-hospital CFR estimates for children with RSV-associated ALRI before and during the pandemic period by World Bank income region

	January-December, 2019 (from this study)	January-December, 2019 (from Li et al, 2022)^2^	January, 2020-May, 2022

**High-income region**			
Age 0 to <12 months			
Number of studies	8	29	11
In-hospital CFR, % (95% UI)	0⋅1% (<0⋅1 to 1⋅3)	0⋅1% (0⋅1 to 0⋅3)	<0⋅1% (<0⋅1 to 2⋅1)
Age 12 to <60 months			
Number of studies	7	17	8
In-hospital CFR, % (95% UI)	0⋅3% (<0⋅1 to 2⋅2)	0⋅2% (0⋅1 to 0⋅4)	0⋅1% (<0⋅1 to 5⋅3)
Age 0 to <60 months			
Number of studies	7	26	11
In-hospital CFR, % (95% UI)	0⋅1% (<0⋅1 to 0⋅5)	0⋅1% (0⋅1 to 0⋅2)	0⋅1% (<0⋅1 to 0⋅8)
**Upper-middle income region**			
Age 0 to <12 months			
Number of studies	10	27	10
In-hospital CFR, % (95% UI)	0⋅4% (0⋅1 to 1⋅0)	0⋅8% (0⋅5 to 1⋅3)	<0⋅1% (<0⋅1 to 67)
Age 0 to <60 months			
Number of studies	10	30	11
In-hospital CFR, % (95% UI)	0⋅2% (0⋅1 to 0⋅7)	0⋅6% (0⋅3 to 1⋅0)	<0⋅1% (<0⋅1 to 10⋅0)
**Lower-middle-income region**			
Age 0 to <12 months			
Number of studies	1	22	1
In-hospital CFR, % (95% UI)	1⋅9% (0⋅5 to 7⋅4)	1⋅5% (0⋅7 to 3⋅2)	2⋅2% (0⋅6 to 8⋅4)
Age 0 to <60 months			
Number of studies	1	26	1
In-hospital CFR, % (95% UI)	17% (0⋅4 to 6⋅5)	0⋅8% (0⋅4 to 1⋅5)	17% (0⋅4 to 6⋅6)

CFR=case fatality ratio. RSV=respiratory syncytial virus. ALRI=acute lower respiratory infection. UI=uncertainty interval.

## Data Availability

Aggregated data on RSV epidemiology used for this study and additional technical model outputs are shared in GitHub. All other data included in the analysis are publicly available and have been properly cited.

## Research in context

### Evidence before this study

Respiratory syncytial virus (RSV) causes a substantial disease burden in children younger than 5 years globally. The COVID-19 pandemic has been reported to have affected RSV epidemiology and disease burden, which could have important implications for RSV prevention and control strategies. We searched PubMed, with no language restrictions, for studies reporting the effects of the COVID-19 pandemic on RSV epidemiology or disease burden published between Jan 1, 2020, and July 4, 2023, using the following search terms: (“COVID-19” OR “SARS-CoV-2”) AND (“respiratory syncytial virus” OR “RSV”) AND (“hospitalisation” OR “hospitalization” OR “mortality” OR “incidence” OR “epidemiology” OR “burden”). Both primary studies from multiple countries (eg, France, Denmark, England, Australia, and South Africa) and narrative systematic reviews noted changes in RSV epidemiology during the COVID-19 pandemic in terms of hospitalisation rate, age distribution, and disease severity, although these changes were not consistent across studies, partly due to reporting variations in the age groups and time periods investigated.

### Added value of this study

We supplemented the published literature with granular unpublished data, which provided an increased resolution in age groups and time periods to help understand the temporal changes in the incidence, age distribution, and disease severity of children hospitalised with RSV-associated acute lower respiratory infection (ALRI) from a global perspective. Although hospitalisation rates decreased consistently across different income regions in 2020, they started to rise in 2021 and showed different trajectories by income region. The high-income region reported similar hospitalisation rates by March, 2022, to those in 2019, whereas the middle-income region reported lower rates than those in 2019. Consistent with the pre-pandemic period, hospitalisation rates were highest in younger infants (aged 0 to <3 months) and decreased with increasing age; however, we also noted an increased proportion of children hospitalised with RSV-associated ALRI in the second year of life (aged 12 to <24 months) during the pandemic period in the high-income and upper-middle-income regions. This observation is possibly due to decreased immunity from natural infection of the infant population during the first year of the COVID-19 pandemic (2020), when exposure to RSV was lower than that in the pre-pandemic period. We did not observe consistent changes overall in disease severity, which was measured by the proportion of children hospitalised with RSV-associated ALRI requiring oxygen supplementation or requiring mechanical ventilation or admission to intensive care, as well as by the in-hospital case fatality ratio.

### Implications of all the available evidence

Our findings report the disruptions in RSV epidemiology observed during the COVID-19 pandemic, which had still not fully returned to pre-pandemic patterns by March, 2022. The varied changes in the hospitalisation burden of RSV by income region and the changes in the age distribution of children hospitalised with RSV-associated ALRI highlight the importance of establishing (or re-establishing) RSV surveillance, particularly in low-income and lower-middle-income countries.

## RSV GEN investigators

Bingbing Cong, You Li (Nanjing Medical University, Nanjing, China); Harish Nair, Uğurcan Koç (University of Edinburgh, Edinburgh, UK); Teresa Bandeira, Zakhar Shchomak, Rosário Barreto (Hospital Santa Maria, Centro Hospitalar Universitário Lisboa Norte, Lisbon, Portugal); Quique Bassat, David Torres-Fernandez (ISGlobal, Hospital Clínic–Universitat de Barcelona, Barcelona, Spain); Neydis Baute (Mapulaneng Hospital, Mpumalanga, South Africa); Louis Bont (University Medical Center Utrecht, Utrecht, Netherlands); Sara S Bressler, Dana Bruden (Centers for Disease Control and Prevention, Anchorage, AK, USA); Jean-Sebastien Casalegno (Hôpital de la Croix-Rousse, Lyon, France); Come Horvat, Dominique Ploin (Hôpital Femme Mère Enfant, Lyon, France); Giorgi Chakhunashvili, Irakli Karseladze (National Center for Disease Control and Public Health, Tbilisi, Georgia); Cheryl Cohen, Jocelyn Moyes, Nicole Wolter, Sibongile Walaza, Anne von Gottberg, Mignon du Plessis (National Institute for Communicable Diseases of the National Health Laboratory Service, Johannesburg, South Africa); Halima Dawood, Fathima Naby (Greys Hospital, Pietermaritzburg, South Africa); Christine Desnoyers (Yukon-Kuskokwim Health Corporation, Bethel, AK, USA); Laura Hammitt, Rachel Hartman, Marqia Sandoval (Center for Indigenous Health, Department of International Health, Johns Hopkins Bloomberg School of Public Health, Baltimore, MD, USA); Terho Heikkinen (University of Turku and Turku University Hospital, Turku, Finland); Q Sue Huang (Institute of Environmental Science and Research, Wellington, New Zealand); Joško Markić, Dina Mrčela, Petra Milić, Daniela Veljačić Visković (University Hospital Split, Split, Croatia); Ainara Mira-Iglesias, Alejandro Orrico-Sánchez, Javier Díez-Domingo, Arantxa Urchueguía (Fundación para el Fomento de la Investigación Sanitaria y Biomédica de la Comunitat Valenciana, Salud Pública, Valencia, Spain); Omphile Mekgoe (Klerksdorp Hospital, Klerksdorp, South Africa); D James Nokes, Nickson Murunga, Martin Mutunga (Kenya Medical Research Institute–WellcomeTrust Research Programme, Kilifi, Kenya); Gary Reubenson (Faculty of Health Sciences, University of the Witwatersrand, Johannesburg, South Africa); Euri Seo (Dongguk University, Goyang, South Korea); Rosalyn Singleton, Jennifer Dobson, James W Keck (Alaska Native Tribal Health Consortium, Anchorage, AK, USA); Chee Fu Yung (KK Women’s and Children’s Hospital, Singapore); Heather J Zar (University of Cape Town and Red Cross War Memorial Children’s Hospital, Cape Town, South Africa).

## PROMISE investigators

Jeroen Aerssens, Gabriela Ispas (Janssen, Beerse, Belgium); Bahar Ahani (AstraZeneca, Gaithersburg, MD, USA); Jessica Atwell, Elizabeth Begier, Tin Tin Htar (Pfizer, Paris, France); Mathieu Bangert, Rolf Kramer, Charlotte Vernhes (Sanofi Pasteur, Lyon, France); Philippe Beutels (University of Antwerp, Antwerpen, Belgium); Louis Bont (University Medical Centre Utrecht, Utrecht, Netherlands); Harry Campbell, Harish Nair, You Li, Richard Osei-Yeboah, Sebastien Kenmoe, Xin Wang (University of Edinburgh, Edinburgh, UK); Arantxa Urchueguía-Fornes, Ainara Mira-Iglesias, Alejandro Orrico-Sánchez, Javier Díez-Domingo (Instituto de Salud Carlos III, Valencia, Spain); Rachel Cohen, Gael Dos Santos, Theo Last (GlaxoSmithKline, Wavre, Belgium); Veena Kumar (Novavax, Gaithersburg, MD, USA); Nuria Machin (Teamit Research, Barcelona, Spain); Hanna Nohynek (Finnish National Institute for Health and Welfare, Helsinki, Finland); Peter Openshaw (Imperial College London, London, UK); John Paget (Netherlands Institute for Health Services Research, Utrecht, Netherlands); Andrew Pollard (University of Oxford, Oxford, UK); Anne Teirlinck (National Institute for Public Health and the Environment, Bilthoven, Netherlands).
