# Supplementary material for: Changes in the global hospitalisation burden of respiratory syncytial virus in young children during the COVID-19 pandemic: a systematic analysis
Source: Lancet Infect Dis. Author manuscript; Available in PMC 2024 Jul 31. (PMC11290460; doi:10.1016/S1473-3099(23)00630-8)
Supplement: AppendixMaterial [file NIHMS2007767-supplement-AppendixMaterial.pdf]

THE LANCET
Infectious Diseases

Supplementary appendix

This appendix formed part of the original submission and has been peer reviewed. We post it as supplied by the authors.

Supplement to: Cong B, Koç U, Bandeira T, et al. Changes in the global hospitalisation burden of respiratory syncytial virus in young children during the COVID-19 pandemic: a systematic analysis. *Lancet Infect Dis* 2023; published online Dec 20. https://doi.org/10.1016/S1473-3099(23)00630-8.

Supplementary appendix

Understanding the changes in global hospitalisation burden of respiratory syncytial virus in young children during the COVID-19 pandemic: a systematic analysis up to March 2022

Contents

Detailed description of data included in the analysis ... 4

Text S1. Search Strategy ... 4

Text S2. Quality scoring criteria for included studies. ... 8

Table S1. Summary of unpublished data from Respiratory Virus Global Epidemiology Network (i.e., previously RSV GEN) investigators ... 9

Table S2. Methodological details about generalized linear mixed-effects model used for analysing various outcomes. ... 10

Table S3. Summary of studies that contributed to RSV-associated ALRI hospital admission rate estimates.. 11

Table S4. Summary of studies that contributed to RSV in-hospital case fatality ratio or proportion of requiring supplemental oxygen or ICU admission estimates. ... 13

Supplementary tables of results ... 18

Table S5. Estimates of RSV-associated ALRI hospitalisation burden (hospitalisation rate per 1000 children and number in thousands) in children aged 12–<24 months and 24-<60 months by World Bank income region in different time periods ... 18

Table S6. Estimates of RSV-associated ALRI hospitalisation burden (hospitalisation rate per 1000 children and number in thousands) in children younger than 5 years by Country Development Status in different time periods ... 19

Table S7. Estimates of RSV-associated ALRI hospitalisation burden that requiring mechanical ventilation and ICU admission (hospitalisation rate per 1000 children and number in thousands) in children younger than 5 years by World Bank income region in different time periods. ... 21

Table S8. Results of sensitivity analysis that only included studies with quality scores ≥0.6. ... 23

Table S9. Results of sensitivity analysis that excluded studies with non-year-round testing ... 26

Table S10. Results of sensitivity analysis that reclassified studies focusing on indigenous populations as high-income/industrialised countries ... 29

Table S11. Median, IQR and range of optimal lag time between 12-month moving average RSV-associated ALRI hospitalisation rates and Retail & recreation index by World Bank income region. ... 32

Table S12. Odds ratio for observing RSV-associated ALRI hospitalisations in older age groups (compared with 0–<3 months) during the COVID-19 pandemic period (compared to the year 2019) ... 33

Table S13. Comparison of proportion of RSV-associated ALRI hospitalisations that needed supplemental oxygen and required mechanical ventilation or ICU admission between pre-pandemic and pandemic period, by broader age group and World Bank Income Region. ... 36

Supplementary figures ... 38

Figure S1. Geographical distribution of included studies reporting RSV-associated ALRI hospitalisation rates. ... 38

Figure S2. Geographical distribution of included studies reporting proportion of severe outcomes or in-hospital CFR among hospitalised RSV-ALRI cases. ... 39

Figure S3. Change in the 12-month retrospective hospitalisation rates of RSV-associated ALRI by broader age group and World Bank income region. ... 40

Figure S4. Maximal correlation coefficient of 12-month moving average RSV-associated ALRI hospitalisation rates and Retail & recreation index by age group and World Bank income region. ... 41

Figure S5. Changes over time of 12-month moving average RSV-associated ALRI hospitalisation rate in infants less than 6 months and Retail & recreation index by study site. ... 42

Figure S6. Three-month moving average of monthly RSV-associated ALRI hospitalisations and Retail & recreation index for children young than five years, stratified by age group and study site. ... 44

Figure S7. Odds ratio for observing RSV-associated ALRI hospitalisations in children aged 12–<18 months and 18–<24 months (compared with 0–<3 months) during the COVID-19 pandemic period (compared to the year 2019). ... 45

Figure S8. Odds ratio for observing RSV-associated ALRI hospitalisations in older age groups (compared with 0–<3 months) during the COVID-19 pandemic period (compared to the year 2019), when only including studies with quality scores ≥0.6. 47

Figure S9. Odds ratio for observing RSV-associated ALRI hospitalisations in older age groups (compared with 0–<3 months) during the COVID-19 pandemic period (compared to the year 2019), when only including studies with year-round testing........ 49

Figure S10. Odds ratio for observing RSV-associated ALRI hospitalisations in older age groups (compared with 0–<3 months) during the COVID-19 pandemic period (compared to the year 2019), when reclassifying studies focusing on indigenous population........ 51

Figure S11. Odds ratio for observing RSV-associated ALRI hospitalisations in older age groups (compared with 0–<3 months) during the COVID-19 pandemic period (compared to the year 2019)........ 52

Figure S12. Comparison of proportion of RSV-associated ALRI hospitalisations that needed supplemental oxygen and required mechanical ventilation or ICU admission between pre-pandemic and pandemic period, by World Bank Income Region and finer age group........ 53

Figure S13. Comparison of proportion of RSV-associated ALRI hospitalisations requiring mechanical ventilation or ICU admission in children aged 0–<24 months between pre-pandemic and pandemic period, by World Bank Income Region........ 54

Figure S14. Comparison of proportion of RSV-associated ALRI hospitalisations that needed supplemental oxygen and required mechanical ventilation or ICU admission between pre-pandemic and pandemic period, by World Bank Income Region and age group, when only including studies with quality scores ≥0.6........ 56

GATHER checklist 57

PRISMA 2020 Checklist........ 58

REFERENCES 61

Detailed description of data included in the analysis

Text S1. Search Strategy

Medline

1. exp Respiratory Syncytial Viruses/ or exp Respiratory Syncytial Virus, Human/ or exp Respiratory Syncytial Virus Infections/ or RSV.mp.

2. respiratory syncytial virus*.mp.

3. pneumonia.mp. or exp Pneumonia/ or exp Pneumonia, Viral/

4. bronchiolitis.mp. or exp Bronchiolitis/ or exp Bronchiolitis, Viral/

5. exp Respiratory Tract Infections/ or respiratory infection*.mp.

6. exp Respiratory Tract Diseases/ or respiratory disease*.mp.

7. incidence.mp. or exp Incidence/

8. prevalence.mp. or exp Prevalence/

9. exp Child Mortality/ or exp Infant Mortality/ or mortality.mp. or exp Hospital Mortality/ or exp Mortality/

10. death*.mp. or exp Death/ or exp "Cause of Death"/

11. morbidity.mp. or exp Morbidity/

12. burden.mp.

13. epidemiology.mp. or exp Epidemiology/

14. 1 or 2

15. 3 or 4 or 5 or 6

16. 7 or 8 or 9 or 10 or 11 or 12 or 13

17. 15 or 16

18. 14 and 17

19. limit 18 to (yr="January 2020–June 2022" and ("all infant (birth to 23 months)" or "newborn infant (birth to 1 month)" or "infant (1 to 23 months)" or "preschool child (2 to 5 years)") and (female or humans or male))

EMBASE

1. RSV.mp. or exp Respiratory syncytial pneumovirus/ or exp respiratory syncytial virus infection/

2. respiratory syncytial virus*.mp.

3. exp community acquired pneumonia/ or exp pneumonia/ or exp virus pneumonia/ or exp infectious pneumonia/ or pneumonia.mp.

4. bronchiolitis.mp. or exp bronchiolitis/ or exp viral bronchiolitis/

5. exp respiratory tract infection/ or exp lower respiratory tract infection/ or respiratory infection*.mp.

6. exp respiratory tract disease/ or respiratory disease*.mp.

7. exp incidence/ or incidence.mp.

8. prevalence.mp. or exp prevalence/

9. exp newborn mortality/ or exp mortality/ or exp childhood mortality/ or mortality.mp. or exp infant mortality/

10. exp death/ or death*.mp. or exp "cause of death"/ or exp child death/

11. morbidity.mp. or exp morbidity/ or exp newborn morbidity/

12. burden.mp.

13. exp epidemiology/ or epidemiology.mp.

14. 1 or 2

15. 3 or 4 or 5 or 6

16. 7 or 8 or 9 or 10 or 11 or 12 or 13

17. 15 or 16

18. 14 and 17

19. limit 18 to (yr=" January 2020 –June 2022" and (infant or preschool child <1 to 6 years>))

Global Health

1. RSV.mp.

2. exp human respiratory syncytial virus/

3. respiratory syncytial virus*.mp.

4. exp community acquired pneumonia/ or pneumonia*.mp. or exp pneumonia/

5. bronchiolitis.mp. or exp bronchiolitis/

6. respiratory infection*.mp.

7. exp respiratory diseases/

8. respiratory disease*.mp.

9. incidence.mp. or exp disease incidence/ or exp incidence/

10. prevalence*.mp. or exp disease prevalence/

11. mortality.mp. or exp infant mortality/ or exp neonatal mortality/ or exp mortality/

12. death*.mp. or exp death/ or exp "causes of death"/

13. morbidity.mp. or exp morbidity/

14. exp epidemiology/ or epidemiology.mp.

15. burden.mp.

16. 1 or 2 or 3

17. 4 or 5 or 6 or 7 or 8

18. 9 or 10 or 11 or 12 or 13 or 14 or 15

19. 17 or 18

20. 16 and 19

21. limit 20 to yr="January 2020 –June 2022"

CINAHL

S1= (MH “respiratory syncytial virus infections”) OR (MH “respiratory syncytial viruses”) OR “respiratory syncytial virus”

S2= “RSV”

S3= (MH “pneumonia+”) OR “pneumonia” OR (MH “pneumonia, viral”) OR (MH “community-acquired pneumonia”)

S4= (MH “bronchiolitis+”) OR “bronchiolitis”

S5= (MH “respiratory tract infections+”) OR “respiratory infection”

S6= (MH “respiratory tract diseases+”) OR “respiratory disease”

S7= (MH “incidence”) OR “incidence”

S8= (MH “prevalence”) OR “prevalence”

S9= (MH “mortality+”) OR “mortality” OR (MH “infant mortality”) OR (MH “child mortality”) OR (MH “hospital mortality”)

S10= (MH “death+”) OR “death” OR (MH “cause of death”) OR (MH “infant death+”)

S11= (MH “morbidity+”) OR “morbidity”

S12= “burden”

S13= (MH “epidemiology+”) OR “epidemiology”

S14= S1 OR S2

S15= S3 OR S4 OR S5 OR S6

S16= S7 OR S8 OR S9 OR S10 OR S11 OR S12 OR S13

S17= S15 OR S16

S18= S14 AND S17

Limiters: Published year: January 2020–June 2022; Age groups: infant, newborn: birth–1 month, infant: 1–23 months, child, preschool: 2–5 years

---

Web of Science

Topic= (respiratory syncytial virus) AND Topic= (epidemiology) AND Topic= (children)

From January 2020 to June 2022

---

the WHO COVID-19 Global literature on coronavirus disease database

(RSV OR respiratory syncytial virus) AND (respiratory infection* or pneumonia or bronchiolitis) AND (child* or infant*)

LILACS (AMRO/PAHO)

Respiratory syncytial virus in All Indexes

OpenGrey (former: SIGLE)

Respiratory syncytial virus

CNKI

Topic: respiratory infection or pneumonia or bronchiolitis

And Topic: respiratory syncytial virus

And Topic: children

Publication time: January 2020 – June 2022

Wanfang Data

All (vague): respiratory infection or

All (vague): pneumonia or

All (vague): bronchiolitis and

All (vague): respiratory syncytial virus and

All (vague): children

Time: January 2020 – June 2022

Subject field: Medicine and health

CQVIP

Title/key word: respiratory tract infection

Or Title/key word: pneumonia

Or Title/key word: bronchiolitis

And Title/key word: respiratory syncytial virus

And Title/key word: children

Time: January 2020 – June 2022

Subject field: Medicine and health

Text S2. Quality scoring criteria for included studies.

Category	Questions	Scoring criteria
**Study design**	Q1. Whether cases were prospectively enrolled?	• Yes – **1 point**<br>• No/unclear – 0 points
**Study subjects**	Q2. Any subgroup(s) exclusion that may have affected estimates?	• No – **1 point**<br>• Yes/unclear – 0 points (e.g., excluding neonates)
**Case definition**	Q3. Whether common/standard definitions were used?	• Yes – **1 point**<br>• No/unclear – 0 points
**Sampling strategy**	Q4. What is the proportion of eligible ALRI cases that were tested for RSV?	• 90% – **1 point**<br>• <90% but a systematic sample of eligible cases were tested – **1 point**<br>• <90%/unclear – 0 points
**Diagnostic test**	Q5. Whether PCR was used for the confirmation of RSV infection*?	• Yes – **1 point**<br>• No/unclear – 0 points
**<u>For studies reporting hospital admission rate</u>**		
**Adjustment for healthcare utilisation**	Q6. Whether healthcare utilisation was adjusted when calculating hospital admission rate?	• Including all or main hospitals of the area; no need for adjustment – **1 point**<br>• Adjusting for the proportion of patients admitted in the study hospitals – **1 point**<br>• Not including all or main hospitals of the area and not adjusting for healthcare utilisation – 0 point

*Other diagnostic tests might be used but PCR should be used for confirmation, e.g., for negative samples by other tests.

Table S1. Summary of unpublished data from Respiratory Virus Global Epidemiology Network (i.e., previously RSV GEN) investigators

ID	Data sources	Location	Study period	Published reference
**U301**	Markic and colleagues	Split, Croatia (single-centre)	2019/1-2022/5	Mrcela et al. 2022[1]
**U302**	Bassat and colleagues	Spain (25 hospitals, nationwide) †	2019/1-2020/12	Torres-Fernandez et al. 2021[2]
**U303**	Mira-Iglesias and colleagues	Valencia Region, Spain (regional)	2019/1-2020/3; 2020/12-2021/4; 2021/10-2022/5	Mira-Iglesias et al. 2022[3]
**U304**	Cohen and colleagues	Edendale, South Africa (single-centre)	2019/1-2022/3	Update of the previous RSV GEN data (original data id: U113*)[4]
**U305**	Cohen and colleagues	Klerksdorp, South Africa (single-centre)	2019/1-2022/3	Update of the previous RSV GEN data (original data id: U114*)[4]
**U306**	Seo and colleagues	Goyang-si, Gyeonggi-do, Republic of Korea (single-centre)	2019/1-2022/5	Update of the previous RSV GEN data (original data id: U129*)[4]
**U307**	Bont and colleagues	Netherlands (nationwide)	2019/1-2022/5	None
**U308**	Desnoyers and colleagues	Yukon Kuskokwim, Alaska, United States (regional; active surveillance)	2019/11-2022/5	Atwell et al[5]; Hartman et al. 2022[6]
**U309**	Hammitt and colleagues	Southwest United States (regional; active surveillance)	2019/11-2022/5	Atwell et al[5]; Hartman et al. 2022[6]
**U310**	Singleton and colleagues	Yukon Kuskokwim, Alaska, United States (regional, passive surveillance)	2019/1-2022/5	Update of the previous RSV GEN data (original data id: U116*) [4]
**U311**	Chakhunashvili and colleagues	Tbilisi and Kutaisi, Georgia (regional)	2019/1-2022/5	Update of the previous RSV GEN data (original data id: U122*)[4]
**U312**	Heikkinen and colleagues	Turku, Finland (single-centre)	2019/1-2022/5	Update of the previous RSV GEN data (original data id: U102*)[4]
**U313**	Bandeira and colleagues	Lisbon, Portugal (single-centre)	2019/1-2022/5	None
**U314**	Cohen and colleagues	Agincourt and Tintswalo, South Africa (regional)	2019/1-2022/5	None
**U315**	Cohen and colleagues	Helen Joseph and Rahima Moosa, South Africa (regional)	2019/1-2022/5	None
**U316**	Zar and colleagues	Cape Town, South Africa (regional)	2019/1-2022/5	None
**U317**	Nokes and colleagues	Kilifi, Kenya (single-centre)	2019/1-2021/12	Update of the previous RSV GEN data (original data id: U110*) [4]
**U318**	Fu and colleagues	Singapore (single-centre)	2019/1-2022/5	None
**U319–U321**	Danilenko and colleagues	St. Petersburg, Russian Federation (regional)	2019/1-2022/5	Caini et al. 2022[7]
**U322**	Casalegno and colleagues	Lyon, France (single-centre)	2019/1-2022/5	Casalegno et al. 2021[8]
**U323**	Huang and colleagues	Auckland, New Zealand (regional)	2019/5-2019/11; 2020/3-2022/5	Huang et al. 2022[9]

* See pp 9–11 in the appendix of Li et al. Lancet 2022.[4] † In Spain, 25 hospitals from different study sites in Spain were included (retrospective multicentric national study using data obtained from the Pediatric Spanish Society).[2]

Table S2. Methodological details about generalized linear mixed-effects model used for analysing various outcomes.

Outcome of interest	Measure	Within-study likelihood	Link function	Predictors	Intercept
**RSV hospitalisation rate**	incidence rate	Poisson likelihood	log	none	random study-level intercept
**Changes in the distribution of RSV hospitalisations in older age groups**	odds ratio	binomial likelihood	logit	none	fixed intercept
**Changes in the proportion of severe outcomes**	odds ratio	binomial likelihood	logit	none	fixed intercept
**RSV in-hospital case fatality ratio**	proportion	binomial likelihood	logit	none	random study-level intercept

Table S3. Summary of studies that contributed to RSV-associated ALRI hospital admission rate estimates.

Study	Country	Location	Study period	Number of subjects*	Age groups reported	Case definitions	Specimen	Diagnostic test	QA score
**Chiu et al. 2022**[10]	China, Hong Kong	Hong Kong	2017/01–2021/01	65	0–<6m, 6-<12m, 12–<24m, 24–<60m	acute febrile illness or respiratory signs/ symptoms	respiratory samples	PCR	0·67
**Reyes-Dominguez et al. 2021**[11]	Spain	Gran Canaria	2016/01–2021/06	87	0–<24m	RSV acute bronchiolitis	NPW, nasal swabs or aspirate specimens	immunochromatography, PCR	0·60
**Foley et al. 2022**[12]	Australia	Perth	2019/01–2021/03	328	0–<12m, 12–<24m, 24–<48m	symptomatic children	respiratory samples	PCR	0·67
**Markić et al. Unpub**	Croatia	Split	2019/01–2022/05	203	0–<3m, 3–<6m, 6–<9m, 9–<12m, 12–<18m, 18–<24m and 24–<60m	Physician diagnosed	NPA	Rapid antigen tests	0·50
**Mira-Iglesias et al. Unpub†**	Spain	Valencia Region	2019/01–2022/05	286	0–<3m, 3–<6m, 6–<9m, 9–<12m, 12–<18m, 18–<24m and 24–<60m	ILI (WHO)	NPS and/or pharyngeal/nasal swabs	PCR	0·84
**Cohen et al. Unpub**	South Africa	Edendale	2019/01–2022/03	256	0–<3m, 3–<6m, 6–<9m, 9–<12m, 12–<18m, 18–<24m and 24–<60m	Physician diagnosed LRTI	NPS	PCR	0·84
**Cohen et al. Unpub**	South Africa	Klerksdorp	2019/01–2022/03	116	0–<3m, 3–<6m, 6–<9m, 9–<12m, 12–<18m, 18–<24m and 24–<60m	Physician diagnosed LRTI	NPS	PCR	0·84
**Desnoyers et al. Unpub**	United States of America	Yukon Kuskokwim, Alaska (active surveillance)	2019/11–2022/05	125	0–<3m, 3–<6m, 6–<9m, 9–<12m, 12–<18m, 18–<24m and 24–<60m	ARI	MT swab	PCR	0·84
**Hammitt et al. Unpub**	United States of America	Southwest United States (active surveillance)	2019/11–2022/05	93	0–<3m, 3–<6m, 6–<9m, 9–<12m, 12–<18m, 18–<24m and 24–<60m	ARI	MT swab	PCR	0·84
**Singleton et al. Unpub**	United States of America	Yukon Kuskokwim, Alaska (passive surveillance)	2019/01–2022/05	200	0–<3m, 3–<6m, 6–<9m, 9–<12m, 12–<18m, 18–<24m and 24–<36m	ARI hospitalization	NPS	PCR, rapid antigen	0·67
**Heikkinen et al. Unpub**	Finland	Turku	2019/01–2022/05	296	0–<3m, 3–<6m, 6–<9m, 9–<12m, 12–<18m, 18–<24m and 24–<60m	hospitalised Bronchiolitis and/or pneumonia	NPS	Antigen test	0·50
**Nokes et al. Unpub**	Kenya	Kilifi	2019/01–2021/012	235	0–<3m, 3–<6m, 6–<9m, 9–<12m, 12–<18m, 18–<24m and 24–<60m	WHO syndromic pneumonia	NP/OP	Multiplex PCR testing	0·84
**Yung et al. Unpub**	Singapore	Singapore city	2019/01–2022/05	3873	0–<3m, 3–<6m, 6–<9m, 9–<12m, 12–<18m, 18–<24m and 24–<60m	Physician diagnosed	NPS	PCR	0·84

Study	Country	Location	Study period	Number of subjects*	Age groups reported	Case definitions	Specimen	Diagnostic test	QA score
**Casalegno et al. Unpub**	France	Lyon	2019/01–2022/05	1538	0–<3m, 3–<6m, 6–<9m, 9–<12m, 12–<18m, 18–<24m and 24–<60m	WHO definition LRTI	NPS, NPA, BAL	PCR	0·84
**Huang et al. Unpub†**	New Zealand	Auckland	2019/05–2022/05	466	0–<3m, 3–<6m, 6–<9m, 9–<12m, 12–<18m, 18–<24m and 24–<60m	SARI (WHO)	NPS, NPA	PCR	1·00

*Number of RSV associated ALRI hospital admissions; †Studies that tested RSV only during the perceived epidemic months. m = months; RSV = respiratory syncytial virus; ALRI = acute lower respiratory infection; LRTI = lower respiratory tract infection; NPA = nasopharyngeal aspirate; NPS = nasopharyngeal swab; NPW = nasopharyngeal wash; OPS = oropharyngeal swab; BAL = bronchoalveolar lavage; PCR = polymerase chain reaction; SARI = Severe acute respiratory infection; ILI = influenza-like illness; WHO = World Health Organization; QA = quality assessment; Unpub = unpublished data.

Table S4. Summary of studies that contributed to RSV in-hospital case fatality ratio or proportion of requiring supplemental oxygen or ICU admission estimates.

Study	Country	Location	Study period	Number of subjects†	Age groups reported	Case definitions	Specimen	Diagnostic test	QA score
**Markić et al. Unpub**	Croatia	Split	2019/01-2022/05	203	0–<3m, 3–<6m, 6–<9m, 9–<12m, 12–<18m, 18–<24m and 24–<60m	Physician diagnosed	NPA	Rapid antigen tests	0·50
**Bassat et al. Unpub***	Spain	Albacete (Complejo Hospitalario Universitario de Albacete)	2019/01-2020/12	153	0–<24m	Acute bronchiolitis	NPS and/or pharyngeal/nasal swabs	PCR	0·40
**Bassat et al. Unpub***	Spain	Navarra (Complejo Universitario de Navarra)	2019/01-2020/12	126	0–<24m	acute bronchiolitis	NPS and/or pharyngeal/nasal swabs	PCR	0·40
**Bassat et al. Unpub***	Spain	Madrid (Fundacion Jimenez Diaz)	2019/01-2020/12	88	0–<24m	acute bronchiolitis	NPS and/or pharyngeal/nasal swabs	PCR	0·40
**Bassat et al. Unpub***	Spain	Madrid (Hospital 12 de Octubre)	2019/01-2020/12	179	0–<24m	acute bronchiolitis	NPS and/or pharyngeal/nasal swabs	PCR	0·40
**Bassat et al. Unpub***	Spain	Madrid (Hospital Clinico San Carlos)	2019/01-2020/12	69	0–<24m	acute bronchiolitis	NPS and/or pharyngeal/nasal swabs	PCR	0·40
**Bassat et al. Unpub***	Spain	Alicante (Hospital General Universitario de Alicante)	2019/01-2020/12	231	0–<24m	acute bronchiolitis	NPS and/or pharyngeal/nasal swabs	PCR	0·40
**Bassat et al. Unpub***	Spain	Castellon (Hospital General Universitario de Castellon)	2019/01-2020/12	118	0–<24m	acute bronchiolitis	NPS and/or pharyngeal/nasal swabs	PCR	0·40
**Bassat et al. Unpub***	Spain	Madrid (Hospital de Getafe)	2019/01-2020/12	109	0–<24m	acute bronchiolitis	NPS and/or pharyngeal/nasal swabs	PCR	0·40
**Bassat et al. Unpub***	Spain	Madrid (Hospital Infantil Universitario Nino Jesus)	2019/01-2020/12	88	0–<24m	acute bronchiolitis	NPS and/or pharyngeal/nasal swabs	PCR	0·40
**Bassat et al. Unpub***	Spain	Zaragoza (Hospital Miguel Servet de Zaragoza)	2019/01-2020/12	226	0–<24m	acute bronchiolitis	NPS and/or pharyngeal/nasal swabs	PCR	0·40
**Bassat et al. Unpub***	Spain	Tarragona (Hospital Pius de Valls)	2019/01-2020/12	39	0–<24m	acute bronchiolitis	NPS and/or pharyngeal/nasal swabs	PCR	0·40

Study	Country	Location	Study period	Number of subjects†	Age groups reported	Case definitions	Specimen	Diagnostic test	QA score
**Bassat et al. Unpub***	Spain	Madrid (Hospital Sanitas La Zarzuela)	2019/01-2020/12	16	0–<24m	acute bronchiolitis	NPS and/or pharyngeal/nasal swabs	PCR	0·40
**Bassat et al. Unpub***	Spain	Catalonia (Hospital Sant Joan de Deu)	2019/01-2020/12	603	0–<24m	acute bronchiolitis	NPS and/or pharyngeal/nasal swabs	PCR	0·40
**Bassat et al. Unpub***	Spain	Granada (Hospital Universitario Virgen de las Nieves)	2019/01-2020/12	244	0–<24m	acute bronchiolitis	NPS and/or pharyngeal/nasal swabs	PCR	0·40
**Bassat et al. Unpub***	Spain	Madrid (Hospital Universitario QuironSalud)	2019/01-2020/12	63	0–<24m	acute bronchiolitis	NPS and/or pharyngeal/nasal swabs	PCR	0·40
**Bassat et al. Unpub***	Spain	Valladolid (Hospital Universitario Rio Hortega de Valladolid)	2019/01-2020/12	72	0–<24m	acute bronchiolitis	NPS and/or pharyngeal/nasal swabs	PCR	0·40
**Bassat et al. Unpub***	Spain	Madrid (Hospital Universitario de Mostoles)	2019/01-2020/12	82	0–<24m	acute bronchiolitis	NPS and/or pharyngeal/nasal swabs	PCR	0·40
**Bassat et al. Unpub***	Spain	Madrid (Hospital Univesitario de Fuenlabrada)	2019/01-2020/12	105	0–<24m	acute bronchiolitis	NPS and/or pharyngeal/nasal swabs	PCR	0·40
**Bassat et al. Unpub***	Spain	Leon (Hospital de Leon)	2019/01-2020/12	138	0–<24m	acute bronchiolitis	NPS and/or pharyngeal/nasal swabs	PCR	0·40
**Bassat et al. Unpub***	Spain	Extremadura (Hospital de Merida)	2019/01-2020/12	50	0–<24m	acute bronchiolitis	NPS and/or pharyngeal/nasal swabs	PCR	0·40
**Bassat et al. Unpub***	Spain	Andalucia (Hospital Universitario Puerta del Mar)	2019/01-2020/12	79	0–<24m	acute bronchiolitis	NPS and/or pharyngeal/nasal swabs	PCR	0·40
**Bassat et al. Unpub***	Spain	Madrid (Hospital General Universitario de Villalba)	2019/01-2020/12	59	0–<24m	acute bronchiolitis	NPS and/or pharyngeal/nasal swabs	PCR	0·40
**Bassat et al. Unpub***	Spain	Gran Canaria (Hospital Las Palmas de Gran Canaria)	2019/01-2020/12	279	0–<24m	acute bronchiolitis	NPS and/or pharyngeal/nasal swabs	PCR	0·40

Study	Country	Location	Study period	Number of subjects†	Age groups reported	Case definitions	Specimen	Diagnostic test	QA score
**Bassat et al. Unpub***	Spain	Madrid (Hospital Universitario Ramon y Cajal)	2019/01-2020/12	94	0–<24m	acute bronchiolitis	NPS and/or pharyngeal/nasal swabs	PCR	0·40
**Bassat et al. Unpub***	Spain	Catalonia (Hospital Universitario Ramon y Cajal)	2019/01-2020/12	309	0–<24m	acute bronchiolitis	NPS and/or pharyngeal/nasal swabs	PCR	0·40
**Mira-Iglesias et al. Unpub**	Spain	Valencia Region	2019/01-2022/05	286	0–<3m, 3–<6m, 6–<9m, 9–<12m, 12–<18m, 18–<24m and 24–<60m	ILI (WHO)	NPS and/or pharyngeal/nasal swabs	PCR	0·83
**Cohen et al. Unpub**	South Africa	Edendale	2019/01-2022/03	256	0–<3m, 3–<6m, 6–<9m, 9–<12m, 12–<18m, 18–<24m and 24–<60m	Physician diagnosed LRTI	NPS	PCR	0·83
**Cohen et al. Unpub**	South Africa	Klerksdorp	2019/01-2022/03	116	0–<3m, 3–<6m, 6–<9m, 9–<12m, 12–<18m, 18–<24m and 24–<60m	Physician diagnosed LRTI	NPS	PCR	0·83
**Seo et al. Unpub**	Republic of Korea	Goyang-si, Gyeonggi-do	2019/01-2022/05	167	0–<3m, 3–<6m, 6–<9m, 9–<12m, 12–<18m, 18–<24m and 24–<60m	fever, respiratory infection	NPS	PCR	0·80
**Marie-Noelle et al. Unpub***	Netherlands	Netherlands	2019/01-2022/05	740	0–<3m, 3–<6m, 6–<9m, 9–<12m, 12–<18m, 18–<24m and 24–<60m	Systematic testing during COVID-19 pandemic up to the end of 2021. In 2022, only bronchiolitis cases are tested (RSV bronchiolitis, other LRTI, and rhinitis, otitis, tonsillitis)	NPS	Rapid antigen test during the pandemic; Mix of PCR and antigen tests in pre-pandemic years (with a majority of PCR)	0·40
**Desnoyers et al. Unpub**	United States of America	Yukon Kuskokwim, Alaska (active surveillance)	2019/11-2022/05	125	0–<3m, 3–<6m, 6–<9m, 9–<12m, 12–<18m and 18–<24m	ARI	MT swab	PCR	0·83
**Hammitt et al. Unpub**	United States of America	Southwest United States (active surveillance)	2019/11-2022/05	93	0–<3m, 3–<6m, 6–<9m, 9–<12m, 12–<18m, 18–<24m and 24–<60m	ARI	MT swab	PCR	0·83
**Chakhunashvili et al. Unpub**	Georgia	Tbilisi, Kutaisi	2019/01-2022/05	451	0–<3m, 3–<6m, 6–<9m, 9–<12m, 12–<18m, 18–<24m and 24–<60m	SARI	NPS	PCR	0·60
**Heikkinen et al. Unpub**	Finland	Turku	2019/01-2022/05	296	0–<3m, 3–<6m, 6–<9m, 9–<12m, 12–<18m, 18–<24m and 24–<60m	hospitalised Bronchiolitis and/or pneumonia	NPS	Antigen test	0·60

Study	Country	Location	Study period	Number of subjects†	Age groups reported	Case definitions	Specimen	Diagnostic test	QA score
**Bandeira et al. Unpub**	Portugal	Lisbon	2019/01-2022/05	193	0–<3m, 3–<6m, 6–<9m, 9–<12m, 12–<18m, 18–<24m and 24–<60m	Physician diagnosed	NPS	PCR	0·60
**Cohen et al. Unpub**	South Africa	Agincourt and Tintswalo	2019/01-2022/05	103	0–<3m, 3–<6m, 6–<9m, 9–<12m, 12–<18m, 18–<24m and 24–<60m	Physician diagnosed LRTI	NPS	PCR	0·80
**Cohen et al. Unpub**	South Africa	Helen Joseph and Rahima Moosa	2019/01-2022/05	418	0–<3m, 3–<6m, 6–<9m, 9–<12m, 12–<18m, 18–<24m and 24–<60m	Physician diagnosed LRTI	NPS	PCR	0·80
**Zar et al. Unpub**	South Africa	Cape Town	2019/01-2022/05	2725	0–<3m, 3–<6m, 6–<9m, 9–<12m, 12–<18m, 18–<24m and 24–<60m	Physician diagnosed LRTI	NPS	PCR	0·80
**Nokes et al. Unpub**	Kenya	Kilifi	2019/01-2021/12	235	0–<3m, 3–<6m, 6–<9m, 9–<12m, 12–<18m, 18–<24m and 24–<60m	WHO syndromic pneumonia	NP/OP	Multiplex PCR	0·83
**Danilenko et al. Unpub**	Russian Federation	St. Petersburg	2019/01-2022/04	205	0–<3m, 3–<6m, 6–<9m, 9–<12m, 12–<18m, 18–<24m and 24–<60m	ILI (Euro)	NPS	PCR	0·80
**Danilenko et al. Unpub**	Russian Federation	St. Petersburg	2019/01-2022/05	390	0–<3m, 3–<6m, 6–<9m, 9–<12m, 12–<18m, 18–<24m and 24–<60m	ILI (Euro)	NPS	PCR	0·80
**Danilenko et al. Unpub**	Russian Federation	St. Petersburg	2019/01-2022/05	69	0–<3m, 3–<6m, 6–<9m, 9–<12m, 12–<18m, 18–<24m and 24–<60m	SARI (WHO 2011)	NPS	PCR	0·80
**Casalegno et al. Unpub**	France	Lyon	2019/01-2022/05	1538	0–<3m, 3–<6m, 6–<9m, 9–<12m, 12–<18m, 18–<24m and 24–<60m	WHO definition LRTI	NPS, NPA, BAL	PCR	0·83
**Huang et al. Unpub**	New Zealand	Auckland	2019/05-2022/05	283	0–<3m, 3–<6m, 6–<9m, 9–<12m, 12–<18m, 18–<24m and 24–<60m	non-SARI inpatients	NPS, NPA	PCR	0·67
**Huang et al. Unpub**	New Zealand	Auckland	2019/05-2022/05	466	0–<3m, 3–<6m, 6–<9m, 9–<12m, 12–<18m, 18–<24m and 24–<60m	SARI (WHO)	NPS, NPA	PCR	1·00
**Lee et al. 2021***[13]	China, Taiwan Province of China	Zhang hua	2019/10-2021/02	80	0–<48m	hospitalized for wheezing (LRTI)	NPS	PCR	1·00

Study	Country	Location	Study period	Number of subjects†	Age groups reported	Case definitions	Specimen	Diagnostic test	QA score
**Reyes-Dominguez et al. 2021**[11]	Spain	Gran Canaria	2016/01-2021/06	87	0–<24m	RSV acute bronchiolitis	nasal swabs, NPW, NPA	immunochromatography and PCR	0·60
**Guitart et al. 2022**[14]	Spain	Barcelona	2010/09-2021/06	49	0–<12m	severe bronchiolitis admitted to the Paediatric Intensive Care Unit	NPA or a tracheal aspirate/BAL (in intubated patients)	PCR	0·50
**Meyer et al. 2022**[15]	Germany	Cologne	2020/03-2021/11	169	0–<60m	symptomatic children	NPS, OPS	PCR	0·60
**Pappa et al. 2022**[16]	Greece	Thessaloniki and Giannitsa	2021/09-2021/11	41	0–<24m	acute bronchiolitis	NPS	PCR/antigen detection	0·60
**Hernández-Rivas et al. 2021**[17]	Spain	Madrid	2018/09-2021/07	179	0–<60m	hospitalized for RSV	NA	rapid test and/or PCR	0·80
**Fourgeaud et al. 2021**[18]	France	Paris	2018/08-2021/04	212	0–<6m, 6–<12m, 12–<24m	RSV-associated ALRI	nose/throat swabs/BAL	PCR	1·00
**Lin et al. 2022**[19]	China, Taiwan	Xinbei and Gaoxiong	2018/02-2021/01	99	0–<60m	hospitalized with respiratory symptoms	throat swabs or NPA	culture	0·40
**Saravanos et al. 2022***[20]	Australia	Sydney	2014/01-2020/12	713	0–<6m, 6–<12m, 12–<24m, 24–<60m	all RSV-coded hospitalizations and all unspecified bronchiolitis–coded hospitalizations based on principal and additional diagnosis fields	NA	PCR	0·60
**Camporesi et al. 2022**[21]	Italy	Milano, Bologna, Rome and Catania	2021/07-2022/01	87	0–<24m	clinical diagnosis of bronchiolitis or a first episode of acute viral wheeze	NPS	PCR	0·80
**Bermúdez Barrezueta et al. 2022***[22]	Spain	Valladolid	2014/10-2021/09	17	0–<24m	acute bronchiolitis	respiratory samples	molecular diagnostic tests	0·80
**Loconsole et al. 2022**[23]	Italy	Bari	2017/01-2021/12	128	0–<24m	hospitalized with a positive PCR test for RSV	NPS and/or aspirates	PCR	0·40

†Number of RSV-ALRI hospital admissions. ***Studies only reporting proportion of requiring intensive care unit admission among RSV-ALRI in children aged 0–<24 months (retrospective multicentric national study using data obtained from the Pediatric Spanish Society).[2] m = months; RSV = respiratory syncytial virus; ALRI = acute lower respiratory infection; LRTI = lower respiratory tract infection; NPA = nasopharyngeal aspirate; NPS = nasopharyngeal swab; NPW = nasopharyngeal wash; OPS = oropharyngeal swab; BAL = bronchoalveolar lavage; PCR = polymerase chain reaction; SARI = Severe acute respiratory infection; ILI = influenza-like illness; WHO = World Health Organization; NA = not available; QA = quality assessment; Unpub = unpublished data.

Supplementary tables of results

Table S5. Estimates of RSV-associated ALRI hospitalisation burden (hospitalisation rate per 1000 children and number in thousands) in children aged 12–<24 months and 24-<60 months by World Bank income region in different time periods

	2019 (from this study)	2019 (from Li Lancet 2022)[4]	2020	2021†	Latest (April 2021 to March 2022)
**High-income Countries**					
Median of stringency index (IQR)*	0	0	42·0 (39·8, 47·7)	43·8 (42·7, 46·7)	41·5 (37·2, 46·6)
Median changes in mobility (IQR)	0	0	-13·8 (-20·7, -13·7)	-6·0 (-12·3, -1·8)	-5·0 (-9·3, 0·5)
**12–<24m**					
Studies	6	28	8	6	5
Hospital admission rate (95% UI)	4·0 (2·5, 6·7)	4·4 (3·1, 6·1)	0·8 (0·2, 3·1)	3·3 (1·6, 6·7)	4·4 (2·4, 7·8)
Number of episodes (95% UI)	52 (32, 86)	58 (41, 81)	11 (3, 39)	41 (20, 82)	54 (30, 97)
**24–<60m**					
Studies	6	··	7	6	5
Hospital admission rate (95% UI)	0·8 (0·4, 1·6)	··	0·3 (0·1, 1·0)	1·1 (0·5, 2·5)	1·5 (0·8, 2·8)
Number of episodes (95% UI)	34 (17, 65)	··	11 (3, 40)	43 (19, 96)	59 (32, 108)
**Upper-middle-income Countries**					
Median of stringency index (IQR)*	0	0	55·6 (54·1, 55·6)	56·6 (52·6, 56·6)	50·4 (49·1, 50·4)
Median changes in mobility (IQR)	0	0	-16·1 (-23·6, -16·1)	-9·4 (-9·4, -4·6)	-7·8 (-7·8, 3·4)
**12–<24m**					
Studies	3	14	5	5	5
Hospital admission rate (95% UI)	4·9 (0·7, 33·1)	5·1 (2·8, 9·3)	4·7 (0·9, 24·4)	2·2 (1·0, 5·0)	3·3 (1·1, 10·0)
Number of episodes (95% UI)	164 (24, 1106)	207 (113, 376)	148 (29, 760)	65 (29, 145)	95 (31, 294)
**24–<60m**					
Studies	2	··	4	4	4
Hospital admission rate (95% UI)	0·6 (0·1, 3·3)	··	0·3 (0·0, 4·3)	0·3 (0·2, 0·5)	0·4 (0·1, 1·1)
Number of episodes (95% UI)	66 (13, 343)	··	36 (3, 444)	30 (17, 52)	37 (13, 105)
**Lower-middle-income Countries**					
Median of stringency index (IQR)*	0	0	57·4 (57·4, 57·4)	45·7 (45·7, 45·7)	52·8 (52·8, 52·8)
Median changes in mobility (IQR)	0	0	-14·4 (-14·4, -14·4)	19·9 (19·9, 19·9)	··
**12–<24m**					
Studies	1	14	1	1	0
Hospital admission rate (95% UI)	2·0 (1·3, 3·2)	7·6 (4·2,13·8)	2·2 (1·5, 3·5)	2·7 (1·8, 4·0)	··
Number of episodes (95% UI)	138 (88, 217)	466 (256,846)	152 (98, 236)	179 (118, 272)	··
**24–<60m**					
Studies	1	··	1	1	0
Hospital admission rate (95% UI)	0·3 (0·1, 0·6)	··	NE	0·5 (0·3, 0·9)	··
Number of episodes (95% UI)	58 (29, 115)	··	NE	102 (60, 172)	··

UI = uncertainty interval. NE = not estimated. *The median (IQR) COVID-19 NPI stringency index was calculated based on the last month of corresponding year by income region. †The year 2021 (that is from January 2021 to December 2021, overlapping with the latest available period).

Table S6. Estimates of RSV-associated ALRI hospitalisation burden (hospitalisation rate per 1000 children and number in thousands) in children younger than 5 years by Country Development Status in different time periods

	2019 (from this study)	2019 (from Li Lancet 2022)[4]	2020	2021‡
**Industrialised countries**				
Median of stringency index (IQR)*	0	0	63·5 (53·1, 71·1)	43·3 (39·3, 47·5)
Median changes in mobility (IQR)	0	0	-16·1 (-16·1, -14·4)	-9·4 (-9·7, -5·8)
**0–<3m**				
Studies	5	16	5	5
Hospital admission rate (95% UI)	31·6 (20·0, 49·8)	36·9 (20·9, 65·0)	8·0 (1·2, 51·5)	28·4 (14·0, 57·7)
Number of episodes (95% UI)	103 (65, 162)	122 (69, 215)	25 (4, 162)	87 (43, 178)
**3–<6m**				
Studies	5	18	5	5
Hospital admission rate (95% UI)	17·5 (14·8, 20·9)	20·6 (12·4, 34·1)	4·0 (0·9, 16·7)	14·1 (8·0, 25·1)
Number of episodes (95% UI)	57 (48, 68)	68 (41, 113)	13 (3, 53)	43 (24, 77)
**0–<6m†**				
Studies	5	24	5	5
Hospital admission rate (95% UI)	24·4 (17·3, 34·3)	29·3 (20·0, 42·8)	5·2 (0·7, 39·8)	21·2 (10·8, 41·7)
Number of episodes (95% UI)	158 (112, 223)	194 (133, 283)	33 (4, 251)	130 (66, 256)
**6–<12m**				
Studies	5	24	5	5
Hospital admission rate (95% UI)	5·9 (3·8, 9·1)	11·1 (7·1, 17·4)	1·1 (0·4, 3·2)	5·6 (3·4, 9·3)
Number of episodes (95% UI)	38 (24, 59)	74 (47, 116)	7 (2, 20)	35 (21, 57)
**0–<12m†**				
Studies	5	38	6	5
Hospital admission rate (95% UI)	16·0 (12·9, 19·9)	22·5 (17·1, 29·5)	3·6 (0·7, 18·3)	13·6 (7·4, 25·0)
Number of episodes (95% UI)	208 (167, 258)	298 (227, 391)	46 (9, 231)	168 (92, 308)
**12–<60m**				
Studies	5	15	5	5
Hospital admission rate (95% UI)	1·3 (1·0, 1·6)	1·7 (1·3, 2·3)	0·2 (0·0, 1·2)	1·3 (0·7, 2·5)
Number of episodes (95% UI)	68 (53, 87)	95 (72, 125)	12 (2, 65)	66 (34, 129)
**0–<60m†**				
Studies	5	48 (27)	5	5
Hospital admission rate (95% UI)	4·3 (3·7, 5·0)	6·1 (4·7, 7·9)	0·7 (0·1, 6·3)	3·9 (2·1, 7·2)
Number of episodes (95% UI)	287 (246, 333)	413 (318, 537)	45 (5, 415)	252 (137, 463)
**Developing countries**				
Median of stringency index (IQR)*	0	0	52·8 (47·7, 60·8)	45·7 (44·2, 51·1)
Median changes in mobility (IQR)	0	0	-23·4 (-24·1, -21·3)	-4·6 (-8·4, 1·5)
**0–<3m**				
Studies	5	36	7	7
Hospital admission rate (95% UI)	41·2 (19·3, 88·0)	23·5 (15·2,36·3)	19·8 (9·0, 43·4)	14·0 (10·2, 19·0)
Number of episodes (95% UI)	1274 (596, 2723)	721 (466,1115)	602 (274, 1323)	419 (308, 571)
**3–<6m**				
Studies	5	38	7	7
Hospital admission rate (95% UI)	29·2 (12·8, 66·7)	16·7 (11·2,24·9)	16·3 (5·3, 50·2)	9·2 (6·0, 14·0)
Number of episodes (95% UI)	903 (395, 2064)	51 (345, 765)	498 (162, 1529)	275 (180, 420)
**0–<6m†**				
Studies	5	41	8	7
Hospital admission rate (95% UI)	36·0 (16·1, 80·7)	19·3 (13·1,28·6)	15·4 (6·4, 36·8)	11·9 (8·6, 16·4)
Number of episodes (95% UI)	2227 (994, 4989)	1188 (802,1759)	936 (390, 2244)	714 (518, 983)
**6–<12m**				
Studies	5	41	8	7
Hospital admission rate (95% UI)	14·5 (5·8, 36·0)	10·0 (6·9,14·4)	6·7 (1·9, 23·9)	5·8 (2·8, 12·3)
Number of episodes (95% UI)	897 (362, 2226)	612 (422, 886)	408 (114, 1455)	349 (165, 739)
**0–<12m†**				
Studies	5	51	8	7
Hospital admission rate (95% UI)	25·4 (11·3, 57·0)	15·3 (11·3,20·8)	11·4 (4·2, 30·8)	9·1 (5·7, 14·6)
Number of episodes (95% UI)	3138 (1396, 7056)	1881 (1386,2552)	1392 (516, 3757)	1094 (685, 1749)
**12–<60m**				
Studies	4	31	7	6

	2019 (from this study)	2019 (from Li Lancet 2022)[4]	2020	2021‡
Hospital admission rate (95% UI)	1·4 (0·5, 4·2)	1·5 (1·0, 2·3)	1·1 (0·4, 3·3)	1·2 (0·5, 2·6)
Number of episodes (95% UI)	693 (232,2067)	735 (491,1101)	562 (194, 1625)	587 (268, 1289)
**0–<60m†**				
Studies	4	57	7	6
Hospital admission rate (95% UI)	4·8 (2·4, 9·7)	5·2 (3·9, 6·9)	2·6 (1·1, 6·5)	2·6 (1·6, 4·3)
Number of episodes (95% UI)	2974 (1475,5995)	3163 (2395,4179)	1622 (662, 3975)	1578 (960, 2595)
**Global**§				
Median of stringency index (IQR)*	0	0	57·8 (49·0, 70·5)	43·5 (42·8, 48·8)
Median changes in mobility (IQR)	0	0	-16·1 (-23·6, -14·8)	-9·4 (-9·7, -3·4)
**0–<3m**				
Studies	10	52	12	12
Hospital admission rate (95% UI)	40·1 (21·4, 84·1)	24·7 (17·5, 37·1)	18·8 (9·4, 41·1)	15·4 (11·6, 20·6)
Number of episodes (95% UI)	1369 (733, 2875)	841 (597, 1261)	633 (318, 1383)	509 (386, 680)
**3–<6m**				
Studies	10	56	12	12
Hospital admission rate (95% UI)	27·9 (14·0, 62·4)	17·0 (12·4, 24·9)	15·1 (5·6, 46·9)	9·6 (6·9, 14·3)
Number of episodes (95% UI)	952 (477, 2132)	579 (422, 846)	509 (188, 1577)	318 (227, 472)
**0–<6m†**				
Studies	10	65	13	12
Hospital admission rate (95% UI)	34·7 (17·6, 75·9)	20·2 (14·9, 29·1)	14·5 (6·7, 34·6)	12·8 (9·7, 17·2)
Number of episodes (95% UI)	2369 (1202, 5189)	1376 (1017, 1982)	978 (454, 2326)	847 (642, 1137)
**6–<12m**				
Studies	10	65	13	12
Hospital admission rate (95% UI)	13·6 (6·2, 33·4)	10·0 (7·4, 14·3)	6·1 (2·0, 21·9)	5·8 (3·2, 11·8)
Number of episodes (95% UI)	927 (421, 2280)	683 (507, 973)	410 (133, 1473)	382 (209, 784)
**0–<12m†**				
Studies	10	89	14	12
Hospital admission rate (95% UI)	24·3 (12·3, 53·4)	15·9 (12·6, 21·2)	10·7 (4·5, 29·0)	9·5 (6·6, 14·8)
Number of episodes (95% UI)	3317 (1682, 7304)	2170 (1713, 2882)	1443 (601, 3907)	1260 (868, 1958)
**12–<60m**				
Studies	9	46	12	11
Hospital admission rate (95% UI)	1·4 (0·6, 3·9)	1·5 (1·1, 2·2)	1·1 (0·4, 3·1)	1·2 (0·6, 2·5)
Number of episodes (95% UI)	752 (315, 2151)	827 (600, 1207)	574 (223, 1683)	653 (349, 1376)
**0–<60m†**				
Studies	9	105	12	11
Hospital admission rate (95% UI)	4·7 (2·7, 9·2)	5·3 (4·2, 6·8)	2·5 (1·1, 6·1)	2·7 (1·8, 4·3)
Number of episodes (95% UI)	3236 (1832, 6315)	3567 (2856, 4634)	1683 (758, 4119)	1827 (1233, 2908)

UI = uncertainty interval. *The median (IQR) COVID-19 NPI stringency index was calculated based on the last month of corresponding year by income region. †The point estimates and uncertainty interval estimates are not necessarily equal to the sum of the estimates by finer age bands; this is because the studies that contributed to age-specific estimates were different. ‡The year 2021 (that is from January 2021 to December 2021, overlapping with the latest available period). §Global estimates were obtained by summing the numbers of developing and industrialised countries for each of the 1000 samples in the Monte Carlo simulation.

Table S7. Estimates of RSV-associated ALRI hospitalisation burden that requiring mechanical ventilation and ICU admission (hospitalisation rate per 1000 children and number in thousands) in children younger than 5 years by World Bank income region in different time periods

	2019 (from this study)	2019 (from Li Lancet 2022)[4]	2020	2021‡	Latest (April 2021 to March 2022)
**High-income Countries**					
Median of stringency index (IQR)*	0	0	42·0 (39·8, 47·7)	43·8 (42·7, 46·7)	41·5 (37·2, 46·6)
Median changes in mobility (IQR)	0	0	-13·8 (-20·7, -13·7)	-6·0 (-12·3, 1·8)	-5·0 (-9·3, 0·5)
**0–<3m**					
Studies	5	7	5	5	4
Hospital admission rate (95% UI)	3·5 (0·6, 19·0)	4·3 (2·5, 7·1)	0·6 (<0·05, 8·1)	4·3 (0·8, 23·9)	8·2 (2·3, 29·1)
Number of episodes (95% UI)	11 (2, 60)	14 (8, 24)	2 (0, 25)	13 (2, 73)	25 (7, 88)
**3–<6m**					
Studies	5	8	5	5	4
Hospital admission rate (95% UI)	1·1 (0·2, 7·1)	0·9 (0·5, 1·5)	<0·05 (<0·05, 28·7)	0·3 (<0·05, 13·6)	0·8 (<0·05, 17·3)
Number of episodes (95% UI)	3 (0, 22)	3 (2, 5)	0 (0, 88)	1 (0, 41)	2 (0, 53)
**0–<6m†**					
Studies	5	8	5	5	4
Hospital admission rate (95% UI)	2·4 (0·5, 12·1)	2·7 (1·8, 4·3)	0·3 (<0·05, 4·3)	2·4 (0·4, 15·0)	4·9 (1·3, 18·2)
Number of episodes (95% UI)	15 (3, 76)	18 (12, 29)	2 (0, 27)	15 (2, 91)	30 (8, 110)
**6–<12m**					
Studies	5	8	5	5	4
Hospital admission rate (95% UI)	0·4 (0·1, 2·0)	0·3 (0·2, 0·6)	<0·05 (<0·05, 10·6)	0·1 (<0·05, 3·7)	0·3 (<0·05, 3·7)
Number of episodes (95% UI)	3 (1, 12)	2 (1, 4)	0 (0, 65)	1 (0, 22)	2 (0, 22)
**0–<12m†**					
Studies	5	11	5	5	4
Hospital admission rate (95% UI)	1·3 (0·3, 6·7)	1·2 (0·7, 2·1)	0·1 (<0·05, 2·3)	1·3 (0·2, 8·3)	2·6 (0·7, 9·8)
Number of episodes (95% UI)	17 (3, 84)	16 (10, 28)	2 (0, 28)	16 (2, 100)	31 (8, 119)
**12–<60m**					
Studies	5	10	5	5	4
Hospital admission rate (95% UI)	0·1 (<0·05, 0·4)	0·1 (<0·05, 0·1)	<0·05 (<0·05, 0·4)	0·1 (<0·05, 0·6)	0·3 (0·2, 0·6)
Number of episodes (95% UI)	5 (1, 20)	4 (2, 7)	1 (0, 20)	7 (2, 31)	17 (10, 29)
**0–<60m†**					
Studies	5	10	5	5	4
Hospital admission rate (95% UI)	0·3 (0·1, 1·6)	0·3 (0·2, 0·5)	<0·05 (<0·05, 0·6)	0·3 (0·1, 2·2)	0·8 (0·3, 2·4)
Number of episodes (95% UI)	21 (4, 103)	19 (11, 32)	2 (0, 41)	21 (3, 140)	51 (17, 150)
**Upper-middle-income Countries**					
Median of stringency index (IQR)*	0	0	46·0 (46·0, 46·0)	56·6 (56·6, 56·6)	38·6 (38·6, 38·6)
Median changes in mobility (IQR)	0	0	-23·6 (-23·6, -19·0)	-4·6 (-4·6, 7·6)	3·4 (3·4, 3·4)
**0–<3m**					
Studies	2	4	4	4	4
Hospital admission rate (95% UI)	1·3 (0·1,15·6)	3·3 (0·3,34·5)	0·6 (0·2, 2·5)	0·9 (0·1, 5·4)	..
Number of episodes (95% UI)	10 (1,122)	30 (3, 308)	4	4	..
**3–<6m**					
Studies	2	4	4	4	4
Hospital admission rate (95% UI)	1·4 (0·2,11·2)	2·1 (0·2,18·3)	..	1·0 (0·3, 3·1)	0·7 (0·2, 2·6)
Number of episodes (95% UI)	11 (1, 87)	19 (2, 164)	..	7 (2, 21)	4 (1, 18)
**0–<6m†**					
Studies	2	4	4	4	4
Hospital admission rate (95% UI)	0·6 (<0·05,22·8)	2·7 (0·3,26·1)	0·3 (0·1, 1·2)	1·0 (0·4, 2·1)	0·3 (0·1, 1·3)
Number of episodes (95% UI)	10 (0,357)	48 (5, 466)	5 (1, 18)	13 (6, 29)	4 (1, 17)
**6–<12m**					
Studies	2	4	4	4	4
Hospital admission rate (95% UI)	0·7 (0·1, 7·9)	0·8 (<0·05,15·1)	..	0·2 (<0·05, 1·1)	..
Number of episodes (95% UI)	10 (1,124)	13 (1, 269)	..	2 (0, 15)	..
**0–<12m†**					
Studies	2	4	4	4	4

	2019 (from this study)	2019 (from Li Lancet 2022)[4]	2020	2021‡	Latest (April 2021 to March 2022)
Hospital admission rate (95% UI)	0·3 (<0·05,23·1)	1·7 (0·2,19·2)	0·2 (<0·05, 0·6)	0·5 (0·1, 1·8)	0·2 (<0·05, 0·6)
Number of episodes (95% UI)	10 (0,722)	61 (5, 685)	5 (1, 18)	14 (4, 48)	4 (1, 17)
**12–<60m**					
Studies	2	2	4	4	4
Hospital admission rate (95% UI)	..	<0·05 (<0·05, 0·1)	..	..	..
Number of episodes (95% UI)	..	5 (1, 22)	..	..	..
**0–<60m†**					
Studies	2	2	4	4	4
Hospital admission rate (95% UI)	0·1 (<0·05, 4·9)	0·1 (<0·05, 0·8)	<0·05 (<0·05, 0·1)	0·1 (<0·05, 0·3)	<0·05 (<0·05, 0·1)
Number of episodes (95% UI)	11 (0, 833)	14 (1, 144)	5 (1, 20)	15 (4, 54)	5 (1, 19)
**Lower-middle-income Countries**					
Median of stringency index (IQR)*	0	0	57·4 (57·4, 57·4)	45·7 (45·7, 45·7)	52·8 (52·8, 52·8)
Median changes in mobility (IQR)	0	0	-14·4 (-14·4, -14·4)	19·9 (19·9, 19·9)	··
**0–<3m**					
Studies	1	6	1	1	0
Hospital admission rate (95% UI)	1·6 (0·6, 4·3)	19·9 (10·3,38·5)	1·3 (0·4, 4·1)	1·7 (0·6, 4·4)	··
Number of episodes (95% UI)	27 (10, 73)	312 (161, 603)	23 (7, 70)	28 (11, 75)	··
**3–<6m**					
Studies	1	8	1	1	0
Hospital admission rate (95% UI)	0·5 (0·1, 3·2)	7·5 (4·2,13·2)	0·6 (0·1, 4·4)	1·0 (0·3, 4·2)	··
Number of episodes (95% UI)	8 (1, 55)	117 (66, 207)	10 (1, 74)	18 (4, 71)	··
**0–<6m†**					
Studies	1	6	1	1	0
Hospital admission rate (95% UI)	1·3 (0·6, 2·8)	14·8 (8·0,27·5)	1·0 (0·4, 2·8)	1·2 (0·5, 2·8)	··
Number of episodes (95% UI)	44 (20, 97)	464 (250, 862)	35 (13, 93)	39 (16, 94)	··
**6–<12m**					
Studies	1	8	1	1	0
Hospital admission rate (95% UI)	0·2 (<0·05, 1·5)	4·7 (2·2,10·0)	..	0·5 (0·1, 2·1)	··
Number of episodes (95% UI)	7 (1, 52)	147 (69, 313)	..	17 (4, 70)	··
**0–<12m†**					
Studies	1	8	1	1	0
Hospital admission rate (95% UI)	0·8 (0·4, 1·6)	7·2 (3·5,14·9)	0·5 (0·2, 1·3)	0·7 (0·3, 1·6)	··
Number of episodes (95% UI)	51 (24,108)	453 (220, 936)	34 (13, 91)	50 (22,110)	··
**12–<60m**					
Studies	1	6	1	1	0
Hospital admission rate (95% UI)	0·1 (<0·05, 0·2)	0·4 (0·2, 0·9)	..	0·1 (<0·05, 0·3)	··
Number of episodes (95% UI)	22 (7, 67)	98 (45, 214)	..	22 (7, 70)	··
**0–<60m†**					
Studies	1	6	1	1	0
Hospital admission rate (95% UI)	0·2 (0·1, 0·4)	1·4 (0·7, 2·8)	0·1 (<0·05, 0·3)	0·2 (0·1, 0·4)	··
Number of episodes (95% UI)	80 (44,144)	423 (205, 874)	38 (16, 91)	69 (36,132)	··

UI = uncertainty interval. *The median (IQR) COVID-19 NPI stringency index was calculated based on the last month of corresponding year by income region. † The point estimates and uncertainty interval estimates are not necessarily equal to the sum of the estimates by finer age bands; this is because the studies that contributed to age-specific estimates were different. ‡The year 2021 (that is from January 2021 to December 2021, overlapping with the latest available period).

Table S8. Results of sensitivity analysis that only included studies with quality scores ≥0.6

	2019 (from this study)	2019 (from Li Lancet 2022)[4]	2020‡	2021	Latest (April 2021 to March 2022)
**RSV-associated ALRI hospitalisation burden**					
**High-income Countries**					
**0–<3m**					
Studies	4	14	4	4	5
Hospital admission rate (95% UI)	34·5 (20·0, 59·7)	26·6 (14·8,48·0)	3·7 (3·1, 4·5)	23·3 (10·2, 53·2)	36·2 (23·3, 56·2)
Number of episodes (95% UI)	108 (63, 187)	89 (49, 160)	11 (9, 14)	71 (31, 161)	110 (71, 171)
**3–<6m**					
Studies	4	15	4	4	5
Hospital admission rate (95% UI)	21·3 (16·1, 28·1)	16·3 (10·0,26·7)	2·5 (0·5, 13·3)	13·4 (6·6, 27·2)	18·3 (14·4, 23·3)
Number of episodes (95% UI)	67 (50, 88)	54 (33, 89)	8 (1, 41)	41 (20, 82)	56 (44, 71)
**0–<6m†**					
Studies	4	19	5	4	5
Hospital admission rate (95% UI)	28·4 (18·9, 42·7)	22·1 (14·8,33·0)	3·0 (0·6, 15·9)	18·2 (8·3, 40·0)	27·8 (19·2, 40·1)
Number of episodes (95% UI)	178 (118, 268)	148 (99, 220)	18 (4, 98)	110 (50, 243)	169 (117, 243)
**6–<12m**					
Studies	4	19	5	4	5
Hospital admission rate (95% UI)	9·1 (5·0, 16·6)	9·5 (6·1,14·8)	1·3 (0·4, 3·9)	7·7 (4·2, 14·2)	7·7 (6·0, 9·9)
Number of episodes (95% UI)	57 (31, 104)	64 (41, 99)	8 (3, 24)	47 (26, 86)	47 (36, 60)
**0–<12m†**					
Studies	4	23	6	4	5
Hospital admission rate (95% UI)	19·8 (14·7, 26·7)	17·8 (11·9,26·6)	2·5 (0·6, 10·5)	13·2 (6·3, 27·4)	18·2 (14·3, 23·1)
Number of episodes (95% UI)	249 (185, 335)	237 (158, 356)	31 (7, 129)	160 (77, 333)	221 (174, 281)
**12–<60m**					
Studies	4	10	5	4	5
Hospital admission rate (95% UI)	1·9 (0·9, 4·0)	1·7 (1·2, 2·5)	0·3 (0·0, 1·6)	1·8 (0·7, 5·0)	2·3 (1·3, 4·0)
Number of episodes (95% UI)	101 (48, 214)	94 (64, 137)	14 (2, 82)	93 (33, 257)	115 (65, 204)
**0–<60m†**					
Studies	4	27	5	4	5
Hospital admission rate (95% UI)	5·6 (3·8, 8·4)	4·7 (3·2, 7·1)	0·6 (0·1, 4·2)	4·2 (1·8, 9·6)	6·0 (5·4, 6·8)
Number of episodes (95% UI)	368 (247, 548)	322 (216, 482)	36 (5, 274)	267 (117, 611)	381 (339, 429)
**Industrialised Countries**					
**0–<3m**					
Studies	3	11	3	3	4
Hospital admission rate (95% UI)	31·8 (15·6, 64·9)	26·1 (11·9,57·2)	2·3 (0·1, 67·0)	24·1 (8·0, 72·8)	41·9 (26·6, 65·9)
Number of episodes (95% UI)	103 (50, 211)	86 (39, 189)	7 (0, 211)	74 (25, 224)	129 (82, 203)
**3–<6m**					
Studies	3	12	3	3	4
Hospital admission rate (95% UI)	18·6 (16·0, 21·5)	14·8 (7·6,28·6)	1·4 (0·1, 16·0)	11·9 (4·6, 31·2)	19·4 (14·6, 25·9)
Number of episodes (95% UI)	60 (52, 70)	49 (25, 95)	4 (0, 51)	37 (14, 96)	60 (45, 80)
**0–<6m†**					
Studies	3	16	3	3	4
Hospital admission rate (95% UI)	25·6 (15·6, 42·3)	21·7 (13·4,35·3)	1·4 (0·0, 52·9)	17·8 (6·2, 51·3)	31·0 (21·0, 45·9)
Number of episodes (95% UI)	166 (101, 274)	144 (88, 233)	9 (0, 334)	109 (38, 316)	191 (129, 282)
**6–<12m**					
Studies	3	16	3	3	4
Hospital admission rate (95% UI)	7·0 (4·1, 11·8)	9·1 (5·4,15·4)	0·6 (0·1, 5·1)	6·3 (3·1, 13·1)	31·0 (21·0, 45·9)
Number of episodes (95% UI)	45 (27, 77)	60 (36, 102)	4 (0, 32)	39 (19, 80)	191 (129, 282)
**0–<12m†**					
Studies	3	20	4	3	4
Hospital admission rate (95% UI)	17·4 (13·3, 22·8)	17·8 (11·2,28·3)	1·6 (0·1, 21·2)	12·2 (4·6, 32·4)	31·0 (21·0, 45·9)
Number of episodes (95% UI)	226 (173, 296)	235 (148, 375)	20 (2, 267)	150 (57, 399)	191 (129, 282)
**12–<60m**					
Studies	3	8	3	3	4
Hospital admission rate (95% UI)	1·3 (0·9, 1·8)	2·0 (1·3, 2·9)	0·1 (0·0, 0·6)	1·2 (0·5, 3·2)	1·8 (1·1, 3·1)
Number of episodes (95% UI)	68 (48, 98)	109 (74, 161)	4 (0, 33)	62 (23, 167)	94 (55, 161)
**0–<60m†**					
Studies	3	24	3	3	4

	2019 (from this study)	2019 (from Li Lancet 2022)[4]	2020‡	2021	Latest (April 2021 to March 2022)
Hospital admission rate (95% UI)	4·5 (3·9, 5·2)	4·7 (3·0, 7·4)	0·2 (0·0, 7·6)	3·4 (1·3, 9·0)	5·8 (5·1, 6·5)
Number of episodes (95% UI)	303 (262, 350)	320 (204, 503)	10 (0, 499)	215 (80, 578)	368 (327, 415)
**Global**[§]					
**0–<3m**					
Studies	8	43	10	10	0
Hospital admission rate (95% UI)	40·0 (21·5, 83·8)	22·5 (15·4, 34·8)	18·5 (8·8, 43·6)	15·2 (11·1, 21·4)	··
Number of episodes (95% UI)	1369 (734, 2864)	764 (523, 1183)	622 (298, 1465)	504 (368, 708)	··
**3–<6m**					
Studies	8	46	10	10	0
Hospital admission rate (95% UI)	28·0 (14·1, 62·5)	15·0 (10·6, 22·3)	14·9 (5·4, 46·9)	9·5 (6·6, 14·1)	··
Number of episodes (95% UI)	956 (481, 2135)	509 (362, 759)	501 (181, 1577)	315 (219, 468)	··
**0–<6m†**					
Studies	8	53	11	10	0
Hospital admission rate (95% UI)	34·8 (17·9, 76·8)	18·1 (13·1, 26·7)	14·3 (6·3, 36·7)	12·6 (9·3, 17·4)	··
Number of episodes (95% UI)	2380 (1221, 5247)	1234 (891, 1820)	964 (425, 2467)	836 (616, 1153)	··
**6–<12m**					
Studies	8	53	11	10	0
Hospital admission rate (95% UI)	13·7 (6·3, 33·5)	8·7 (6·4, 12·4)	6·1 (1·9, 22·0)	5·9 (3·2, 11·9)	··
Number of episodes (95% UI)	937 (432, 2292)	590 (436, 843)	410 (130, 1481)	388 (213, 791)	··
**0–<12m†**					
Studies	8	66	12	10	0
Hospital admission rate (95% UI)	24·4 (12·4, 53·6)	14·4 (11·2, 19·5)	10·6 (4·3, 28·8)	9·5 (6·4, 14·7)	··
Number of episodes (95% UI)	3336 (1693, 7329)	1959 (1518, 2647)	1422 (576, 3879)	1254 (843, 1951)	··
**12–<60m**					
Studies	7	37	10	9	0
Hospital admission rate (95% UI)	1·4 (0·6, 3·9)	1·5 (1·1, 2·2)	1·0 (0·4, 3·0)	1·2 (0·6, 2·6)	··
Number of episodes (95% UI)	754 (320, 2154)	818 (594, 1201)	564 (215, 1654)	650 (344, 1379)	··
**0–<60m†**					
Studies	7	76	10	9	0
Hospital admission rate (95% UI)	4·7 (2·7, 9·2)	4·8 (3·8, 6·4)	2·4 (1·1, 6·3)	2·7 (1·8, 4·3)	··
Number of episodes (95% UI)	3253 (1847, 6332)	3283 (2599, 4333)	1654 (719, 4273)	1805 (1191, 2894)	··
**RSV-associated ALRI hospitalisation burden requiring mechanical ventilation or ICU admission**					
**High-income Countries**					
**0–<3m**					
Studies	3	··	3	3	4
Hospital admission rate (95% UI)	5·7 (0·8, 39·7)	··	0·1 (<0·05, 163·7)	3·0 (0·1, 106·4)	8·2 (2·3, 29·1)
Number of episodes (95% UI)	18 (3, 125)	··	0 (0, 503)	9 (0, 323)	25 (7, 88)
**3–<6m**					
Studies	3	··	3	3	4
Hospital admission rate (95% UI)	3·0 (0·7, 13·6)	··	0·1 (<0·05, 21·8)	1·7 (0·1, 31·8)	0·8 (<0·05, 17·3)
Number of episodes (95% UI)	9 (2, 43)	··	0 (0, 67)	5 (0, 97)	2 (0, 53)
**0–<6m†**					
Studies	3	··	3	3	4
Hospital admission rate (95% UI)	4·4 (0·8, 25·7)	··	<0·05 (<0·05, 117·9)	1·9 (<0·05, 78·8)	4·9 (1·3, 18·2)
Number of episodes (95% UI)	28 (5, 161)	··	0 (0, 725)	11 (0, 479)	30 (8, 110)
**6–<12m**					
Studies	3	··	3	3	4
Hospital admission rate (95% UI)	0·9 (0·3, 2·7)	··	0·1 (<0·05, 8·8)	0·5 (<0·05, 7·8)	0·3 (<0·05, 3·7)
Number of episodes (95% UI)	6 (2, 17)	··	0 (0, 54)	3 (0, 47)	2 (0, 22)
**0–<12m†**					
Studies	3	··	3	3	4
Hospital admission rate (95% UI)	2·6 (0·5, 13·9)	··	<0·05 (<0·05, 84·3)	1·0 (<0·05, 46·3)	2·6 (0·7, 9·8)
Number of episodes (95% UI)	33 (6, 174)	··	0 (0, 1037)	12 (0, 562)	31 (8, 119)
**12–<60m**					
Studies	3	··	3	3	4
Hospital admission rate (95% UI)	0·2 (<0·05, 0·7)	··	<0·05 (<0·05, 1·5)	0·1 (<0·05, 1·9)	0·3 (0·2, 0·6)
Number of episodes (95% UI)	8 (2, 34)	··	0 (0, 81)	7 (1, 96)	17 (10, 29)

	2019 (from this study)	2019 (from Li Lancet 2022)[4]	2020‡	2021	Latest (April 2021 to March 2022)
**0–<60m†**					
Studies	3	··	3	3	4
Hospital admission rate (95% UI)	0·7 (0·1, 3·2)	··	<0·05 (<0·05, 40·7)	0·2 (<0·05, 12·6)	0·8 (0·3, 2·4)
Number of episodes (95% UI)	43 (9, 208)	··	0 (0, 2622)	15 (0, 799)	51 (17, 150)
**RSV-associated ALRI in-hospital CFR**					
**High-income Countries**					
**0–<12m**					
Studies	6	23	8		
In-hospital CFR (%)	0·1 (<0·05, 1·4)	0·1 (0·1, 0·3)	··		
**12–<60m**					
Studies	5	17	6		
In-hospital CFR (%)	0·3 (<0·05, 2·4)	0·2 (0·1, 0·5)	0·2 (<0·05, 1·5)		
**0–<60m**					
Studies	5	15	8		
In-hospital CFR (%)	0·1 (<0·05, 0·6)	0·1 (0·1, 0·3)	<0·05 (<0·05, 0·3)		

Note that studies with quality score < 0.6 were all from high-income countries/industrialised countries (i.e., two studies from Croatia and Finland). Hospitalisation rates were shown in per 1000 children and number in thousands. UI = uncertainty interval. †The point estimates and uncertainty interval estimates are not necessarily equal to the sum of the estimates by finer age bands; this is because the studies that contributed to age-specific estimates were different. ‡For in-hospital CFR, 2020 time period means 2019 and 2020 onwards because no further stratification due to data scarcity. § Global estimates were obtained by summing the numbers of developing and industrialised countries for each of the 1000 samples in the Monte Carlo simulation.

Table S9. Results of sensitivity analysis that excluded studies with non-year-round testing

	2019 (from this study)	2020‡	2021	Latest (April 2021 to March 2022)
**RSV-associated ALRI hospitalisation burden**				
**High-income Countries**				
**0–<3m**				
Studies	4	4	4	5
Hospital admission rate (95% UI)	42·4 (30·5, 58·9)	19·6 (10·8, 35·7)	38·5 (21·9, 67·6)	36·2 (23·3, 56·2)
Number of episodes (95% UI)	133 (96, 185)	60 (33, 110)	117 (66, 205)	110 (71, 171)
**3–<6m**				
Studies	4	4	4	5
Hospital admission rate (95% UI)	19·8 (13·2, 29·7)	8·6 (5·1, 14·3)	20·9 (15·6, 27·9)	18·3 (14·4, 23·3)
Number of episodes (95% UI)	62 (41, 93)	26 (16, 44)	63 (48, 85)	56 (44, 71)
**0–<6m†**				
Studies	4	5	4	5
Hospital admission rate (95% UI)	30·9 (22·3, 42·9)	11·4 (6·1, 21·3)	30·0 (19·2, 47·1)	27·8 (19·2, 40·1)
Number of episodes (95% UI)	194 (140, 269)	70 (37, 131)	182 (116, 286)	169 (117, 243)
**6–<12m**				
Studies	4	5	4	5
Hospital admission rate (95% UI)	6·2 (2·9, 13·4)	2·3 (1·3, 3·8)	7·6 (4·7, 12·4)	7·7 (6·0, 9·9)
Number of episodes (95% UI)	39 (18, 84)	14 (8, 24)	46 (29, 75)	47 (36, 60)
**0–<12m†**				
Studies	4	5	4	5
Hospital admission rate (95% UI)	19·3 (13·6, 27·2)	7·1 (4·2, 12·0)	19·4 (13·7, 27·5)	18·2 (14·3, 23·1)
Number of episodes (95% UI)	242 (171, 341)	87 (52, 148)	236 (166, 334)	221 (174, 281)
**12–<60m**				
Studies	4	5	4	5
Hospital admission rate (95% UI)	1·7 (0·8, 3·8)	1·0 (0·5, 2·0)	2·2 (1·0, 4·5)	2·3 (1·3, 4·0)
Number of episodes (95% UI)	91 (41, 202)	52 (27, 102)	111 (53, 228)	115 (65, 204)
**0–<60m†**				
Studies	4	5	4	5
Hospital admission rate (95% UI)	5·4 (3·5, 8·6)	2·5 (1·7, 3·6)	6·4 (5·0, 8·1)	6·0 (5·4, 6·8)
Number of episodes (95% UI)	356 (227, 560)	159 (110, 231)	403 (317, 511)	381 (339, 429)
**Industrialised Countries**				
**0–<3m**				
Studies	3	3	3	4
Hospital admission rate (95% UI)	41·3 (26·2, 65·1)	26·6 (20·1, 35·2)	48·1 (27·3, 84·7)	41·9 (26·6, 65·9)
Number of episodes (95% UI)	134 (85, 211)	84 (63, 111)	148 (84, 261)	129 (82, 203)
**3–<6m**				
Studies	3	3	3	4
Hospital admission rate (95% UI)	17·3 (12·0, 24·8)	8·6 (4·2, 17·6)	22·6 (15·7, 32·5)	19·4 (14·6, 25·9)
Number of episodes (95% UI)	56 (39, 80)	27 (13, 56)	69 (48, 100)	60 (45, 80)
**0–<6m†**				
Studies	3	3	3	4
Hospital admission rate (95% UI)	28·4 (18·6, 43·4)	18·5 (12·8, 26·8)	35·3 (21·9, 57·0)	31·0 (21·0, 45·9)
Number of episodes (95% UI)	184 (121, 282)	117 (81, 169)	217 (134, 351)	191 (129, 282)
**6–<12m**				
Studies	3	3	3	4
Hospital admission rate (95% UI)	4·1 (3·2, 5·3)	1·9 (1·3, 2·7)	6·3 (3·9, 10·2)	7·1 (5·3, 9·5)
Number of episodes (95% UI)	27 (21, 34)	12 (8, 17)	39 (24, 63)	43 (32, 58)
**0–<12m†**				
Studies	3	3	3	4
Hospital admission rate (95% UI)	16·7 (11·9, 23·4)	10·3 (7·2, 14·7)	20·6 (13·2, 32·2)	19·6 (15·1, 25·3)
Number of episodes (95% UI)	217 (155, 304)	130 (91, 185)	254 (162, 397)	241 (186, 312)
**12–<60m**				
Studies	3	3	3	4
Hospital admission rate (95% UI)	1·0 (0·7, 1·3)	0·7 (0·3, 1·8)	1·5 (1·0, 2·5)	1·8 (1·1, 3·1)
Number of episodes (95% UI)	54 (41, 72)	38 (15, 97)	79 (49, 128)	94 (55, 161)
**0–<60m†**				
Studies	3	3	3	4
Hospital admission rate (95% UI)	4·3 (3·3, 5·6)	2·8 (1·7, 4·5)	5·8 (4·5, 7·5)	5·8 (5·1, 6·5)
Number of episodes (95% UI)	291 (225, 377)	182 (112, 297)	371 (287, 480)	368 (327, 415)

	2019 (from this study)	2020‡	2021	Latest (April 2021 to March 2022)
**Global§**				
**0–<3m**				
Studies	8	10	10	0
Hospital admission rate (95% UI)	41·0 (22·3, 84·8)	20·3 (11·1, 42·5)	17·3 (13·1, 22·8)	··
Number of episodes (95% UI)	1400 (764, 2899)	682 (374, 1430)	572 (434, 754)	··
**3–<6m**				
Studies	8	10	10	0
Hospital admission rate (95% UI)	27·9 (13·8, 62·5)	15·6 (6·0, 46·9)	10·4 (7·6, 15·0)	··
Number of episodes (95% UI)	952 (471, 2135)	523 (203, 1576)	343 (253, 498)	··
**0–<6m†**				
Studies	8	11	10	0
Hospital admission rate (95% UI)	35·1 (18·1, 76·8)	15·6 (7·9, 35·7)	14·1 (10·8, 18·5)	··
Number of episodes (95% UI)	2397 (1240, 5252)	1046 (533, 2404)	934 (716, 1226)	··
**6–<12m**				
Studies	8	11	10	0
Hospital admission rate (95% UI)	13·4 (6·0, 33·1)	6·2 (2·0, 21·9)	5·8 (3·2, 11·9)	··
Number of episodes (95% UI)	917 (412, 2264)	415 (137, 1476)	385 (213, 788)	··
**0–<12m†**				
Studies	8	11	10	0
Hospital admission rate (95% UI)	24·4 (12·3, 53·6)	11·2 (5·1, 29·1)	10·1 (7·2, 15·4)	··
Number of episodes (95% UI)	3332 (1679, 7327)	1511 (685, 3921)	1343 (951, 2043)	··
**12–<60m**				
Studies	7	10	9	0
Hospital admission rate (95% UI)	1·3 (0·5, 3·9)	1·1 (0·5, 3·1)	1·2 (0·7, 2·6)	··
Number of episodes (95% UI)	738 (302, 2136)	599 (249, 1710)	662 (362, 1386)	··
**0–<60m†**				
Studies	7	10	9	0
Hospital admission rate (95% UI)	4·7 (2·7, 9·3)	2·6 (1·3, 6·2)	2·9 (2·0, 4·5)	··
Number of episodes (95% UI)	3244 (1833, 6351)	1794 (888, 4240)	1941 (1364, 3005)	··
**RSV-associated ALRI hospitalisation burden requiring mechanical ventilation or ICU admission**				
**High-income Countries**				
**0–<3m**				
Studies	3	3	3	4
Hospital admission rate (95% UI)	4·8 (0·2, 104·0)	3·7 (0·7, 19·6)	11·8 (2·5, 56·8)	8·2 (2·3, 29·1)
Number of episodes (95% UI)	15 (1, 326)	11 (2, 60)	36 (7, 173)	25 (7, 88)
**3–<6m**				
Studies	3	3	3	4
Hospital admission rate (95% UI)	0·2 (<0·05, 112·5)	0·4 (<0·05, 24·2)	0·2 (0·2, 0·2)	0·8 (<0·05, 17·3)
Number of episodes (95% UI)	1 (0, 353)	1 (0, 74)	1 (1, 1)	2 (0, 53)
**0–<6m†**				
Studies	3	3	3	4
Hospital admission rate (95% UI)	2·5 (0·1, 64·9)	1·9 (0·3, 11·0)	6·5 (1·2, 36·5)	4·9 (1·3, 18·2)
Number of episodes (95% UI)	16 (1, 407)	12 (2, 67)	39 (7, 221)	30 (8, 110)
**6–<12m**				
Studies	3	3	3	4
Hospital admission rate (95% UI)	0·1 (<0·05, 19·0)	0·2 (<0·05, 9·4)	0·1 (<0·05, 44·8)	0·3 (<0·05, 3·7)
Number of episodes (95% UI)	1 (0, 119)	1 (0, 58)	1 (0, 272)	2 (0, 22)
**0–<12m†**				
Studies	3	3	3	4
Hospital admission rate (95% UI)	1·2 (<0·05, 34·3)	1·0 (0·2, 5·9)	3·4 (0·6, 20·2)	2·6 (0·7, 9·8)
Number of episodes (95% UI)	16 (1, 431)	12 (2, 73)	41 (7, 245)	31 (8, 119)
**12–<60m**				
Studies	3	3	3	4
Hospital admission rate (95% UI)	0·1 (<0·05, 0·9)	0·2 (0·1, 0·3)	0·3 (0·1, 1·1)	0·3 (0·2, 0·6)
Number of episodes (95% UI)	6 (1, 46)	9 (6, 14)	13 (3, 56)	17 (10, 29)
**0–<60m†**				
Studies	3	3	3	4
Hospital admission rate (95% UI)	0·3 (<0·05, 7·7)	0·3 (0·1, 1·4)	0·9 (0·2, 4·5)	0·8 (0·3, 2·4)
Number of episodes (95% UI)	18 (1, 507)	18 (4, 91)	57 (11, 285)	51 (17, 150)
**RSV-associated ALRI in-hospital CFR**				

	2019 (from this study)	2020‡	2021	Latest (April 2021 to March 2022)
**<u>High-income Countries</u>**				
**0–<12m**				
Studies	6	9		
In-hospital CFR (%)	0·2 (<0·05, 1.5)	<0·05 (<0·05, 2·0)		
**12–<60m**				
Studies	5	6		
In-hospital CFR (%)	0·5 (0·1, 3·6)	0·3 (<0·05, 6.8)		
**0–<60m**				
Studies	5	9		
In-hospital CFR (%)	0·2 (<0·05, 0·7)	0.1 (<0·05, 0·8)		

Note that studies with incomplete surveillance year were all from high-income countries/industrialised countries (i.e., two studies from Spain and New Zealand). Hospitalisation rates were shown in per 1000 children and number in thousands. UI = uncertainty interval. †The point estimates and uncertainty interval estimates are not necessarily equal to the sum of the estimates by finer age bands; this is because the studies that contributed to age-specific estimates were different. ‡For in-hospital CFR, 2020 time period means 2019 and 2020 onwards because no further stratification due to data scarcity. § Global estimates were obtained by summing the numbers of developing and industrialised countries for each of the 1000 samples in the Monte Carlo simulation.

Table S10. Results of sensitivity analysis that reclassified studies focusing on indigenous populations as high-income/industrialised countries

	2019 (from this study)	2020‡	2021	Latest (April 2021 to March 2022)
**RSV-associated ALRI hospitalisation burden**				
**High-income Countries**				
**0–<3m**				
Studies	7	9	9	5
Hospital admission rate (95% UI)	41·5 (24·6, 70·0)	16·2 (5·4, 49·0)	23·7 (14·3, 39·2)	36·2 (23·3, 56·2)
Number of episodes (95% UI)	130 (77, 220)	50 (17, 151)	72 (43, 119)	110 (71, 171)
**3–<6m**				
Studies	7	9	9	5
Hospital admission rate (95% UI)	24·0 (13·5, 42·8)	11·4 (3·5, 37·5)	13·0 (8·4, 20·1)	18·3 (14·4, 23·3)
Number of episodes (95% UI)	75 (42, 134)	35 (11, 115)	40 (26, 61)	56 (44, 71)
**0–<6m†**				
Studies	7	10	9	5
Hospital admission rate (95% UI)	33·8 (19·7, 58·0)	11·9 (3·8, 37·6)	18·2 (11·3, 29·3)	27·8 (19·2, 40·1)
Number of episodes (95% UI)	212 (123, 364)	73 (23, 231)	110 (68, 178)	169 (117, 243)
**6–<12m**				
Studies	7	10	9	5
Hospital admission rate (95% UI)	10·0 (4·5, 22·3)	3·9 (1·0, 14·5)	7·1 (4·3, 11·6)	7·7 (6·0, 9·9)
Number of episodes (95% UI)	63 (28, 140)	24 (6, 89)	43 (26, 71)	47 (36, 60)
**0–<12m†**				
Studies	7	11	9	5
Hospital admission rate (95% UI)	22·8 (13·3, 39·1)	8·2 (2·6, 25·2)	13·0 (8·2, 20·6)	18·2 (14·3, 23·1)
Number of episodes (95% UI)	287 (167, 491)	100 (33, 309)	158 (100, 250)	221 (174, 281)
**12–<60m**				
Studies	6	9	8	5
Hospital admission rate (95% UI)	1·7 (1·0, 2·8)	0·8 (0·2, 2·7)	1·6 (0·9, 2·9)	2·3 (1·3, 4·0)
Number of episodes (95% UI)	88 (51, 151)	40 (11, 143)	84 (47, 151)	115 (65, 204)
**0–<60m†**				
Studies	6	9	8	5
Hospital admission rate (95% UI)	5·0 (3·6, 6·8)	1·8 (0·5, 6·9)	4·0 (2·5, 6·2)	6·0 (5·4, 6·8)
Number of episodes (95% UI)	325 (235, 448)	119 (32, 444)	252 (160, 395)	381 (339, 429)
**Upper-middle-income Countries**				
**0–<3m**				
Studies	2	2	2	5
Hospital admission rate (95% UI)	25·7 (8·7, 75·5)	7·7 (5·0, 11·8)	10·9 (7·6, 15·5)	20·5 (6·3, 67·4)
Number of episodes (95% UI)	201 (68, 590)	56 (37, 87)	74 (52, 106)	140 (43, 459)
**3–<6m**				
Studies	2	2	2	5
Hospital admission rate (95% UI)	14·8 (5·4, 40·2)	2·5 (1·0, 6·7)	7·0 (4·3, 11·3)	11·1 (2·8, 43·6)
Number of episodes (95% UI)	115 (42, 314)	19 (7, 49)	48 (30, 77)	76 (19, 297)
**0–<6m†**				
Studies	2	2	2	5
Hospital admission rate (95% UI)	20·2 (7·1, 57·6)	5·2 (3·6, 7·5)	9·1 (6·8, 12·0)	15·9 (4·7, 53·7)
Number of episodes (95% UI)	316 (111, 900)	76 (52, 110)	123 (93, 164)	216 (64, 732)
**6–<12m**				
Studies	2	2	2	5
Hospital admission rate (95% UI)	6·4 (3·2, 12·8)	0·7 (0·3, 2·0)	2·4 (1·4, 4·1)	7·2 (1·8, 28·8)
Number of episodes (95% UI)	100 (50, 200)	11 (4, 29)	33 (19, 56)	98 (24, 393)
**0–<12m†**				
Studies	2	2	2	5
Hospital admission rate (95% UI)	13·4 (5·1, 35·1)	2·8 (1·8, 4·4)	5·6 (4·4, 7·3)	11·7 (3·3, 40·8)
Number of episodes (95% UI)	420 (161, 1097)	82 (53, 129)	154 (119, 198)	318 (91, 1112)
**12–<60m**				
Studies	2	2	2	4
Hospital admission rate (95% UI)	0·9 (0·3, 3·3)	0·2 (0·1, 0·4)	0·4 (0·3, 0·7)	0·8 (0·3, 2·7)
Number of episodes (95% UI)	127 (36, 450)	25 (12, 50)	56 (36, 88)	109 (35, 343)

	2019 (from this study)	2020‡	2021	Latest (April 2021 to March 2022)
**0–<60m†**				
Studies	2	2	2	4
Hospital admission rate (95% UI)	3·4 (1·2, 9·7)	0·7 (0·4, 1·2)	1·5 (1·2, 1·8)	2·1 (0·7, 6·1)
Number of episodes (95% UI)	581 (206, 1643)	108 (60, 192)	227 (182, 283)	334 (117, 956)
**RSV-associated ALRI hospitalisation burden requiring mechanical ventilation or ICU admission**				
**High-income Countries**				
**0–<3m**				
Studies	5	7	7	4
Hospital admission rate (95% UI)	3·5 (0·6, 19·0)	0·4 (<0·05, 5·3)	2·2 (0·3, 14·7)	8·2 (2·3, 29·1)
Number of episodes (95% UI)	11 (2, 60)	1 (0, 16)	7 (1, 45)	25 (7, 88)
**3–<6m**				
Studies	5	7	7	4
Hospital admission rate (95% UI)	1·1 (0·2, 7·1)	<0·05 (<0·05, 6<0·05)	0·2 (<0·05, 9·4)	0·8 (<0·05, 17·3)
Number of episodes (95% UI)	3 (0, 22)	0 (0, 184)	0 (0, 29)	2 (0, 53)
**0–<6m†**				
Studies	5	7	7	4
Hospital admission rate (95% UI)	2·4 (0·5, 12·1)	0·2 (<0·05, 2·8)	1·2 (0·2, 8·8)	4·9 (1·3, 18·2)
Number of episodes (95% UI)	15 (3, 76)	1 (0, 17)	7 (1, 53)	30 (8, 110)
**6–<12m**				
Studies	5	7	7	4
Hospital admission rate (95% UI)	0·4 (0·1, 2·0)	<0·05 (<0·05, 17·4)	0·1 (<0·05, 2·9)	0·3 (<0·05, 3·7)
Number of episodes (95% UI)	3 (1, 12)	0 (0, 107)	0 (0, 17)	2 (0, 22)
**0–<12m†**				
Studies	5	7	7	4
Hospital admission rate (95% UI)	1·3 (0·3, 6·7)	0·1 (<0·05, 1·4)	0·6 (0·1, 4·7)	2·6 (0·7, 9·8)
Number of episodes (95% UI)	17 (3, 84)	1 (0, 18)	7 (1, 57)	31 (8, 119)
**12–<60m**				
Studies	5	7	7	4
Hospital admission rate (95% UI)	0·1 (<0·05, 0·4)	<0·05 (<0·05, 0·3)	0·1 (<0·05, 0·4)	0·3 (0·2, 0·6)
Number of episodes (95% UI)	5 (1, 20)	1 (0, 18)	5 (1, 22)	17 (10, 29)
**0–<60m†**				
Studies	5	7	7	4
Hospital admission rate (95% UI)	0·3 (0·1, 1·6)	<0·05 (<0·05, 0·4)	0·1 (<0·05, 1·2)	0·8 (0·3, 2·4)
Number of episodes (95% UI)	21 (4, 103)	1 (0, 24)	9 (1, 77)	51 (17, 150)
**Upper-middle-income Countries**				
**0–<3m**				
Studies	2	2	2	4
Hospital admission rate (95% UI)	1·3 (0·1, 15·6)	0·7 (0·2, 2·9)	1·1 (0·4, 3·4)	..
Number of episodes (95% UI)	10 (1, 122)	5 (1, 21)	7 (2, 23)	..
**3–<6m**				
Studies	2	2	2	4
Hospital admission rate (95% UI)	1·4 (0·2, 11·2)	..	1·2 (0·4, 3·8)	0·7 (0·2, 2·6)
Number of episodes (95% UI)	11 (1, 87)	..	8 (3, 26)	4 (1, 18)
**0–<6m†**				
Studies	2	2	2	4
Hospital admission rate (95% UI)	0·6 (<0·05, 22·8)	0·4 (0·1, 1·5)	1·2 (0·5, 2·6)	0·3 (0·1, 1·3)
Number of episodes (95% UI)	10 (0, 357)	5 (1, 22)	16 (7, 35)	4 (1, 17)
**6–<12m**				
Studies	2	2	2	4
Hospital admission rate (95% UI)	0·7 (0·1, 7·9)	..	0·2 (<0·05, 1·3)	..
Number of episodes (95% UI)	10 (1, 124)	..	3 (0, 18)	..
**0–<12m†**				
Studies	2	2	2	4
Hospital admission rate (95% UI)	0·3 (<0·05, 23·1)	0·2 (<0·05, 0·7)	0·7 (0·3, 1·4)	0·2 (<0·05, 0·6)
Number of episodes (95% UI)	10 (0, 722)	5 (1, 22)	18 (9, 38)	4 (1, 17)
**12–<60m**				
Studies	2	2	2	4

	2019 (from this study)	2020‡	2021	Latest (April 2021 to March 2022)
Hospital admission rate (95% UI)	..	..	..	..
Number of episodes (95% UI)	..	..	..	..
**0–<60m†**				
Studies	2	2	2	4
Hospital admission rate (95% UI)	0·1 (<0·05, 4·9)	<0·05 (<0·05, 0·1)	0·1 (0·1, 0·3)	<0·05 (<0·05, 0·1)
Number of episodes (95% UI)	11 (0, 833)	6 (2, 24)	20 (10, 42)	5 (1, 19)
	**RSV-associated ALRI in-hospital CFR**			
**High-income Countries**				
**0–<12m**				
Studies	10	13		
In-hospital CFR (%)	0·1 (<0·05, 1·3)	<0·05 (<0·05, 2·2)		
**12–<60m**				
Studies	9	10		
In-hospital CFR (%)	0·3 (<0·05, 2·2)	0·1 (<0·05, 4·8)		
**0–<60m**				
Studies	9	13		
In-hospital CFR (%)	0·1 (<0·05, 0·5)	<0·05 (<0·05, 0·7)		
**Upper-middle-income Countries**				
**0–<12m**				
Studies	8	8		
In-hospital CFR (%)	0·4 (0·1, 1·0)	<0·05 (<0·05, 6·7)		
**0–<60m**				
Studies	8	9		
In-hospital CFR (%)	0·3 (0·1, 0·7)	<0·05 (<0·05, 6·3)		

Note that studies focusing on indigenous populations in industrialised countries were all from Alaska, US. These studies were reclassified as high-income/industrialised countries in this sensitivity analysis. Hospitalisation rates were shown in per 1000 children and number in thousands. UI = uncertainty interval. †The point estimates and uncertainty interval estimates are not necessarily equal to the sum of the estimates by finer age bands; this is because the studies that contributed to age-specific estimates were different. ‡For in-hospital CFR, 2020 time period means 2019 and 2020 onwards because no further stratification due to data scarcity. § Global estimates were obtained by summing the numbers of developing and industrialised countries for each of the 1000 samples in the Monte Carlo simulation.

Table S11. Median, IQR and range of optimal lag time between 12-month moving average RSV-associated ALRI hospitalisation rates and Retail & recreation index by World Bank income region

	High-income Countries		Upper-middle-income Countries		Lower-middle-income Countries	
**Age groups**	**Lag time (IQR)**	**Lag time (Range)**	**Lag time (IQR)**	**Lag time (Range)**	**Lag time (IQR)**	**Lag time (Range)**
**0–<3m**	0·0 (0·0, 0·0)	0·0 (0·0, 2·0)	0·0 (0·0, 3·5)	0·0 (0·0, 7·0)	—	—
**3–<6m**	0·0 (0·0, 1·5)	0·0 (0·0, 2·0)	1·5 (0·8, 2·2)	1·5 (0·0, 3·0)	—	—
**6–<9m**	0·0 (0·0, 1·0)	0·0 (0·0, 2·0)	2·0 (1·0, 2·5)	2·0 (0·0, 3·0)	—	—
**9–<12m**	0·0 (0·0, 1·5)	0·0 (0·0, 3·0)	3·0 (3·0, 3·0)	3·0 (3·0, 3·0)	—	—
**12–<24m**	0·0 (0·0, 0·8)	0·0 (0·0, 2·0)	4·0 (2·0, 4·0)	4·0 (0·0, 4·0)	0·0 (0·0, 0·0)	0·0 (0·0, 0·0)
**0–<6m**	0·0 (0·0, 0·0)	0·0 (0·0, 2·0)	0·0 (0·0, 2·5)	0·0 (0·0, 5·0)	—	—
**6–<12m**	0·0 (0·0, 1·5)	0·0 (0·0, 3·0)	0·0 (0·0, 1·5)	0·0 (0·0, 3·0)	—	—
**12–<60m**	0·0 (0·0, 1·5)	0·0 (0·0, 2·0)	3·5 (3·2, 3·8)	3·5 (3·0, 4·0)	1·0 (1·0, 1·0)	1·0 (1·0, 1·0)
**0–<60m**	0·0 (0·0, 0·8)	0·0 (0·0, 2·0)	2·0 (1·0, 3·0)	2·0 (0·0, 4·0)	—	—

m = months; lag time unit: months

Table S12. Odds ratio for observing RSV-associated ALRI hospitalisations in older age groups (compared with 0–<3 months) during the COVID-19 pandemic period (compared to the year 2019)

	2020 compared to 2019	2021 compared to 2019	Latest (April 2021 to March 2022) compared to 2019
	**RSV-associated ALRI hospitalisations**		
**High-income Countries**			
**3–<6m**			
Studies	8	8	7
OR (95% UI)	1.00 (0.78, 1.29)	1.19 (0.99, 1.42)	1.13 (0.94, 1.36)
**6–<9m**			
Studies	8	8	7
OR (95% UI)	0.81 (0.56, 1.18)	1.55 (1.23, 1.97)	1.18 (0.92, 1.50)
**9–<12m**			
Studies	8	8	7
OR (95% UI)	1.41 (0.99, 2.03)	1.48 (1.13, 1.95)	1.24 (0.94, 1.65)
**12–<18m**			
Studies	7	7	6
OR (95% UI)	1.35 (0.98, 1.86)	1.63 (1.29, 2.05)	1.36 (1.07, 1.73)
**18–<24m**			
Studies	7	7	6
OR (95% UI)	1.54 (1.09, 2.17)	1.96 (1.54, 2.51)	1.85 (1.44, 2.38)
**12–<24m**			
Studies	7	7	6
OR (95% UI)	1.35 (1.04, 1.76)	1.72 (1.42, 2.09)	1.54 (1.27, 1.87)
**Upper-middle-income Countries**			
**3–<6m**			
Studies	10	9	6
OR (95% UI)	0.90 (0.77, 1.05)	0.63 (0.51, 0.76)	0.70 (0.58, 0.85)
**6–<9m**			
Studies	10	9	6
OR (95% UI)	1.47 (1.19, 1.83)	2.03 (1.61, 2.56)	2.18 (1.73, 2.75)
**9–<12m**			
Studies	10	9	6
OR (95% UI)	0.76 (0.59, 0.98)	1.15 (0.87, 1.52)	1.26 (0.96, 1.65)
**12–<18m**			
Studies	10	9	6
OR (95% UI)	1.12 (0.88, 1.43)	1.48 (1.13, 1.93)	1.77 (1.37, 2.29)
**18–<24m**			
Studies	10	9	6
OR (95% UI)	1.55 (1.18, 2.04)	2.16 (1.59, 2.92)	2.53 (1.87, 3.43)
**12–<24m**			
Studies	10	9	6
OR (95% UI)	1.30 (1.07, 1.59)	1.74 (1.40, 2.17)	2.05 (1.66, 2.54)
**Lower-middle -income Countries**			
**3–<6m**			
Studies	1	1	0
OR (95% UI)	0.89 (0.46, 1.71)	0.80 (0.38, 1.68)	··
**6–<9m**			
Studies	1	1	0
OR (95% UI)	1.65 (0.76, 3.56)	2.05 (0.92, 4.57)	··
**9–<12m**			
Studies	1	1	0
OR (95% UI)	0.68 (0.26, 1.77)	1.15 (0.46, 2.87)	··
**12–<18m**			
Studies	1	1	0
OR (95% UI)	5.04 (1.66, 15.36)	7.17 (2.34, 21.99)	··
**18–<24m**			
Studies	1	1	0
OR (95% UI)	0.69 (0.23, 2.09)	1.28 (0.46, 3.54)	··
**12–<24m**			

	2020 compared to 2019	2021 compared to 2019	Latest (April 2021 to March 2022) compared to 2019
Studies	1	1	0
OR (95% UI)	2.04 (0.96, 4.35)	2.96 (1.37, 6.39)	··
**RSV-associated ALRI hospitalisations requiring supplemental oxygen (a subset of RSV-ALRI hospitalisations)**			
**High-income Countries**			
**3–<6m**			
Studies	4	4	4
OR (95% UI)	0.90 (0.34, 2.34)	1.28 (0.62, 2.63)	1.15 (0.57, 2.33)
**6–<9m**			
Studies	4	4	4
OR (95% UI)	0.27 (0.03, 2.21)	2.00 (0.79, 5.04)	1.77 (0.71, 4.41)
**9–<12m**			
Studies	4	4	4
OR (95% UI)	1.57 (0.42, 5.92)	2.39 (0.78, 7.28)	2.18 (0.73, 6.52)
**12–<18m**			
Studies	3	3	3
OR (95% UI)	2.83 (0.64,12.46)	4.60 (1.45,14.59)	4.96 (1.51,16.35)
**18–<24m**			
Studies	0	3	3
OR (95% UI)	··	2.29 (0.54, 9.72)	2.54 (0.56,11.40)
**12–<24m**			
Studies	3	3	3
OR (95% UI)	1.47 (0.39, 5.57)	2.82 (1.11, 7.15)	2.91 (1.13, 7.49)
**Upper-middle-income Countries**			
**3–<6m**			
Studies	7	7	5
OR (95% UI)	0.98 (0.69, 1.39)	0.89 (0.61, 1.30)	0.96 (0.66, 1.40)
**6–<9m**			
Studies	7	7	5
OR (95% UI)	1.37 (0.86, 2.19)	1.24 (0.77, 1.99)	1.52 (0.95, 2.41)
**9–<12m**			
Studies	7	7	5
OR (95% UI)	0.83 (0.44, 1.56)	1.18 (0.66, 2.08)	1.49 (0.86, 2.57)
**12–<18m**			
Studies	7	7	5
OR (95% UI)	0.89 (0.49, 1.61)	1.09 (0.60, 1.97)	1.31 (0.75, 2.29)
**18–<24m**			
Studies	7	7	5
OR (95% UI)	2.43 (1.13, 5.21)	1.51 (0.65, 3.52)	2.28 (1.01, 5.17)
**12–<24m**			
Studies	7	7	5
OR (95% UI)	1.35 (0.84, 2.18)	1.21 (0.73, 2.01)	1.55 (0.96, 2.51)
**Lower-middle -income Countries**			
**3–<6m**			
Studies	1	1	0
OR (95% UI)	2.25 (0.11,45.73)	2.25 (0.17,29.77)	··
**6–<9m**			
Studies	1	1	0
OR (95% UI)	2.25 (0.11,45.73)	1.12 (0.06,21.09)	··
**18–<24m**			
Studies	0	1	0
OR (95% UI)	··	3.38 (0.29,39.32)	··
**12–<24m**			
Studies	1	1	0
OR (95% UI)	1.50 (0.18,12.78)	1.12 (0.17, 7.24)	··
**RSV-associated ALRI hospitalisations requiring mechanical ventilation or ICU admission (a subset of RSV-ALRI hospitalisations)**			
**High-income Countries**			
**3–<6m**			
Studies	7	7	6

	2020 compared to 2019	2021 compared to 2019	Latest (April 2021 to March 2022) compared to 2019
OR (95% UI)	0.59 (0.35, 0.97)	1.22 (0.93, 1.60)	1.16 (0.88, 1.54)
**6–<9m**			
Studies	7	7	6
OR (95% UI)	0.89 (0.39, 2.03)	1.70 (1.08, 2.69)	1.48 (0.92, 2.37)
**9–<12m**			
Studies	7	7	6
OR (95% UI)	1.52 (0.63, 3.65)	2.05 (1.16, 3.63)	1.63 (0.90, 2.95)
**12–<18m**			
Studies	6	6	5
OR (95% UI)	1.12 (0.53, 2.38)	1.29 (0.79, 2.10)	0.99 (0.59, 1.66)
**18–<24m**			
Studies	6	6	5
OR (95% UI)	1.86 (0.82, 4.23)	2.64 (1.50, 4.66)	2.52 (1.43, 4.45)
**12–<24m**			
Studies	6	6	5
OR (95% UI)	1.40 (0.79, 2.48)	1.74 (1.18, 2.55)	1.50 (1.01, 2.24)
**Upper-middle-income Countries**			
**3–<6m**			
Studies	5	5	0
OR (95% UI)	0.71 (0.18, 2.78)	0.88 (0.22, 3.58)	··
**6–<9m**			
Studies	4	3	0
OR (95% UI)	1.46 (0.24, 8.85)	0.42 (0.04, 4.88)	··
**18–<24m**			
Studies	4	0	0
OR (95% UI)	0.56 (0.03, 10.93)	··	··
**12–<24m**			
Studies	4	0	0
OR (95% UI)	0.28 (0.02, 3.88)	··	··
**Lower-middle -income Countries**			
**3–<6m**			
Studies	1	1	0
OR (95% UI)	1.33 (0.06, 31.12)	2.00 (0.13, 31.98)	··
**6–<9m**			
Studies	0	1	0
OR (95% UI)	··	1.00 (0.05, 22.18)	··

RSV = respiratory syncytial virus; ALRI = acute lower respiratory infection; OR = odds ratio. UI = uncertainty interval.

Table S13. Comparison of proportion of RSV-associated ALRI hospitalisations that needed supplemental oxygen and required mechanical ventilation or ICU admission between pre-pandemic and pandemic period, by broader age group and World Bank Income Region.

	2020 compared to 2019	2021 compared to 2019	Latest (April 2021 to March 2022) compared to 2019
**RSV-associated ALRI hospitalisations that needed supplemental oxygen (a subset of RSV-ALRI hospitalisations)**			
**High-income Countries**			
**0–<6m**			
Studies	4	4	4
OR (95% UI)	0.13 (0.05, 0.35)	1.24 (0.65, 2.35)	0.45 (0.25, 0.81)
**6–<12m**			
Studies	4	4	4
OR (95% UI)	0.29 (0.08, 1.07)	0.78 (0.32, 1.86)	0.61 (0.26, 1.45)
**12–<60m**			
Studies	3	3	3
OR (95% UI)	1.58 (0.51, 4.87)	2.50 (1.16, 5.40)	3.14 (1.45, 6.79)
**0–<24m**			
Studies	3	3	3
OR (95% UI)	1.03 (0.53, 2.00)	1.37 (0.89, 2.11)	1.43 (0.92, 2.21)
**0–<60m**			
Studies	3	3	3
OR (95% UI)	1.30 (0.72, 2.37)	1.51 (1.01, 2.26)	1.67 (1.11, 2.51)
**Upper-middle-income Countries**			
**0–<6m**			
Studies	7	7	5
OR (95% UI)	1.73 (1.40, 2.13)	1.73 (1.34, 2.23)	1.50 (1.16, 1.94)
**6–<12m**			
Studies	7	7	5
OR (95% UI)	1.74 (1.10, 2.74)	1.48 (0.92, 2.37)	1.38 (0.87, 2.18)
**12–<60m**			
Studies	7	7	5
OR (95% UI)	1.46 (0.98, 2.19)	0.90 (0.57, 1.42)	1.12 (0.74, 1.72)
**0–<24m**			
Studies	7	7	5
OR (95% UI)	1.46 (1.22, 1.75)	1.27 (1.04, 1.57)	1.15 (0.94, 1.40)
**0–<60m**			
Studies	7	7	5
OR (95% UI)	1.48 (1.24, 1.75)	1.17 (0.96, 1.43)	1.10 (0.91, 1.34)
**Lower-middle -income Countries**			
**0–<6m**			
Studies	1	1	0
OR (95% UI)	0.67 (0.21, 2.19)	1.98 (0.75, 5.25)	‥
**6–<12m**			
Studies	1	1	0
OR (95% UI)	1.78 (0.11,29.86)	6.15 (0.60,62.93)	‥
**12–<60m**			
Studies	1	1	0
OR (95% UI)	0.37 (0.07, 2.07)	0.54 (0.14, 2.00)	‥
**0–<24m**			
Studies	1	1	0
OR (95% UI)	1.16 (0.44, 3.10)	2.96 (1.28, 6.84)	‥
**0–<60m**			
Studies	1	1	0
OR (95% UI)	0.83 (0.33, 2.11)	2.42 (1.15, 5.08)	‥
**RSV-associated ALRI hospitalisations requiring mechanical ventilation or ICU admission (a subset of RSV-ALRI hospitalisations)**			
**High-income Countries**			
**0–<6m**			
Studies	7	7	6
OR (95% UI)	1.34 (0.88, 2.05)	1.22 (0.93, 1.60)	1.17 (0.89, 1.54)

	2020 compared to 2019	2021 compared to 2019	Latest (April 2021 to March 2022) compared to 2019
**6–<12m**			
Studies	7	7	6
OR (95% UI)	1.13 (0.39, 3.30)	1.43 (0.70, 2.92)	1.14 (0.53, 2.43)
**12–<60m**			
Studies	5	5	4
OR (95% UI)	1.72 (0.66, 4.54)	0.92 (0.53, 1.59)	0.65 (0.37, 1.13)
**0–<24m**			
Studies	31	6	5
OR (95% UI)	1.24 (1.02, 1.50)	1.13 (0.90, 1.43)	1.06 (0.84, 1.34)
**0–<60m**			
Studies	5	5	4
OR (95% UI)	1.29 (0.89, 1.89)	0.86 (0.68, 1.10)	0.77 (0.60, 0.99)
**Upper-middle-income Countries**			
**0–<6m**			
Studies	7	7	5
OR (95% UI)	1.80 (0.92, 3.53)	1.20 (0.58, 2.48)	0.84 (0.27, 2.58)
**6–<12m**			
Studies	8	8	0
OR (95% UI)	1.97 (0.46, 8.46)	0.12 (0.01, 1.12)	··
**12–<60m**			
Studies	8	8	0
OR (95% UI)	1.87 (0.57, 6.20)	0.43 (0.04, 5.14)	··
**0–<24m**			
Studies	7	7	5
OR (95% UI)	1.92 (1.07, 3.47)	0.78 (0.39, 1.53)	0.55 (0.19, 1.58)
**0–<60m**			
Studies	7	7	5
OR (95% UI)	1.71 (1.01, 2.91)	0.76 (0.39, 1.50)	0.55 (0.19, 1.57)
**Lower-middle -income Countries**			
**0–<6m**			
Studies	1	1	0
OR (95% UI)	1.43 (0.39, 5.28)	2.41 (0.69, 8.36)	··
**6–<12m**			
Studies	0	1	0
OR (95% UI)	··	3.90 (0.33, 45.59)	··
**12–<60m**			
Studies	0	1	0
OR (95% UI)	··	0.70 (0.13, 3.76)	··
**0–<24m**			
Studies	1	1	0
OR (95% UI)	1.14 (0.36, 3.58)	2.19 (0.79, 6.05)	··
**0–<60m**			
Studies	1	1	0
OR (95% UI)	0.81 (0.27, 2.41)	1.63 (0.65, 4.07)	··

RSV = respiratory syncytial virus; ALRI = acute lower respiratory infection; OR = odds ratio. UI = uncertainty interval.

Supplementary figures

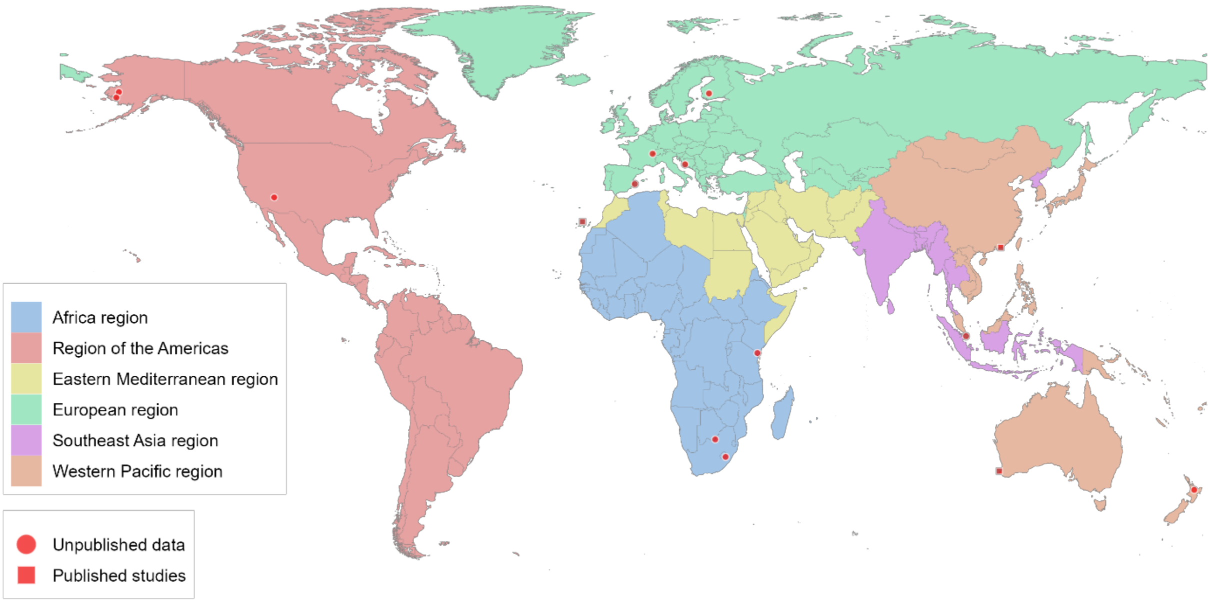

Figure S1. Geographical distribution of included studies reporting RSV-associated ALRI hospitalisation rates.

RSV = respiratory syncytial virus, ALRI = acute lower respiratory infection.

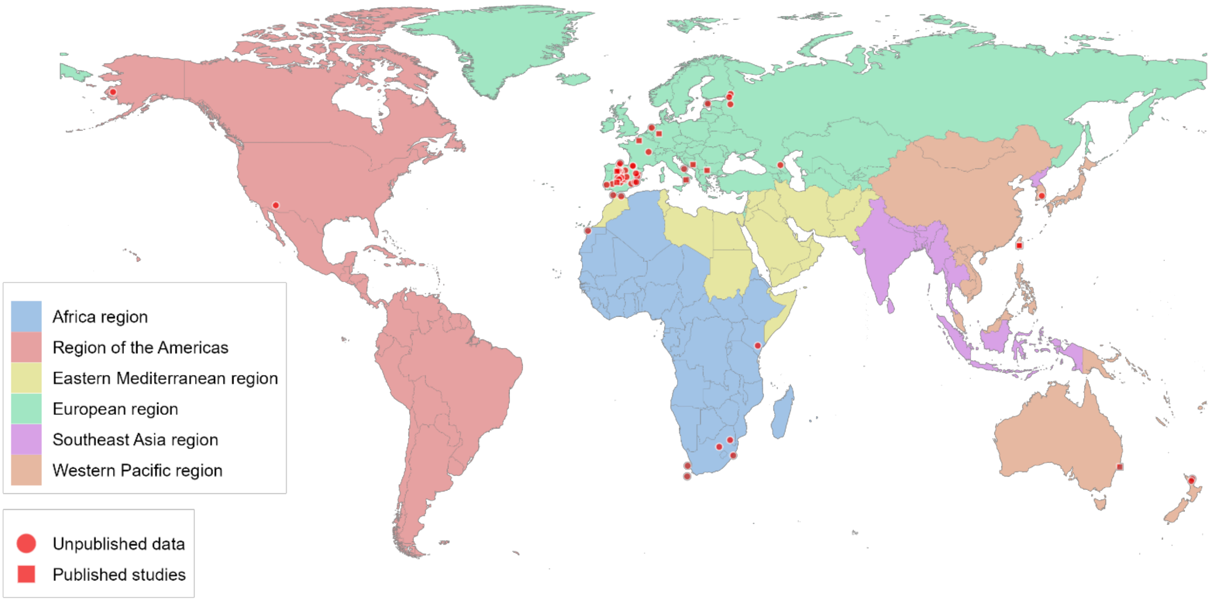

Figure S2. Geographical distribution of included studies reporting proportion of severe outcomes or in-hospital CFR among hospitalised RSV-ALRI cases.

Note that 25 sites in Spain only reported the proportion of requiring intensive care unit admission among RSV-ALRI in children aged 0–<24 months (retrospective multicentric national study using data obtained from the Pediatric Spanish Society).[2] Severe outcomes = hospitalised ALRI that needed supplemental oxygen or that needed mechanical ventilation or ICU admission. RSV = respiratory syncytial virus, ALRI = acute lower respiratory infection, CFR = case fatality ratio.

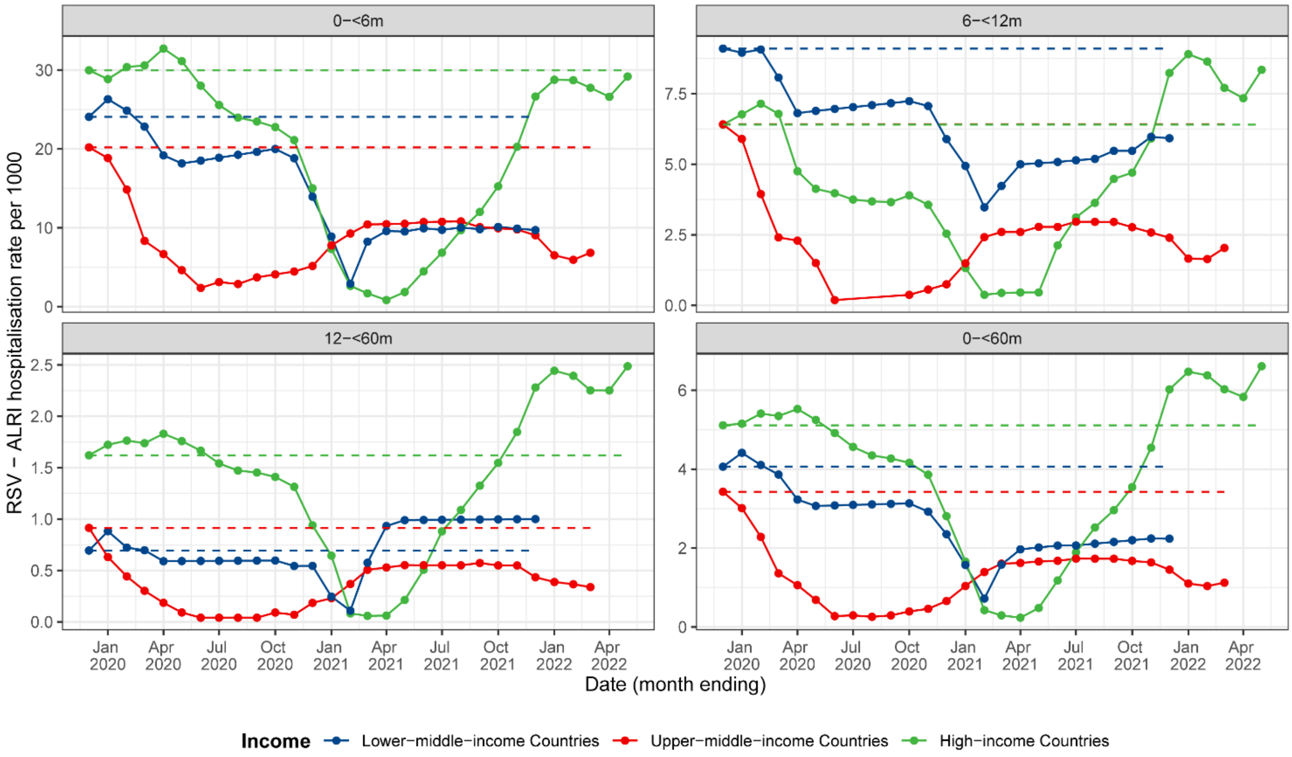

Figure S3. Change in the 12-month retrospective hospitalisation rates of RSV-associated ALRI by broader age group and World Bank income region.

The moving average curve was plotted based on studies (5, 2 and 1 from high-income, upper-middle-income and lower-middle-income countries, respectively) reporting complete data (i.e., RSV-ALRI hospitalisation rate from January 2019 to March 2022 or later). Note that on the X-axis, date represents the end of the 12-month interval — e.g., Jan 2020 represents the time period between February 2019 and January 2020. The dotted lines represent the corresponding RSV-associated ALRI hospitalisation rates in the pre-pandemic reference period (i.e., the year 2019). RSV = respiratory syncytial virus; ALRI = acute lower respiratory infection.

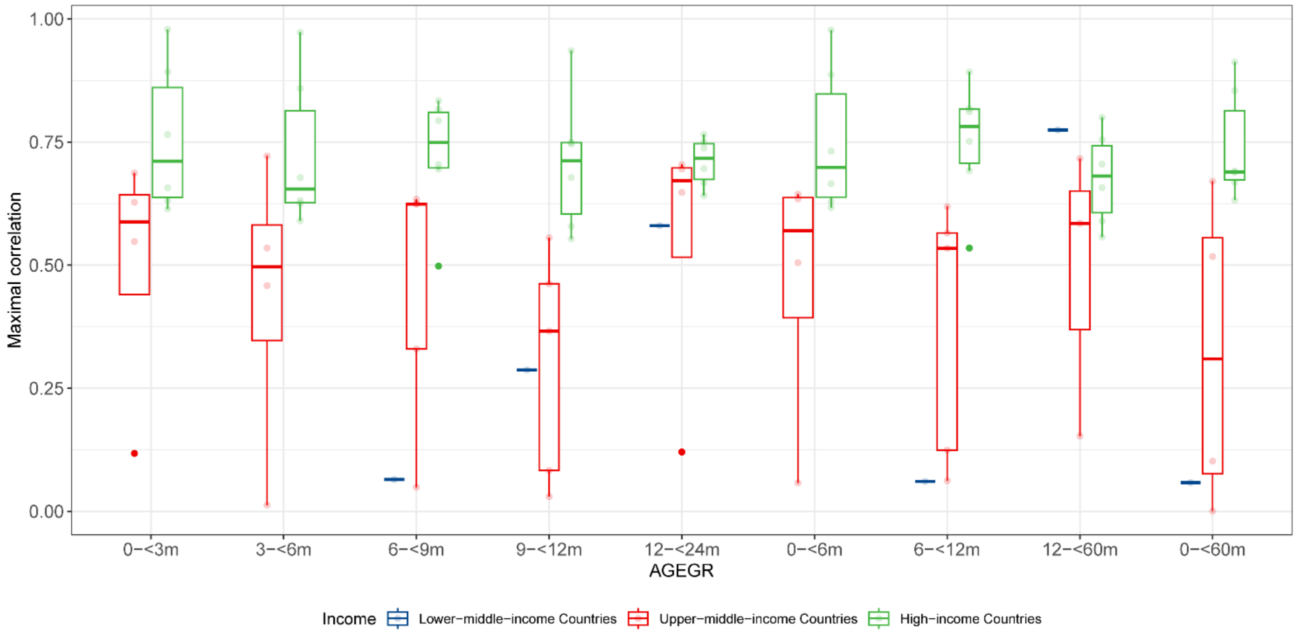

Figure S4. Maximal correlation coefficient of 12-month moving average RSV-associated ALRI hospitalisation rates and Retail & recreation index by age group and World Bank income region.

Top of box represents upper IQR of maximum correlation coefficient between the 12-month moving average population mobility, middle of box represents median IQR maximum correlation coefficient, and bottom of box represents lower IQR of maximum correlation coefficient. Top end of whisker represents highest value, excluding outliers (defined as any points that have a longer distance than 1.5 times IQR from the box). Bottom end of whisker represents lowest value, excluding outliers. Dots represent outliers. Pearson's correlation was used to compute the cross-correlation between two time series. Retail & recreation index (an index of Google COVID-19 Community Mobility Trends), its change was relative to baseline days before the pandemic outbreak (the median value over the five-week period from January 3 to February 6, 2020).

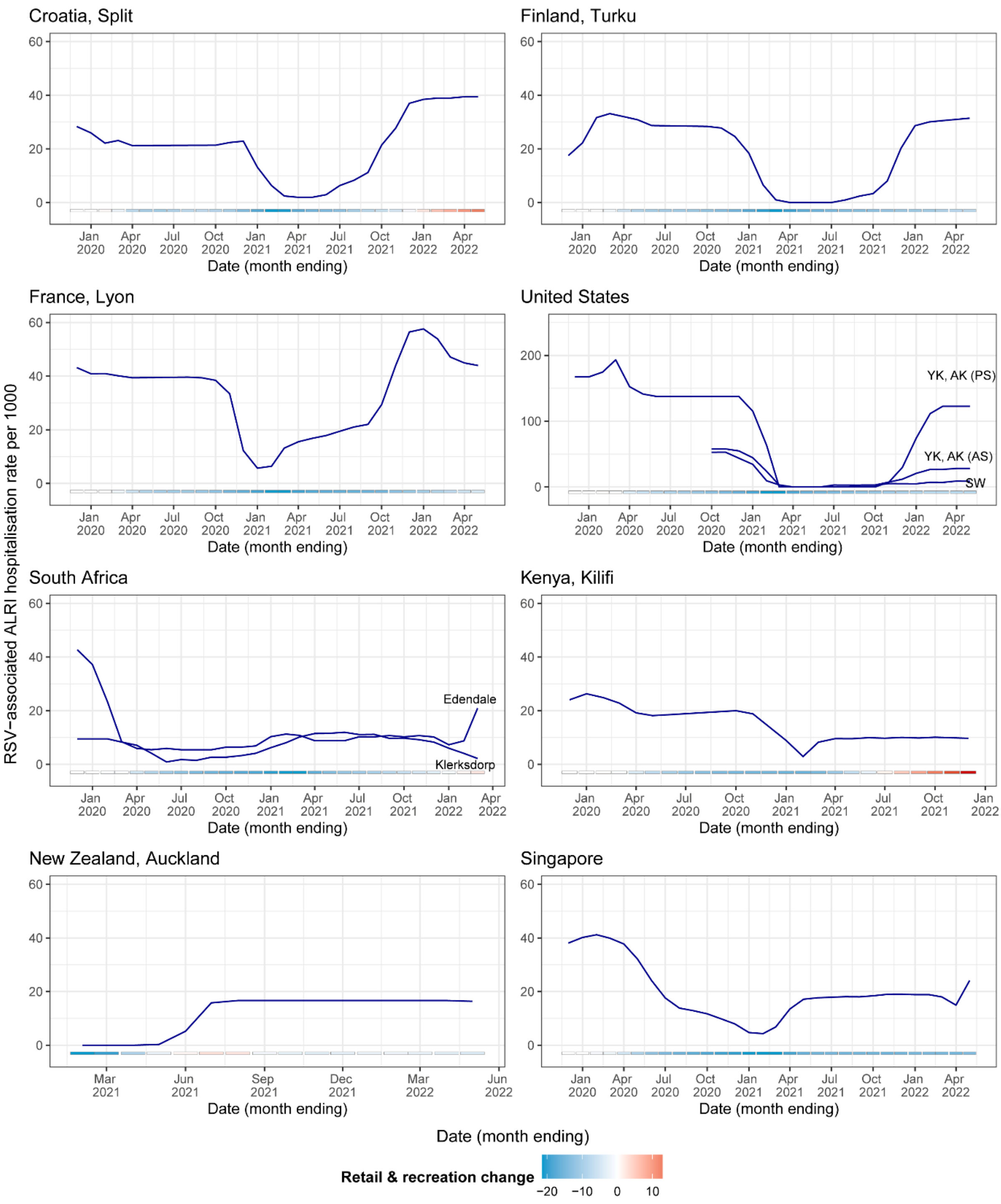

Figure S5. Changes over time of 12-month moving average RSV-associated ALRI hospitalisation rate in infants less than 6 months and Retail & recreation index by study site.

Retail & recreation index (an index of Google COVID-19 Community Mobility Trends), its change was relative to baseline days before the pandemic outbreak (the median value over the five-week period from January 3 to February 6, 2020). YK, AK (PS) = Yukon Kuskokwim, Alaska (passive surveillance); YK, AK (AS) = Yukon Kuskokwim, Alaska (active surveillance); SW = Southwest United States; RSV = respiratory syncytial virus; ALRI = acute lower respiratory infection.

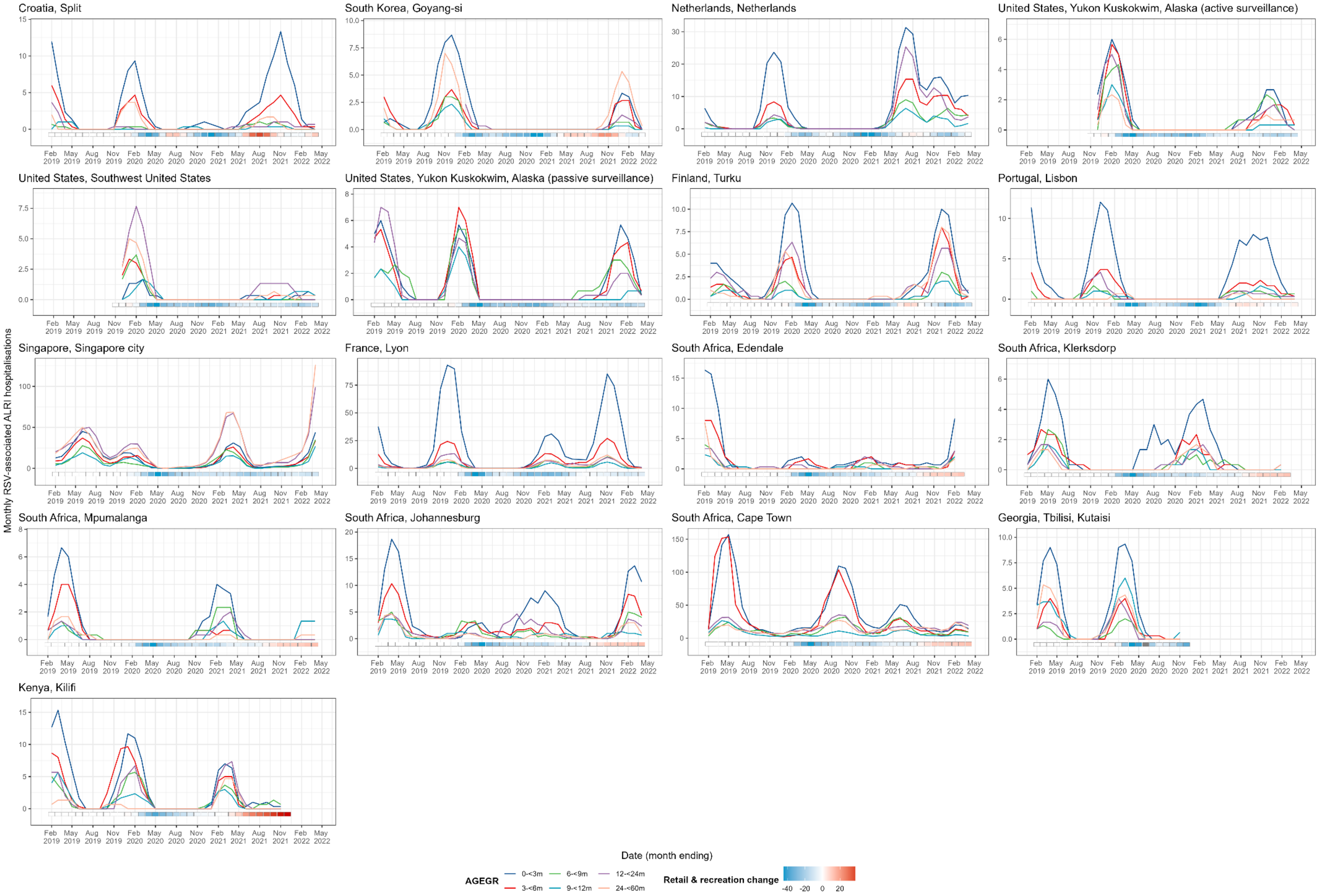
Croatia, Split
South Korea, Goyang-si
Netherlands, Netherlands
United States, Yukon Kuskokwim, Alaska (active surveillance)
United States, Southwest United States
United States, Yukon Kuskokwim, Alaska (passive surveillance)
Finland, Turku
Portugal, Lisbon
Singapore, Singapore city
France, Lyon
South Africa, Edendale
South Africa, Klerksdorp
South Africa, Mpumalanga
South Africa, Johannesburg
South Africa, Cape Town
Georgia, Tbilisi, Kutaisi
Kenya, Kilifi
Monthly RSV-associated ALRI hospitalisations
Date (month ending)
AGEGR
0-<3m
3-<6m
6-<9m
9-<12m
12-<24m
24-<60m
Retail & recreation change
-40
-20
0
20

Figure S6. Three-month moving average of monthly RSV-associated ALRI hospitalisations and Retail & recreation index for children young than five years, stratified by age group and study site.

Retail & recreation index (an index of Google COVID-19 Community Mobility Trends), its change was relative to baseline days before the pandemic outbreak (the median value over the five-week period from January 3 to February 6, 2020). The scale of the y-axis is drawn according to the minimum and maximum monthly hospitalisations for each sub-figure to visualise trends. The data is only presented in sub-figures for study sites with year-round testing (i.e., data from Spain and New Zealand were not presented as testing was not done during the perceived epidemic months). RSV = respiratory syncytial virus; ALRI = acute lower respiratory infection.

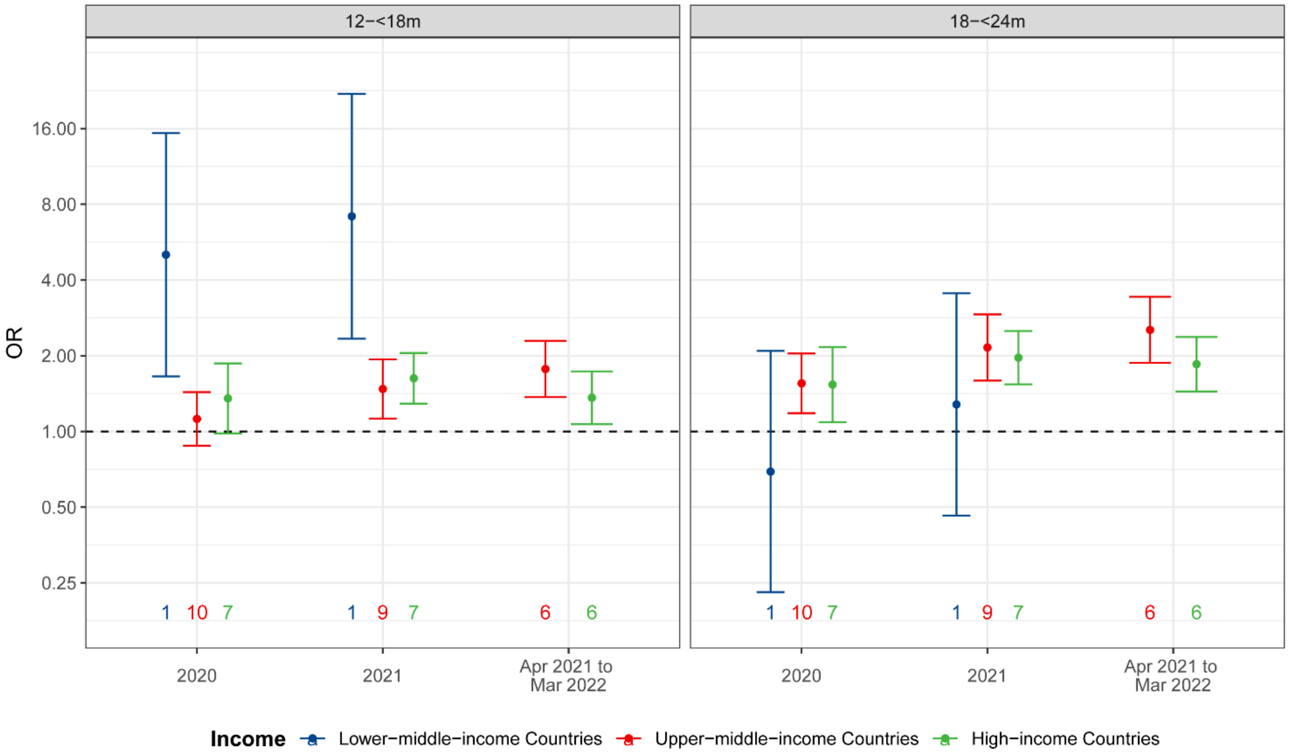

Figure S7. Odds ratio for observing RSV-associated ALRI hospitalisations in children aged 12–<18 months and 18–<24 months (compared with 0–<3 months) during the COVID-19 pandemic period (compared to the year 2019).

Dots and error bars indicate the point estimates and the corresponding 95% UIs. Number at the bottom of each panel indicates the number of data-points contributing to each group. OR = odds ratio. This data is available in the appendix pp 33–34.

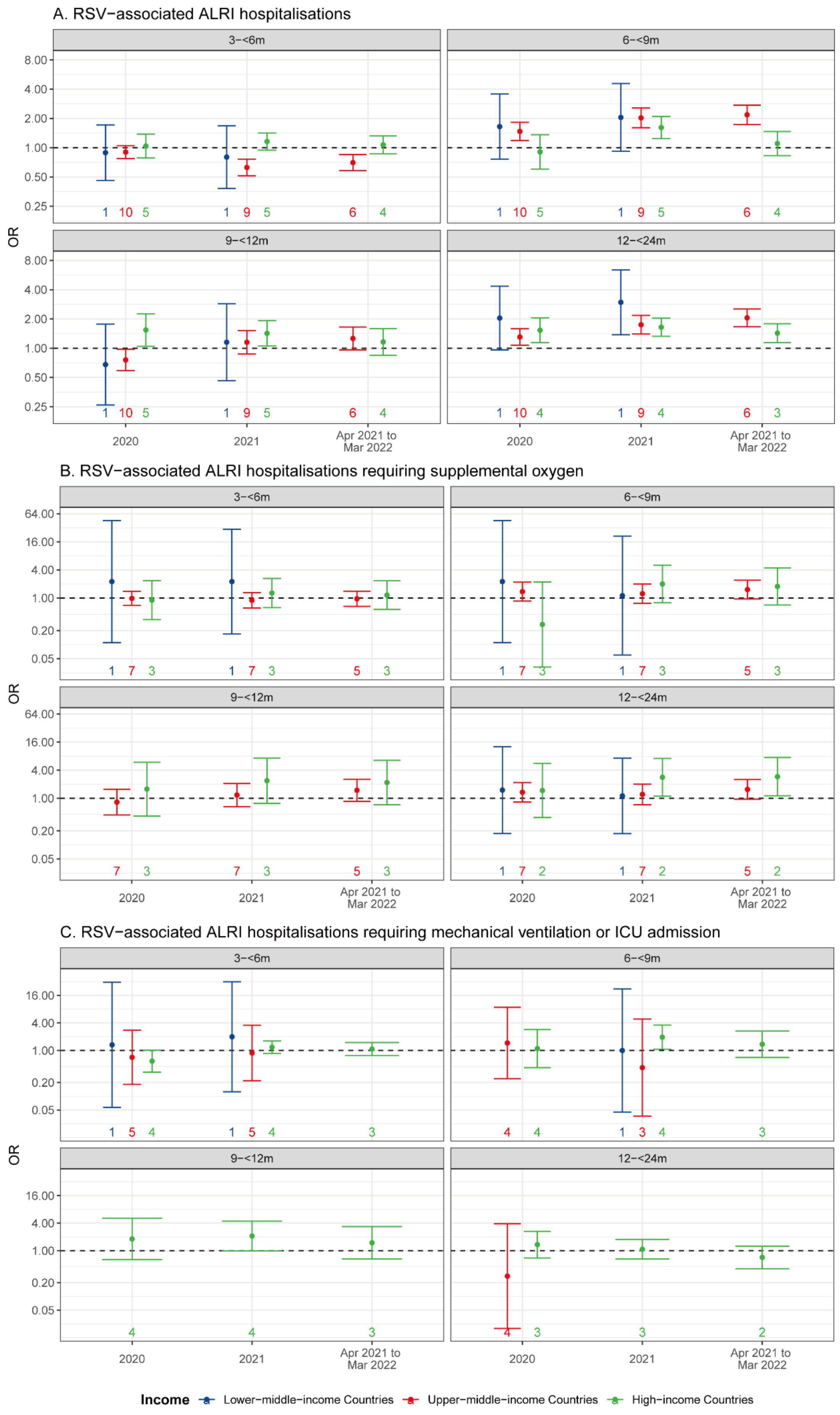
A. RSV−associated ALRI hospitalisations
B. RSV−associated ALRI hospitalisations requiring supplemental oxygen
C. RSV−associated ALRI hospitalisations requiring mechanical ventilation or ICU admission
3−<6m
6−<9m
9−<12m
12−<24m
OR
2020
2021
Apr 2021 to Mar 2022
Income
Lower−middle−income Countries
Upper−middle−income Countries
High−income Countries

Figure S8. Odds ratio for observing RSV-associated ALRI hospitalisations in older age groups (compared with 0–<3 months) during the COVID-19 pandemic period (compared to the year 2019), when only including studies with quality scores ≥0.6.

(A) RSV-associated ALRI hospitalisations; (B) RSV-associated ALRI hospitalisations requiring supplemental oxygen (a subset of ALRI hospitalisations); (C) RSV-associated ALRI hospitalisations requiring mechanical ventilation or ICU admission (a subset of ALRI hospitalisations). Dots and error bars indicate the point estimates and the corresponding 95% UIs. Number at the bottom of each panel indicates the number of data-points contributing to each group. OR = odds ratio. ICU = intensive care unit.

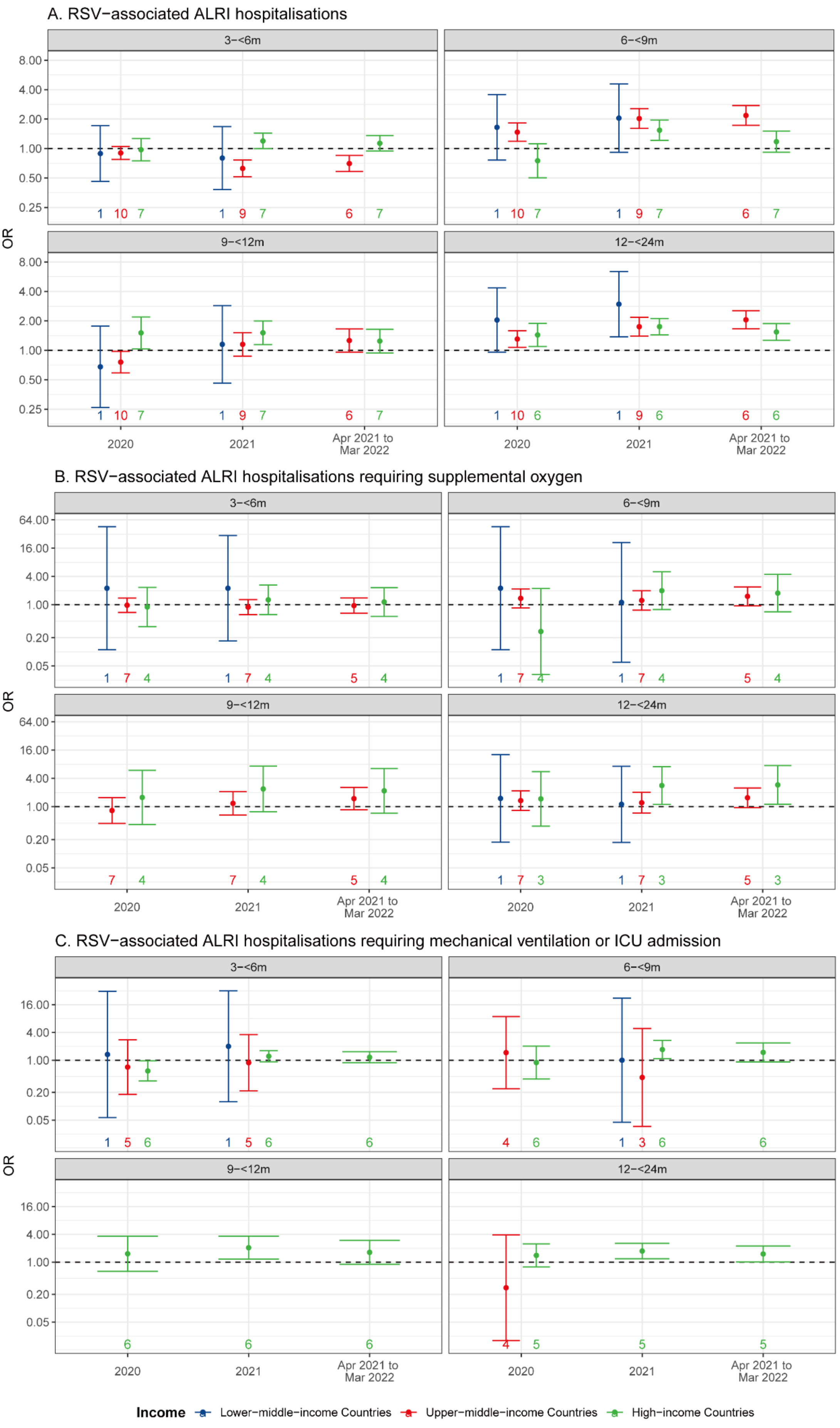
A. RSV−associated ALRI hospitalisations
B. RSV−associated ALRI hospitalisations requiring supplemental oxygen
C. RSV−associated ALRI hospitalisations requiring mechanical ventilation or ICU admission
3−<6m
6−<9m
9−<12m
12−<24m
OR
2020
2021
Apr 2021 to Mar 2022
Income
Lower−middle−income Countries
Upper−middle−income Countries
High−income Countries

Figure S9. Odds ratio for observing RSV-associated ALRI hospitalisations in older age groups (compared with 0–<3 months) during the COVID-19 pandemic period (compared to the year 2019), when only including studies with year-round testing.

(A) RSV-associated ALRI hospitalisations; (B) RSV-associated ALRI hospitalisations requiring supplemental oxygen (a subset of ALRI hospitalisations); (C) RSV-associated ALRI hospitalisations requiring mechanical ventilation or ICU admission (a subset of ALRI hospitalisations). Dots and error bars indicate the point estimates and the corresponding 95% UIs. Number at the bottom of each panel indicates the number of data-points contributing to each group. OR = odds ratio. ICU = intensive care unit.

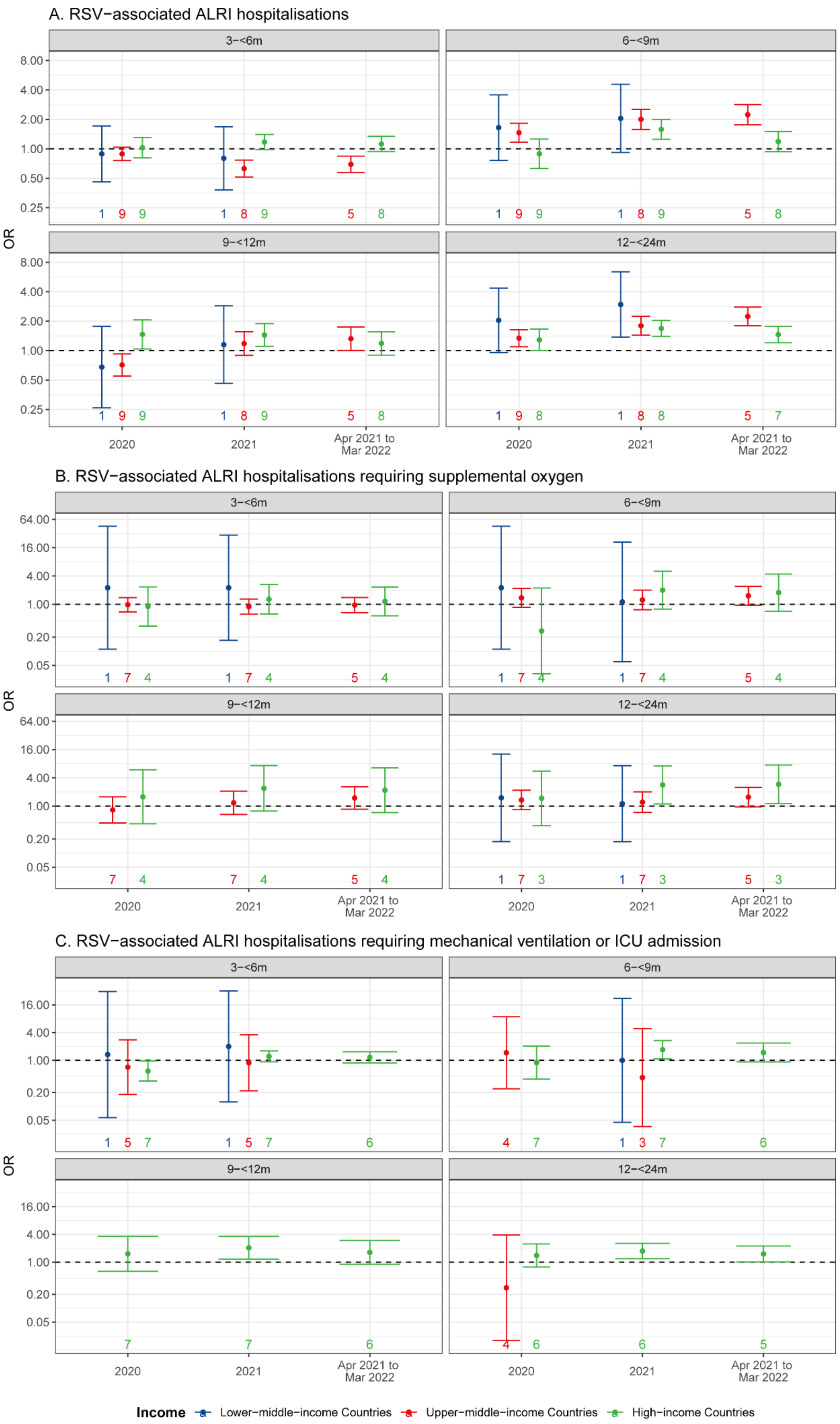
A. RSV−associated ALRI hospitalisations
B. RSV−associated ALRI hospitalisations requiring supplemental oxygen
C. RSV−associated ALRI hospitalisations requiring mechanical ventilation or ICU admission
3−<6m
6−<9m
9−<12m
12−<24m
OR
2020
2021
Apr 2021 to Mar 2022
Income
Lower−middle−income Countries
Upper−middle−income Countries
High−income Countries

Figure S10. Odds ratio for observing RSV-associated ALRI hospitalisations in older age groups (compared with 0–<3 months) during the COVID-19 pandemic period (compared to the year 2019), when reclassifying studies focusing on indigenous population.

(A) RSV-associated ALRI hospitalisations; (B) RSV-associated ALRI hospitalisations requiring supplemental oxygen (a subset of ALRI hospitalisations); (C) RSV-associated ALRI hospitalisations requiring mechanical ventilation or ICU admission (a subset of ALRI hospitalisations). Dots and error bars indicate the point estimates and the corresponding 95% UIs. Number at the bottom of each panel indicates the number of data-points contributing to each group. OR = odds ratio. ICU = intensive care unit.

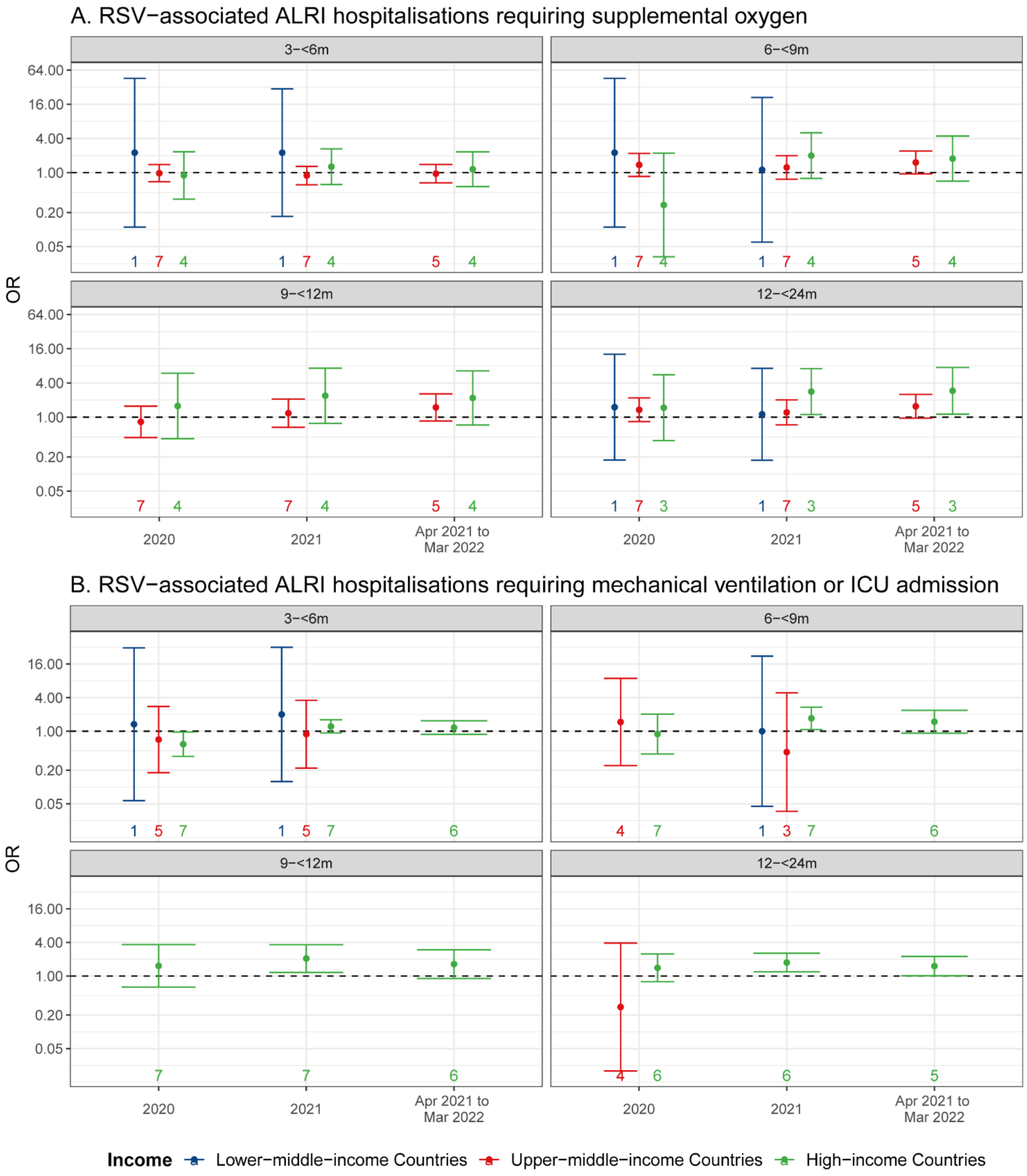

Figure S11. Odds ratio for observing RSV-associated ALRI hospitalisations in older age groups (compared with 0–<3 months) during the COVID-19 pandemic period (compared to the year 2019).

(A) RSV-associated ALRI hospitalisations requiring supplemental oxygen; (B) RSV-associated ALRI hospitalisations requiring mechanical ventilation or ICU admission (both are subsets of RSV-ALRI hospitalisations). Dots and error bars indicate the point estimates and the corresponding 95% UIs. Number at the bottom of each panel indicates the number of data-points contributing to each group. OR = odds ratio.

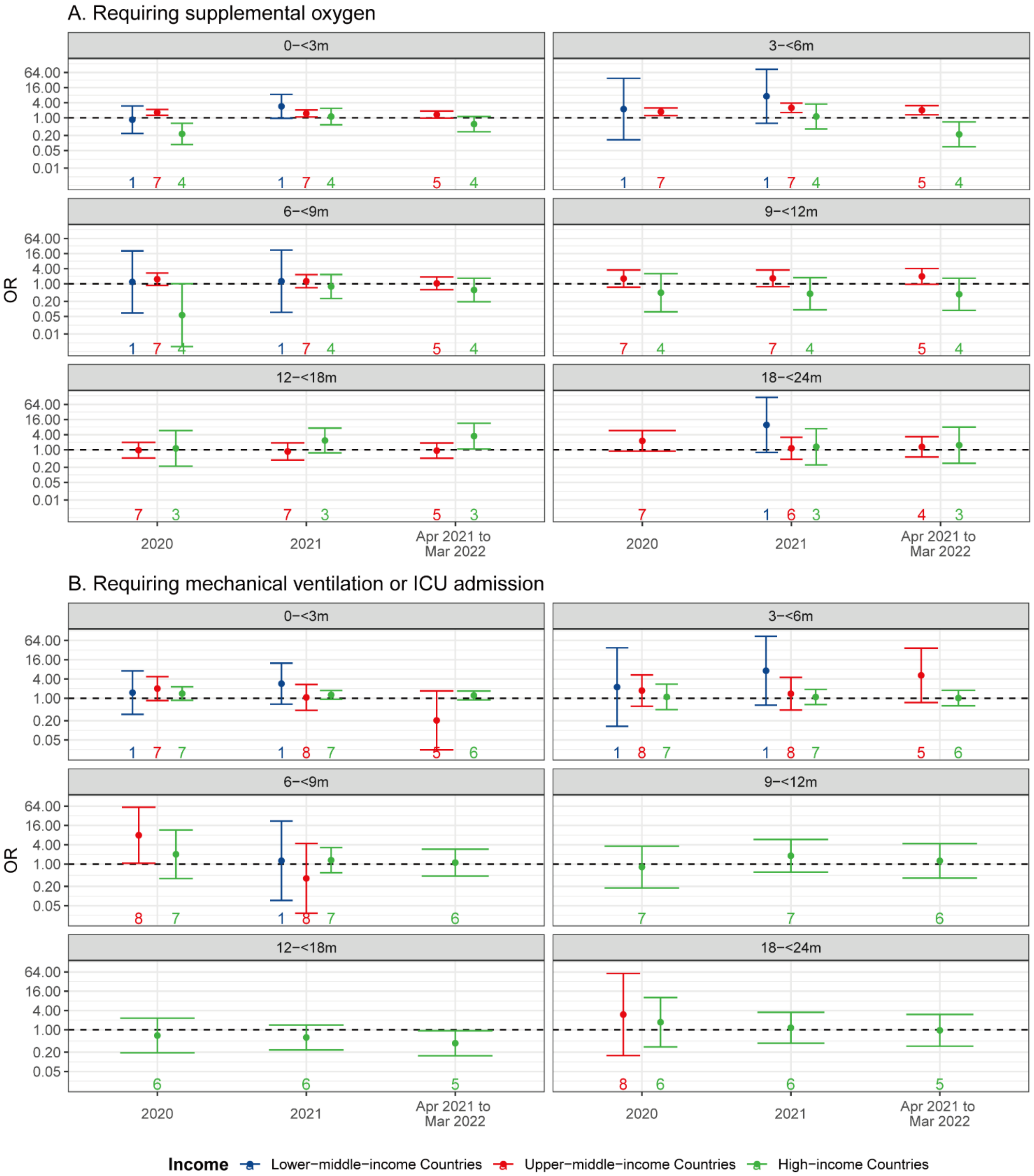

Figure S12. Comparison of proportion of RSV-associated ALRI hospitalisations that needed supplemental oxygen and required mechanical ventilation or ICU admission between pre-pandemic and pandemic period, by World Bank Income Region and finer age group.

(A) RSV-associated ALRI hospitalisations that needed supplemental oxygen; (B) RSV-associated ALRI hospitalisations requiring mechanical ventilation or ICU admission (both are subsets of RSV-ALRI hospitalisations). The pre-pandemic period denotes the year of 2019. Dots and error bars indicate the point estimates and the corresponding 95% UIs. Number at the bottom of each panel indicates the number of data-points contributing to each group. RSV = respiratory syncytial virus; ALRI = acute lower respiratory infection; OR = odds ratio; ICU = intensive care unit.

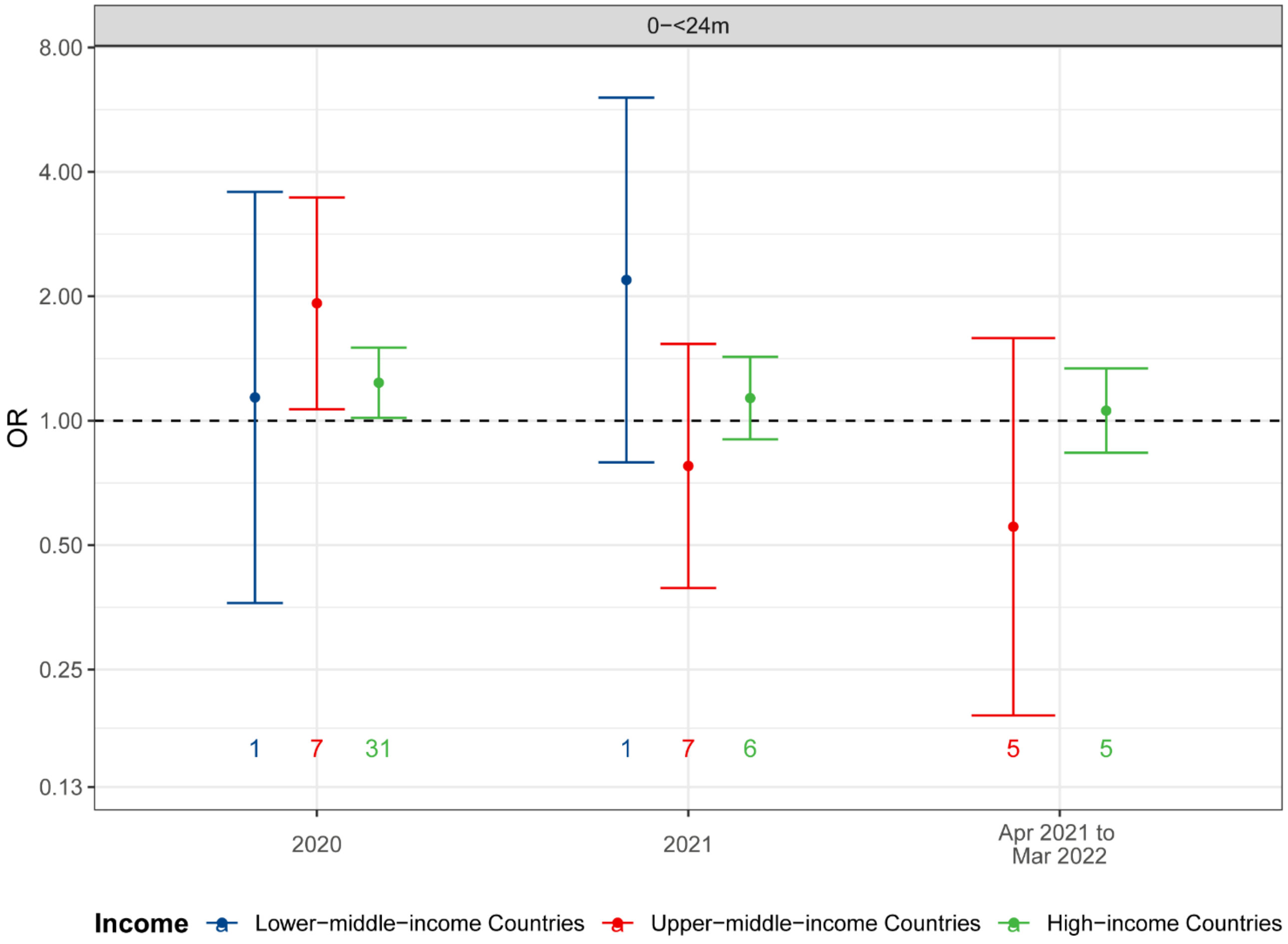

Figure S13. Comparison of proportion of RSV-associated ALRI hospitalisations requiring mechanical ventilation or ICU admission in children aged 0–<24 months between pre-pandemic and pandemic period, by World Bank Income Region.

Dots and error bars indicate the point estimates and the corresponding 95% UIs. The pre-pandemic period denotes the year of 2019. Number at the bottom of each panel indicates the number of data-points contributing to each group. OR = odds ratio. ICU = intensive care unit. In 2020, studies included were based on 32 sites (25 from Spain).[2]

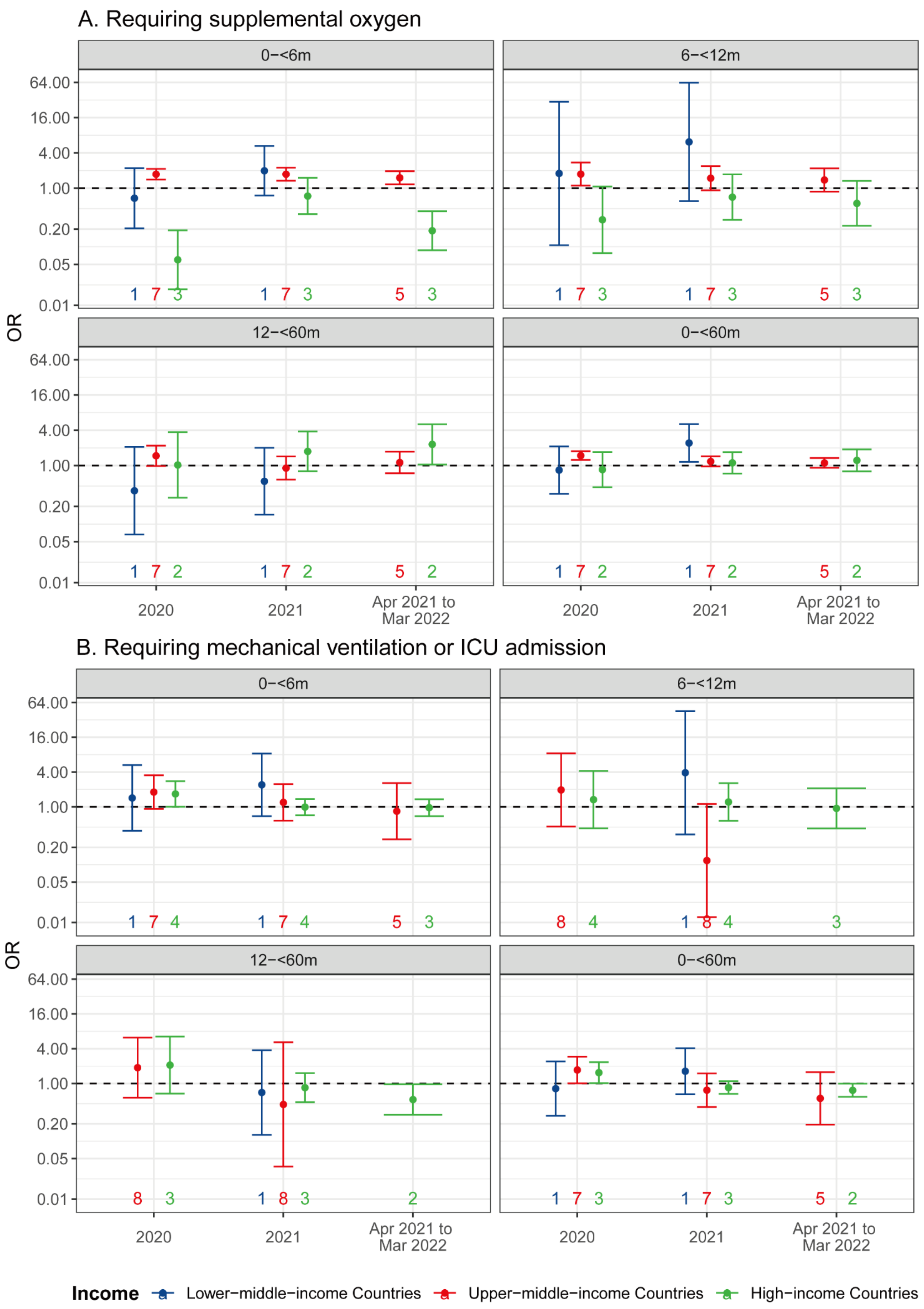
A. Requiring supplemental oxygen
0−<6m
6−<12m
12−<60m
0−<60m
B. Requiring mechanical ventilation or ICU admission
OR
64.00
16.00
4.00
1.00
0.20
0.05
0.01
2020
2021
Apr 2021 to Mar 2022
Income
Lower−middle−income Countries
Upper−middle−income Countries
High−income Countries

Figure S14. Comparison of proportion of RSV-associated ALRI hospitalisations that needed supplemental oxygen and required mechanical ventilation or ICU admission between pre-pandemic and pandemic period, by World Bank Income Region and age group, when only including studies with quality scores ≥0.6.

(A) RSV-associated ALRI hospitalisations that needed supplemental oxygen; (B) RSV-associated ALRI hospitalisations requiring mechanical ventilation or ICU admission (both are subsets of RSV-ALRI hospitalisations). Dots and error bars indicate the point estimates and the corresponding 95% UIs. The pre-pandemic period denotes the year of 2019. Number at the bottom of each panel indicates the number of data-points contributing to each group. OR = odds ratio. ICU = intensive care unit.

GATHER checklist

Checklist of information that should be included in new reports of global health estimates

Item #	Checklist item	Reported on page #
**Objectives and funding**		
**1**	Define the indicator(s), populations (including age, sex, and geographic entities), and time period(s) for which estimates were made.	5-6
**2**	List the funding sources for the work.	3
**Data Inputs**		
For all data inputs from multiple sources that are synthesized as part of the study:		
**3**	Describe how the data were identified and how the data were accessed.	5-8
**4**	Specify the inclusion and exclusion criteria. Identify all ad-hoc exclusions.	6
**5**	Provide information on all included data sources and their main characteristics. For each data source used, report reference information or contact name/institution, population represented, data collection method, year(s) of data collection, sex and age range, diagnostic criteria or measurement method, and sample size, as relevant.	5-8
**6**	Identify and describe any categories of input data that have potentially important biases (e.g., based on characteristics listed in item 5).	5-8
For data inputs that contribute to the analysis but were not synthesized as part of the study:		
**7**	Describe and give sources for any other data inputs.	NA
For all data inputs:		
**8**	Provide all data inputs in a file format from which data can be efficiently extracted (e.g., a spreadsheet rather than a PDF), including all relevant meta-data listed in item 5. For any data inputs that cannot be shared because of ethical or legal reasons, such as third-party ownership, provide a contact name or the name of the institution that retains the right to the data.	NA
**Data analysis**		
**9**	Provide a conceptual overview of the data analysis method. A diagram may be helpful.	7-8
**10**	Provide a detailed description of all steps of the analysis, including mathematical formulae. This description should cover, as relevant, data cleaning, data pre-processing, data adjustments and weighting of data sources, and mathematical or statistical model(s).	7-8
**11**	Describe how candidate models were evaluated and how the final model(s) were selected.	NA
**12**	Provide the results of an evaluation of model performance, if done, as well as the results of any relevant sensitivity analysis.	7-8
**13**	Describe methods for calculating uncertainty of the estimates. State which sources of uncertainty were, and were not, accounted for in the uncertainty analysis.	8
**14**	State how analytic or statistical source code used to generate estimates can be accessed.	15
**Results and Discussion**		
**15**	Provide published estimates in a file format from which data can be efficiently extracted.	9-11, 20-29
**16**	Report a quantitative measure of the uncertainty of the estimates (e.g., uncertainty intervals).	9-11, 20-29
**17**	Interpret results in light of existing evidence. If updating a previous set of estimates, describe the reasons for changes in estimates.	11-14
**18**	Discuss limitations of the estimates. Include a discussion of any modelling assumptions or data limitations that affect interpretation of the estimates.	13

PRISMA 2020 Checklist

Section and Topic	Item #	Checklist item	Location where item is reported
TITLE			
Title	1	Identify the report as a systematic review.	1
ABSTRACT			
Abstract	2	See the PRISMA 2020 for Abstracts checklist.	2
INTRODUCTION			
Rationale	3	Describe the rationale for the review in the context of existing knowledge.	4-5
Objectives	4	Provide an explicit statement of the objective(s) or question(s) the review addresses.	5
METHODS			
Eligibility criteria	5	Specify the inclusion and exclusion criteria for the review and how studies were grouped for the syntheses.	6
Information sources	6	Specify all databases, registers, websites, organisations, reference lists and other sources searched or consulted to identify studies. Specify the date when each source was last searched or consulted.	6
Search strategy	7	present the full search strategies for all databases, registers and websites, including any filters and limits used.	Appendix pp 4-7
Selection process	8	Specify the methods used to decide whether a study met the inclusion criteria of the review, including how many reviewers screened each record and each report retrieved, whether they worked independently, and if applicable, details of automation tools used in the process.	6
Data collection process	9	Specify the methods used to collect data from reports, including how many reviewers collected data from each report, whether they worked independently, any processes for obtaining or confirming data from study investigators, and if applicable, details of automation tools used in the process.	6
Data items	10a	List and define all outcomes for which data were sought. Specify whether all results that were compatible with each outcome domain in each study were sought (e.g. for all measures, time points, analyses), and if not, the methods used to decide which results to collect.	6-7
	10b	List and define all other variables for which data were sought (e.g. participant and intervention characteristics, funding sources). Describe any assumptions made about any missing or unclear information.	NA
Study risk of bias assessment	11	Specify the methods used to assess risk of bias in the included studies, including details of the tool(s) used, how many reviewers assessed each study and whether they worked independently, and if applicable, details of automation tools used in the process.	6
Effect measures	12	Specify for each outcome the effect measure(s) (e.g. risk ratio, mean difference) used in the synthesis or presentation of results.	6-7
Synthesis methods	13a	Describe the processes used to decide which studies were eligible for each synthesis (e.g. tabulating the study intervention characteristics and comparing against the planned groups for each synthesis (item #5)).	6-7
	13b	Describe any methods required to prepare the data for presentation or synthesis, such as handling of missing summary	6-7

Section and Topic	Item #	Checklist item	Location where item is reported
		statistics, or data conversions.	
	13c	Describe any methods used to tabulate or visually display results of individual studies and syntheses.	6-7
	13d	Describe any methods used to synthesize results and provide a rationale for the choice(s). If meta-analysis was performed, describe the model(s), method(s) to identify the presence and extent of statistical heterogeneity, and software package(s) used.	6-7
	13e	Describe any methods used to explore possible causes of heterogeneity among study results (e.g. subgroup analysis, meta-regression).	6-7
	13f	Describe any sensitivity analyses conducted to assess robustness of the synthesized results.	6-7
Reporting bias assessment	14	Describe any methods used to assess risk of bias due to missing results in a synthesis (arising from reporting biases).	6-7
Certainty assessment	15	Describe any methods used to assess certainty (or confidence) in the body of evidence for an outcome.	6-7
RESULTS			
Study selection	16a	Describe the results of the search and selection process, from the number of records identified in the search to the number of studies included in the review, ideally using a flow diagram.	20
	16b	Cite studies that might appear to meet the inclusion criteria, but which were excluded, and explain why they were excluded.	20
Study characteristics	17	Cite each included study and present its characteristics.	Appendix pp 9-16
Risk of bias in studies	18	Present assessments of risk of bias for each included study.	Appendix pp 9-16
Results of individual studies	19	For all outcomes, present, for each study: (a) summary statistics for each group (where appropriate) and (b) an effect estimate and its precision (e.g. confidence/credible interval), ideally using structured tables or plots.	NA
Results of syntheses	20a	For each synthesis, briefly summarise the characteristics and risk of bias among contributing studies.	NA
	20b	Present results of all statistical syntheses conducted. If meta-analysis was done, present for each the summary estimate and its precision (e.g. confidence/credible interval) and measures of statistical heterogeneity. If comparing groups, describe the direction of the effect.	Tables 1-2, Figures 2-4; appendix pp 17-49
	20c	Present results of all investigations of possible causes of heterogeneity among study results.	13, Appendix pp 17-49
	20d	Present results of all sensitivity analyses conducted to assess the robustness of the synthesized results.	Appendix pp 17-49
Reporting biases	21	Present assessments of risk of bias due to missing results (arising from reporting biases) for each synthesis assessed.	NA
Certainty of evidence	22	Present assessments of certainty (or confidence) in the body of evidence for each outcome assessed.	Tables 1-2, Figures 2-4;

Section and Topic	Item #	Checklist item	Location where item is reported
			appendix pp 17-49
DISCUSSION			
Discussion	23a	Provide a general interpretation of the results in the context of other evidence.	11-13
	23b	Discuss any limitations of the evidence included in the review.	13
	23c	Discuss any limitations of the review processes used.	13
	23d	Discuss implications of the results for practice, policy, and future research.	13-14
OTHER INFORMATION			
Registration and protocol	24a	Provide registration information for the review, including register name and registration number, or state that the review was not registered.	5
	24b	Indicate where the review protocol can be accessed, or state that a protocol was not prepared.	5
	24c	Describe and explain any amendments to information provided at registration or in the protocol.	NA
Support	25	Describe sources of financial or non-financial support for the review, and the role of the funders or sponsors in the review.	3,9,15
Competing interests	26	Declare any competing interests of review authors.	15
Availability of data, code and other materials	27	Report which of the following are publicly available and where they can be found: template data collection forms; data extracted from included studies; data used for all analyses; analytic code; any other materials used in the review.	15

REFERENCES

1 Mrcela D, Markic J, Zhao C, *et al.* Changes following the Onset of the COVID-19 Pandemic in the Burden of Hospitalization for Respiratory Syncytial Virus Acute Lower Respiratory Infection in Children under Two Years: A Retrospective Study from Croatia. *Viruses* 2022; **14**: 2746.

2 Torres-Fernandez D, Casellas A, Mellado MJ, Calvo C, Bassat Q. Acute bronchiolitis and respiratory syncytial virus seasonal transmission during the COVID-19 pandemic in Spain: A national perspective from the pediatric Spanish Society (AEP). *J Clin Virol* 2021; **145**: 105027.

3 Mira-Iglesias A, Demont C, López-Labrador FX, *et al.* Role of age and birth month in infants hospitalized with RSV-confirmed disease in the Valencia Region, Spain. *Influenza Other Respir Viruses* 2022; **16**: 328–39.

4 Li Y, Wang X, Blau DM, *et al.* Global, regional, and national disease burden estimates of acute lower respiratory infections due to respiratory syncytial virus in children younger than 5 years in 2019: a systematic analysis. *The Lancet* 2022; **399**: 2047–64.

5 Atwell J, Hartman R, Parker D. RSV Among American Indian and Alaska Native Children: 2019 to 2020. *Pediatrics*.

6 Hartman R, Lutz C, Sutcliffe C. Incidence of Respiratory Syncytial Virus (RSV)-Associated Hospitalization Among American Indian and Alaska Native Children. Belfast, Ireland, 2022.

7 Caini S, Stolyarov K, Sominina A, *et al.* A comparative analysis of the epidemiology of influenza and respiratory syncytial virus in Russia, 2013/14 to 2018/19. *J Glob Health* 2022; **12**: 04009.

8 Casalegno J-S, Ploin D, Cantais A, *et al.* Characteristics of the delayed respiratory syncytial virus epidemic, 2020/2021, Rhône Loire, France. *Euro Surveill* 2021; **26**. DOI:10.2807/1560-7917.ES.2021.26.29.2100630.

9 Huang QS, Wood T, Jelley L, *et al.* Impact of the COVID-19 nonpharmaceutical interventions on influenza and other respiratory viral infections in New Zealand. *Nat Commun* 2021; **12**: 1001.

10 Chiu SS, Cowling BJ, Peiris JSM, Chan ELY, Wong WHS, Lee KP. Effects of Nonpharmaceutical COVID-19 Interventions on Pediatric Hospitalizations for Other Respiratory Virus Infections, Hong Kong - Volume 28, Number 1—January 2022 - Emerging Infectious Diseases journal - CDC. DOI:10.3201/eid2801.211099.

11 Reyes Domínguez AI, Pavlovic Nesic S, Urquía Martí L, Pérez González M del C, Reyes Suárez D, García-Muñoz Rodrigo F. Effects of public health measures during the SARS-CoV-2 pandemic on the winter respiratory syncytial virus epidemic: An interrupted time series analysis. *Paediatric and Perinatal Epidemiology* 2022; **36**: 329–36.

12 Foley DA, Phuong LK, Peplinski J, *et al.* Examining the entire delayed respiratory syncytial virus season in Western Australia. *Arch Dis Child* 2022; **107**: 517–9.

13 Lee CY, Wu TH, Fang YP, *et al.* Delayed respiratory syncytial virus outbreak in 2020 in Taiwan was correlated with two novel RSV-A genotype ON1 variants. *Influenza Other Respir Viruses* 2022; **16**: 511–20.

14 Guitart C, Bobillo-Perez S, Alejandre C, *et al.* Bronchiolitis, epidemiological changes during the SARS-CoV-2 pandemic. *BMC Infect Dis* 2022; **22**: 84.

15 Meyer M, Ruebsteck E, Eifinger F, *et al.* Morbidity of Respiratory Syncytial Virus (RSV) Infections: RSV Compared With Severe Acute Respiratory Syndrome Coronavirus 2 Infections in Children Aged 0-4 Years in Cologne, Germany. *J Infect Dis* 2022; **226**: 2050–3.

16 Pappa S, Haidopoulou K, Zarras C, *et al.* Early initiation of the respiratory syncytial virus season in 2021-2022, Greece. *J Med Virol* 2022; **94**: 3453–6.

17 Hernández-Rivas L, Pedraz T, Calvo C, Juan IS, José Mellado M, Robustillo A. Respiratory syncytial virus outbreak DURING THE COVID-19 PANDEMIC. How has it changed? *Enferm Infecc Microbiol Clin* 2021; published online Dec 22. DOI:10.1016/j.eimc.2021.12.003.

18 Fourgeaud J, Toubiana J, Chappuy H, *et al.* Impact of public health measures on the post-COVID-19 respiratory syncytial virus epidemics in France. *Eur J Clin Microbiol Infect Dis* 2021; **40**: 2389–95.

19 Lin W-H, Wu F-T, Chen Y-Y, *et al.* Unprecedented outbreak of respiratory syncytial virus in Taiwan associated with ON1 variant emergence between 2010 and 2020. *Emerg Microbes Infect* 2022; **11**: 1000–9.

20 Saravanos GL, Hu N, Homaira N, *et al.* RSV Epidemiology in Australia Before and During COVID-19. *Pediatrics* 2022; **149**: e2021053537.

21 Camporesi A, Morello R, Ferro V, *et al.* Epidemiology, Microbiology and Severity of Bronchiolitis in the First Post-Lockdown Cold Season in Three Different Geographical Areas in Italy: A Prospective, Observational Study. *Children (Basel)* 2022; **9**: 491.

22 Bermúdez Barrezueta L, Matías Del Pozo V, López-Casillas P, Brezmes Raposo M, Gutiérrez Zamorano M, Pino Vázquez M de la A. Variation in the seasonality of the respiratory syncytial virus during the COVID-19 pandemic. *Infection* 2022; **50**: 1001–5.

23 Loconsole D, Centrone F, Rizzo C, *et al.* Out-of-Season Epidemic of Respiratory Syncytial Virus during the COVID-19 Pandemic: The High Burden of Child Hospitalization in an Academic Hospital in Southern Italy in 2021. *Children (Basel)* 2022; **9**: 848.
